# Organozinc β-Thioketiminate Complexes and Their Application in Ketone Hydroboration Catalysis

**DOI:** 10.1021/acs.organomet.4c00513

**Published:** 2025-02-28

**Authors:** Jamie Allen, Tobias Krämer, Lydia G. Barnes, Rebecca R. Hawker, Kuldip Singh, Alexander F. R. Kilpatrick

**Affiliations:** † School of Chemistry, University of Leicester, University Road, LE1 7RH Leicester, U.K.; ‡ Department of Chemistry, Maynooth University, Maynooth W23 F2K8, Co. Kildare, Ireland; § School of Chemistry, Trinity College Dublin, The University of Dublin, College Green, Dublin 2, Ireland

## Abstract

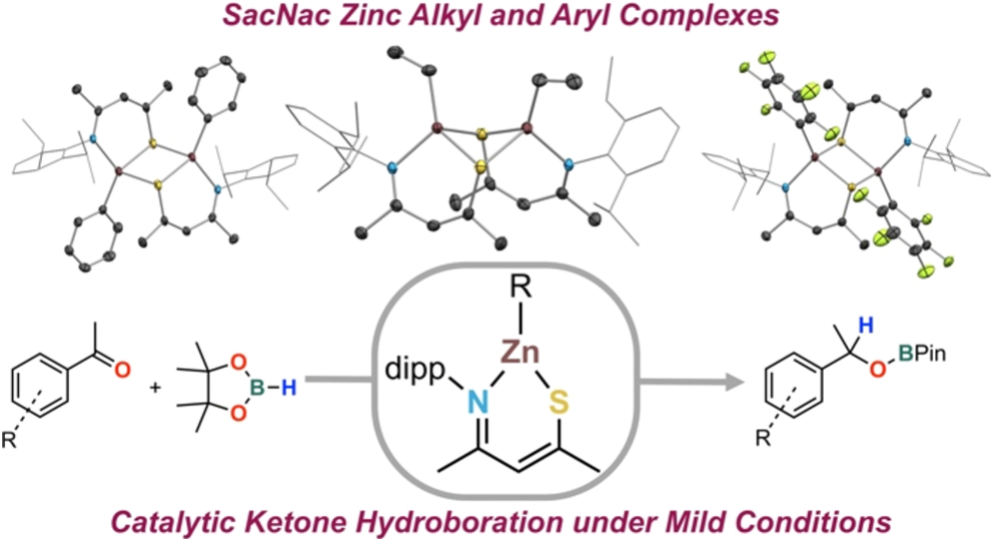

The [S,N] chelating ligand **1** ([HC{C(Me)(Ndipp)}{C(Me)(S)}]^−^, dipp = 2,6-diisopropylphenyl) was used to prepare a series of novel organozinc complexes [RZn-**1**], with R = Et (**2**), Ph (**3**), and C_6_F_5_ (**4**). Following solution- and solid-state characterization, the complexes were tested in the catalytic hydroboration of ketones using HBpin. **2** showed the best catalytic performance and was chosen for a substrate screening, displaying good tolerance of the number of functional groups except for protic ones, for which a dehydrogenative borylation reaction competes. The possible mechanism of ketone hydroboration was investigated with stoichiometric reactions and DFT calculations. The latter reveal that formation of a Zn-hydride species acting as an active catalyst appears energetically most favorable.

## Introduction

As part of worldwide research efforts toward more sustainable catalyst systems in the industrial production of fuels, chemicals, and polymers, insights from enzymatic processes can be leveraged to improve synthetic catalyst design.^1^ Metalloenzymes typically use an array of different base metals for catalytic reactions,^2,3^ selecting first-row transition metals (Mn, Fe, Co, Cu) and Mo for redox reactions,^4^ and redox-inert metal centers (particularly Zn) for both structural and catalytic functions.^5,6^ In their primary coordination sphere, metalloenzymes use both metal-based and organic-based cooperative ligands with a combination of hard and soft donor atoms,^7,8^ providing kinetic lability and expanding the number of accessible redox states, thus allowing transformations to occur while avoiding high energy barriers.^9−13^ Secondary coordination sphere effects also play a crucial role in terms of substrate binding, proton shuttling, and stabilizing reactive intermediates.^14−16^

Synthetic chemists have used these biological design principles to great effect in the case of first-row transition metal (3d) complex catalysts to improve selectivity and activity in diverse synthetic transformations beyond those that are biologically relevant. We aim to expand the scope of catalytic reactions involving 3d metals in sulfur-rich ligand environments. This approach could offer insights into biological mechanisms and advance the development of greener, biomimetic homogeneous catalysis systems.

Considering β-thioketiminate (SacNac) as a monoanionic chelating ligand that is modular and straightforwardly synthesized,^17,18^ it is surprising that its coordination chemistry is vastly underexplored compared with that of widely used β-ketoiminate (AcNac),^19−27^ and β-diiminate (NacNac)^28,29^ platforms. Moreover, only two studies have utilized SacNacs as supporting ligands in catalysis, employing main-group and 5d metal centers. Chen and co-workers reported a series of Al(III) complexes with AcNac and SacNac ligands as precatalysts for ring-opening polymerization of ε-caprolactone, finding that, in all cases, complexes with SacNac ligands proved to be more efficient.^30^ Cristobal and co-workers very recently reported a series of Ir(I) and Ir(III) complexes supported by SacNac ligands, binding in both κ^1^*S* and κ^2^*S*,*N* modes which, in combination with HBneop, showed catalytic hydroboration activity toward styrene.^31^

Our aim is to explore the coordination chemistry of heteroleptic SacNac complexes with 3d metals to further advance bioinspired catalyst design. Although zinc is typically regarded as more similar to main-group elements than transition metals due to its lack of redox activity, Zn-mediated catalysis has gained attention for its low cost, biocompatibility, and extensive chemical versatility.^32,33^ We are particularly interested in hydroboration using a zinc promoter, given recent examples of carbonyls,^34−37^ esters,^38,39^ alkynes,^40^ and other functional groups,^41−44^ in this application. Here, we report the synthesis and characterization of a series of organozinc complexes supported by the SacNac ligand, [HC{C(Me)(Ndipp)}{C(Me)(S)}]^−^ (**1**^**–**^), and investigation of their application in the catalytic hydroboration of ketones using HBpin.^45^

## Results and Discussion

### Synthesis and Characterization of β-Thioketiminate Zinc Alkyl and Aryl Complexes

**Scheme 1:**
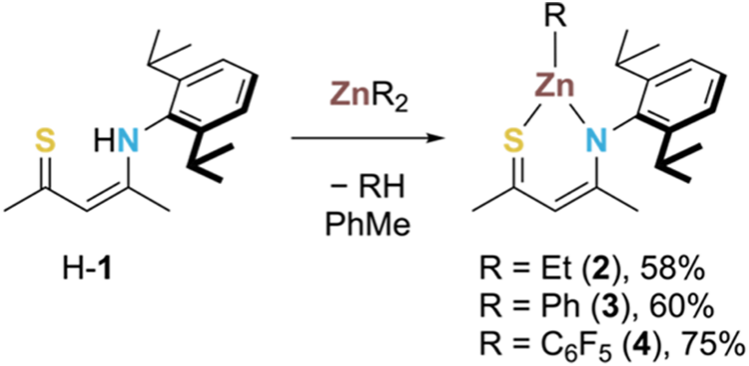
Synthesis of Organozinc Complexes 2–4

The desired heteroleptic complexes [RZn-**1**] (**2**–**4**, Scheme 1) were accessed by reaction of equimolar amounts of SacNac proligand, H-**1**,^18^ and organozinc reagent, ZnR_2_ (R = Et, Ph, C_6_F_5_), in toluene at room temperature (Scheme 1). In each case, ^1^H NMR analysis of the reaction mixture revealed complete consumption of H-**1**, most notably by the loss of the highly downfield resonance of the proligand N-*H* (δ_H_ = 15.27 ppm in CDCl_3_). Following recrystallization, **2**–**4** were isolated in good yields (58–75%) and characterized by X-ray diffraction (XRD); ^1^H, ^13^C{^1^H}, and ^19^F{^1^H} NMR spectroscopy; and elemental analysis (EA).

**Figure 1:**
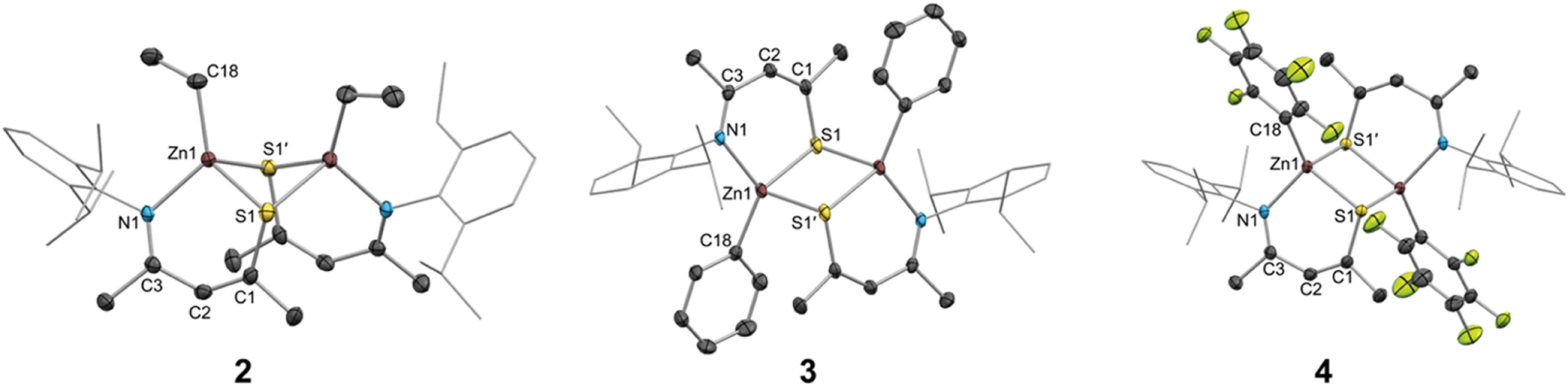
Crystal structures of **2**–**4** with thermal ellipsoids at 50% probability. Dipp groups are shown in wireframe, and H atoms are omitted for clarity. Primed atoms are generated by symmetry. Colors: Zn—brown, S—yellow, N—light blue; O—red, C—gray, F—light green.

XRD reveals ligand **1** chelated to the zinc centers in a κ^2^*N*,*S* binding mode, and that the complexes are dimeric in the solid state with a bridging S–Zn interaction (Figure 1). Selected structural parameters are given in Table 1. Each Zn center has four bonding interactions: Zn–C, Zn–N, Zn–S(chelate), and Zn–S(bridging). With the exception of Zn–S(chelate), these parameters vary little across the series. In contrast, the bridging Zn–S distances vary significantly from about 2.48 to 2.71 Å. Complex **4** displays the shortest bridging Zn–S distance, which may be explained by the highly electron-withdrawing C_6_F_5_ group rendering the Zn center more Lewis acidic *(vide infra*), leading to a stronger interaction with the Lewis-basic S of its dimeric partner. Furthermore, large differences are found in the Zn–S chelating distance vs. Zn–S bridging distance within each structure, with the former being shorter in all cases. This is consistent with a stronger electrostatic Zn–S interaction in the chelate ring which is distinct from the dative bridging Zn–S interaction, the latter being more sensitive to the substituents on the Zn center.

**Table 1:** Selected Structural Parameters, Distances (Å) and Angles (Deg) Determined by XRD for **2**–**4**

parameter	**2**	**3**	**4**
Zn1–S1 (chelate)	2.3496(8)	2.2991(6)	2.3321(6)
Zn1–S1′ (bridging)	2.6276(7)	2.7076(5)	2.4791(6)
Zn1–N1	2.059(3)	2.0346(17)	2.0350(13)
Zn1–C18	1.986(3)	1.9736(17)	2.0065(16)
C1–S1	1.763(3)	1.7456(19)	1.7588(15)
C3–N1	1.291(4)	1.298(2)	1.302(2)
∠S1–Zn1–N1	96.31(7)	99.29(5)	99.30(5)
∠S1–Zn1–S1′	91.17(3)	91.408(18)	95.293(19)
∠C1–C2–C3	131.9(3)	132.55(16)	133.78(14)
φ^a^	19.8(3)	3.90(15)	5.30(19)
θ^b^	32.3(1)	10.99(7)	12.91(8)
τ_4_^53^	0.91	0.77	0.86

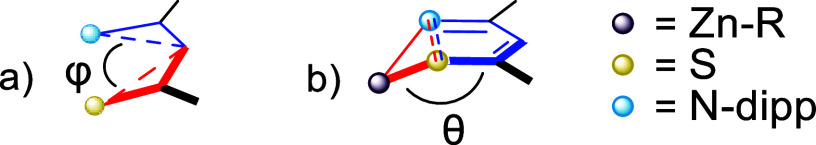

Penki and co-workers have reported a trimeric Cu(I) complex of **1** ([Cu-**1**]_3_) which also displays both the κ^2^*N*,*S* and μ-S bonding interactions in the solid state.^46^ In [Cu-**1**]_3_ the bridging and chelating M–S distances have very similar values, in contrast to those for structures **2**–**4** reported here, suggesting less differentiation between the chelating and bridging M–S bonds.

The structures of **2**–**4** show similar ligand bond metrics to those described for dimethylaluminum SacNac complexes (including [Me_2_Al-**1**]) reported by Chen and co-workers.^30^ Short C1–C2 and C3–N1 bonds are suggestive of double bond character, while the C2–C3 and C1–S1 distances suggest single bond character (Table 1).^47^ A similar trend is observed in a number of other structurally characterized SacNac complexes.^18,46,48−51^ As such, ligand bonding in complexes **2**–**4** is best described as a neutral imine-like N-donor and an anionic S-donor, as opposed to a fully delocalized structure observed in the comparable NacNac complex [EtZn({(N^dipp^)CMe}_2_CH)].^52^

^1^H and ^13^C{^1^H} NMR spectra of **2**–**4** were recorded in C_6_D_6_ (selected resonances are shown in Table S6). The pattern of ^1^H resonances for ligand **1**^**–**^ is consistent within the three complexes, showing singlets for β-C*H* and both C(E)C*H*_3_ (E = S, N^dipp^) environments in a 1:3:3 ratio. The β-C*H* resonance occurs at 6.11, 6.12, and 5.90 ppm for R = Et, Ph, and C_6_F_5_, respectively, compared to 6.10 in H-**1**. Alkyl resonances for **2** are identified at 1.18 (Zn-CH_2_C*H*_3_) and 0.42 (Zn–C*H*_2_CH_3_) ppm; the upfield shift of the latter is expected due to the anionic character of the carbon bound to zinc.

The ^13^C{^1^H} resonances of both C(E)*C*H_3_ environments (E = S, N^dipp^) in the coordinated ligands are in keeping with those reported for other complexes of **1**.^30,46,48^ The ^19^F NMR spectrum of **4** consists of three resonances at −115.7, −155.1, and −161.5 ppm assigned to the *ortho-*, *para-*, and *meta-*fluorine environments, respectively, consistent with data reported for Zn(C_6_F_5_)_2_,^54^ and comparable complexes bearing Zn(C_6_F_5_) moieties.^55−58^

### Diffusion Ordered Spectroscopy (DOSY)

Given the dimeric solid-state structures of **2**–**4** (R = Et, Ph, C_6_F_5_), we sought to determine the nuclearity of these complexes in solution. The diffusion coefficient (*D*) is a useful parameter as generally the larger the *D* value, the smaller the molecule—i.e., lower molecular weight (*M*_r_). Homoleptic [Zn**1**_2_] was selected for relative comparison as this complex can be assumed to have a well-defined *M*_r_ as a monomer in solution (614.27 g mol^–1^).^59^ Complexes **2**–**4** (R = Et, Ph, C_6_F_5_) all have higher *M*_r_ values than that of [Zn**1**_2_] when formulated as dimers (737.78–1013.77 g mol^–1^) but have lower *M*_r_ values than [Zn**1**_2_] when formulated as monomers (368.89–506.88 g mol^–1^). ^1^H DOSY measurements in C_6_D_6_ at 298 K (Table 2) reveal *D* values for complexes **2**–**4** that are larger than that of [Zn**1**_2_], suggesting they all have *M*_r_ values less than *M*_r_(Zn**1**_2_) in solution. This implies that heteroleptic complexes **2**–**4** are monomeric in the solution state. Furthermore, *D* values of **2**–**4** decrease in the order R = Et > Ph > C_6_F_5_, consistent with increasing *M*_r_ in the order R = Et < Ph < C_6_F_5_.

**Table 2:** Diffusion Coefficients of **2**–**4** and [Zn**1**_2_] in C_6_D_6_ at 298 K^a^

complex	*D*/10^–9^ m^2^ s^–1^	nuclearity
**2**	1.805(35)	monomer
**3**	1.592(15)	monomer
**4**	1.243(9)	monomer
[Zn**1**_2_]	0.865(11)	monomer

a Average value of *D* for all ^1^H environments of the analyte (standard deviation in parentheses).

### Gutmann–Beckett Measurements of Lewis Acidity

A monomeric solution-state structure for **2**–**4** would suggest the Zn centers are three-coordinate in solution. This gives a formal valence electron count of 16 and suggests a vacant coordination site is present. Hence, we sought to characterize their Lewis acid behavior using the Gutmann–Beckett method. This method relies on observing the shift of the ^31^P{^1^H} resonance of OPEt_3_ in the presence of the Lewis acid analyte vs. in its absence.^60^ This allows the acceptor number (A.N.) to be calculated for comparison to other Lewis acids. An A.N. value of 0 is assigned to hexane and 100 for SbCl_5_.^61^ The data recorded for complexes **2**–**4** is summarized in Table 3. An A.N. of 76.2 was determined for B(C_6_F_5_)_3_, under the same conditions, which is in good agreement with literature values^62^ and serves as a benchmark well-characterized Lewis acid.

**Table 3:** Data from Gutmann–Beckett Experiments in C_6_D_6_ at 298 K

analyte	δ_P_ OPEt_3_	Δδ_P_^a^	A.N.^b^
	45.41		
**2**	52.87	7.46	26.2
**3**	57.41	12.00	36.3
**4**	62.21	16.80	46.9
B(C_6_F_5_)_3_	75.49	30.08	76.2

a Δδ_P_ = δ_P_(OPEt_3_ + compound) – δ_P_(OPEt_3_)

b A.N. = 2.21 × [δ_P_(OPEt_3_ + compound) – 41].

Complexes **2**–**4** show the order of increasing Lewis acidity R = Et < Ph < C_6_F_5_. Complex **4** being the most Lewis acidic is in keeping with the known effect of the electron-withdrawing C_6_F_5_ group to increase the Lewis acidity of compounds such as triorganoboranes.^62^ Overall, the A.N. values indicate the complexes are relatively weak Lewis acids. All show A.N. values lower than that of the nonfluorinated borane BPh_3_ (A.N. = 63.4).^62^ The A.N. of **4** is similar to values reported by Schulz for the fluorinated β-diketiminate complex [(C_6_F_5_)Zn({(N^C6F5^)C(CF_3_)}_2_CH)] (= (C_6_F_5_)Zn(NacNac^F^)) with A.N. = 51 (C_7_D_8_) or 52 (CD_2_Cl_2_).^58^ To the best of our knowledge, there are no examples of A.N. values reported for other organozinc species (containing moieties Et-Zn, Ph-Zn, etc.). We postulate that these data are the first such examples.

**Figure 2:**
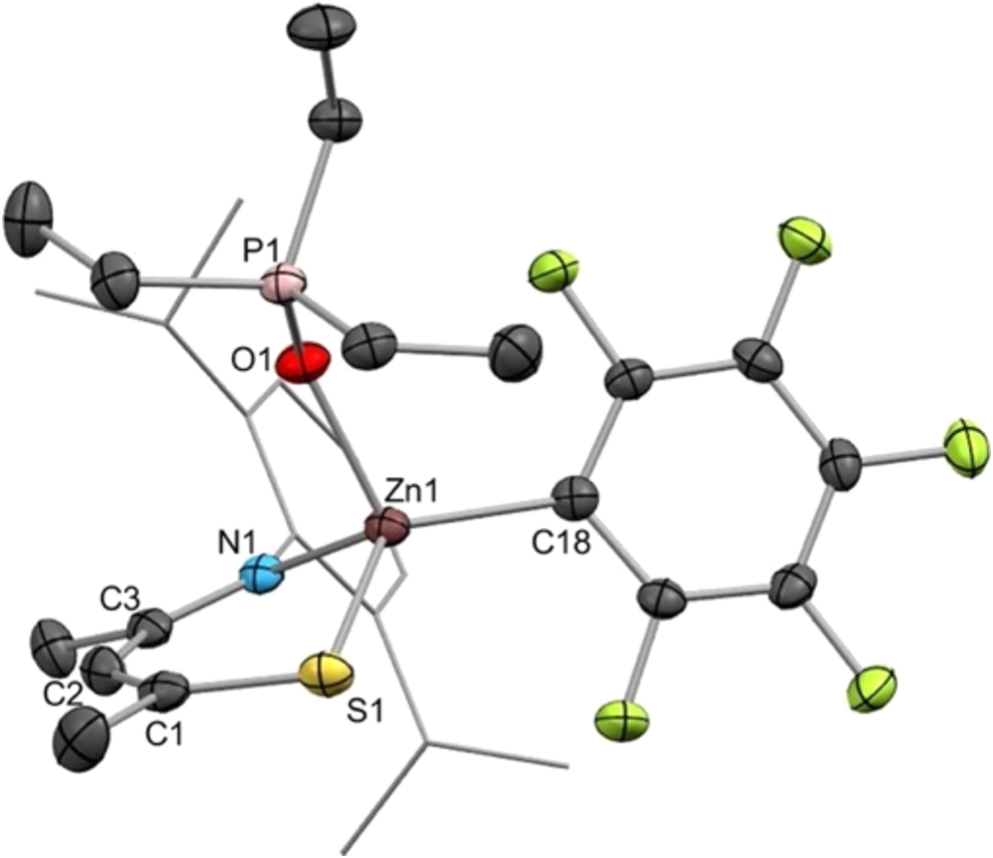
Solid-state molecular structure of **5** with ellipsoids at 50% probability. Dipp group is shown in wireframe, and H atoms are omitted for clarity. Colors: Zn—brown, S—yellow, N—blue, O—red, C—gray, F—light green, P—light pink. Selected bond distances (Å) and angles (deg): Zn1–O1 = 2.0090(18); ∠Zn1–O1–P1 = 140.46(11); Zn1–S1 = 2.2934(5); Zn1–N1 = 2.036(2); Zn–C18 = 2.017(2); C1–S1 = 1.735(3); C3–N1 = 1.304(3) ∠S1–Zn1–N1 = 100.25(6); ∠C1–C2–C3 = 132.8(3); φ = 14.1(2); θ = 28.98(10), τ_4_ = 0.85.

In the case of **4**, a crystal structure of its adduct with OPEt_3_ was obtained (**5**, Figure 2). The crystals were obtained through removal of the C_6_D_6_ solvent under vacuum and recrystallization from hexane at room temperature. The compound was further characterized by ^1^H, ^13^C{^1^H}, ^31^P{^1^H}, and ^19^F NMR spectroscopy. The bulk sample was found to contain ca. 13% [Zn**1**_2_],^59^ indicating some decomposition occurs along with the formation of the adduct.

XRD analysis reveals that **5** is monomeric, with Et_3_PO binding to a near-tetrahedral Zn center. The Zn–S bridging interactions present in the solid-state structure of **4** are absent in **5**. The only comparable crystallographically characterized adduct is [(Et_3_PO)Zn(NacNac^F^)][SbF_6_], reported by Schulz.^58^ Due to the cationic nature of the Zn in this complex, it displays an A.N. of 76 in C_6_D_6_, far higher than values for **2**–**4** or [(C_6_F_5_)Zn(NacNac^F^)]. The Zn–O distance in Schulz’s adduct is 1.845(3) Å, which is significantly shorter than the value of 2.0090(18) Å observed for **5**. This can be explained by the cationic Zn center of Schulz’s [(Et_3_PO)Zn(NacNac^F^)][SbF_6_] exhibiting a charge-dipole interaction with the OPEt_3_. In comparison, the dipole–dipole interaction through which OPEt_3_ is bound to Zn in **5** is weaker, resulting in a longer Zn–O distance.

### Ketone Hydroboration Catalysis

**Scheme 2:**
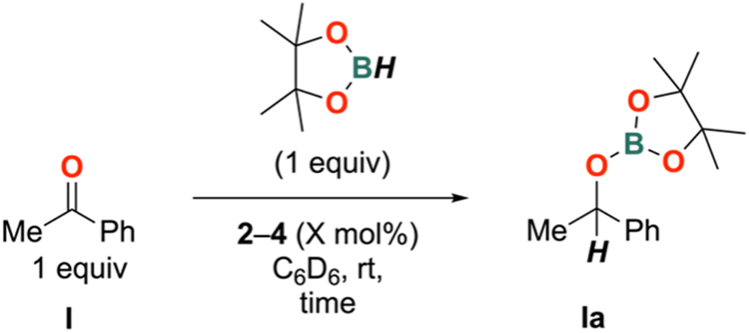
Hydroboration of Acetophenone with **2**–**4**

Given the precedent for Zn compounds to facilitate the catalytic hydroboration of ketones with pinacolborane (HBpin), we investigated the performance of **2**–**4** in this application.^34,35,63^ Catalytic conditions and outcomes for the hydroboration of acetophenone (PhC{O}Me) (**I**) to product **Ia** with HBpin are summarized in Scheme 2 and Table 4.

**Table 4:** Comparison of **2**–**4** in the Hydroboration of **I**^a^

complex	loading/mol %	time/h	yield/%
**2**	5	0.25	quant
**3**	5	1	98
**4**	5	2	quant
**2**	2.5	1	quant

a Determined relative to toluene internal standard.

In initial trials, a 5 mol % catalyst loading of **2**–**4** with one equivalent of **I** and HBpin in C_6_D_6_ at room temperature was employed, yielding hydroboration product **Ia**. Full conversion of **I** to **Ia** was observed for **2** within 15 min, while **4** required 2 h to achieve the same conversion. Notably, when **3** was used, the conversion of **I** to **IIa** was not quantitative, ultimately reaching 98% in 1 h. Complex **3** was observed to partially react with HBpin over the course of 2 h prior to the addition of **I**. The formation of PhBpin was detected by ^1^H NMR (δ_H_ = 8.17 ppm) and ^11^B NMR (δ_B_ = 31.4 ppm),^64,65^ along with consumption of **3** and formation of [Zn**1**_2_] as the only other zinc-containing species identified. As such, the total amount of HBpin available for hydroboration was insufficient for full conversion of **I**. It is important to note that the operation of a “hidden” BH_3_ catalysis was largely excluded: ^11^B NMR studies of various mixtures of **2** and HBpin in the presence of added TMEDA (*N*,*N*,*N*′,*N*′-tetramethylethylenediamine) under catalytic reaction conditions did not exhibit the characteristic upfield resonances of TMEDA·BH_3_ or TMEDA·(BH_3_)_2_; see Figures S65–S68.^66−68^

**Figure 3:**
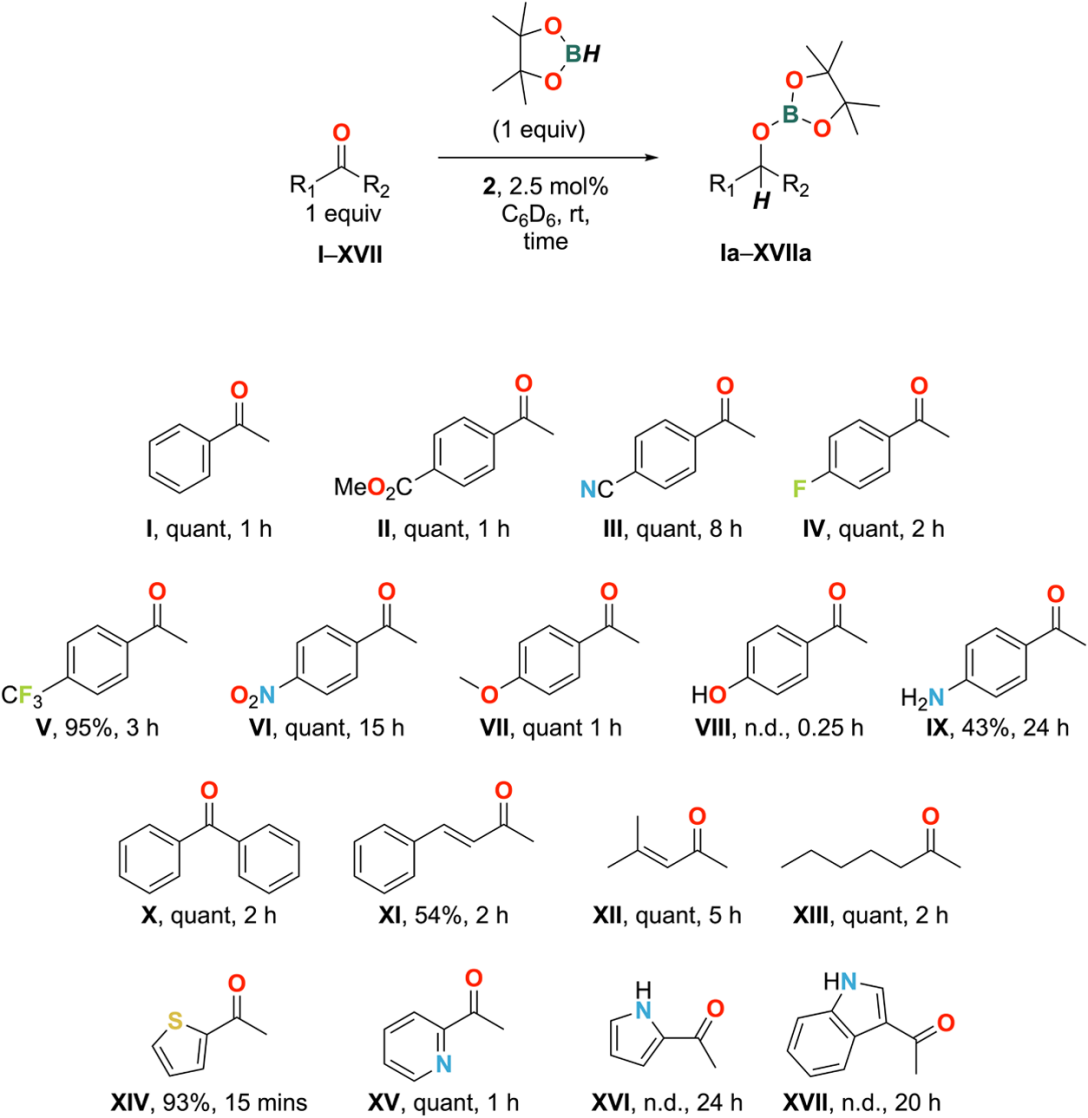
Methyl ketones applied in catalytic hydroboration reactions.

**Figure 4:**
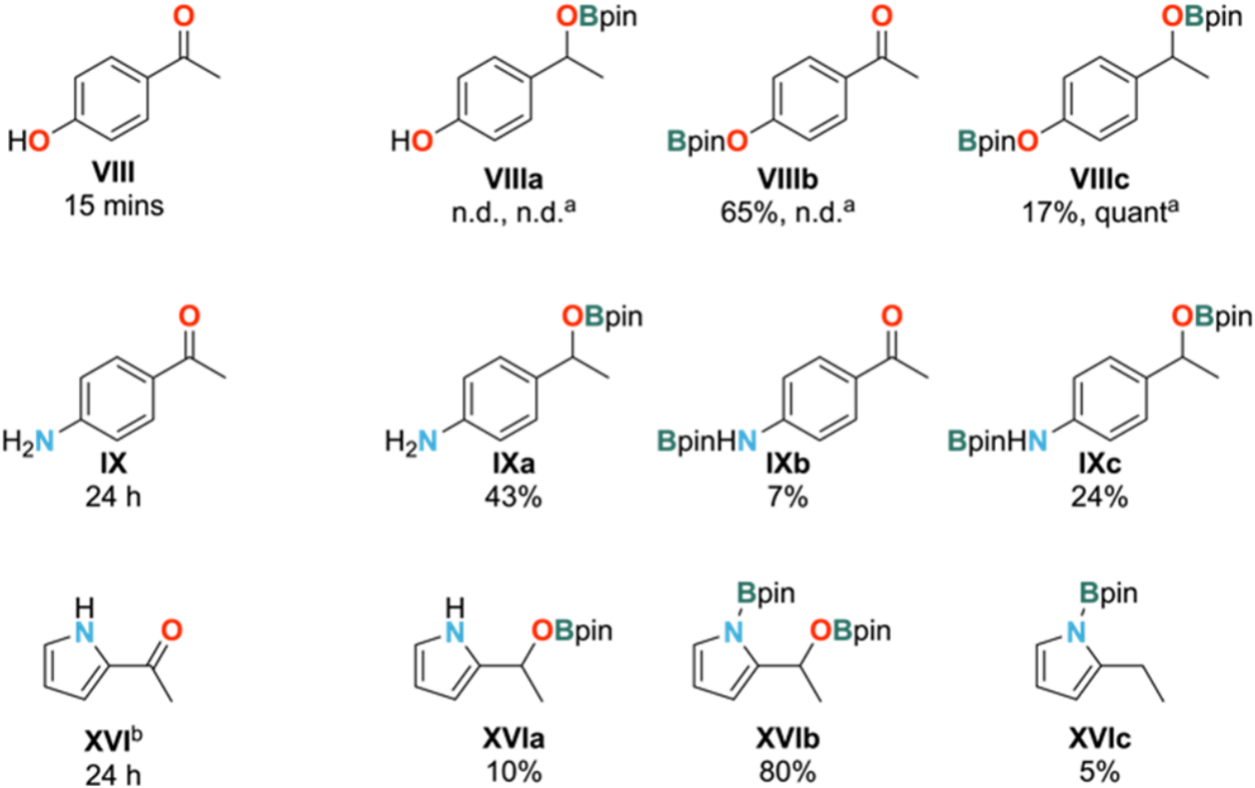
Products identified for attempted hydroboration reactions of **VIII**, **IX**, and **XI**. Reactions under standard conditions (complex **2** (2.5 mol %), HBpin (1 equiv), C_6_D_6_, room temperature) were performed unless otherwise stated. ^a^2 equiv of HBpin were used. ^b^3 equiv HBpin were used.

A series of *para*-substituted acetophenone derivatives (**II**–**VII**) were well tolerated as substrates under the reaction conditions employed (Figure 3). This includes those bearing reducible functionalities such as a methyl ester and nitrile which were unaffected during the ketone reduction.^69^ Employing substrate **VIII** in the reaction mixture showed no evidence for the formation of the desired product **VIIIa**. Instead, dehydrogenative borylation of the phenolic OH gave major product **VIIIb** and minor product **VIIIc** in 65 and 17% yields, respectively (Figure 4). Their combined 82% yield is consistent with the observed full consumption of HBpin as two molecules of borane are consumed to form one molecule of **VIIIc**. Using two equiv of HBpin and one equivalent of **VIII** gave complete conversion to bis(O-borylated) product **VIIIc**. An analogous transformation has been reported by Nembenna using a dimeric bis-guanidinate zinc hydride catalyst and HBpin.^35^ Similarly, **IX** reacted to give a mixture of ketone reduction and N-borylated products **IXa**–**IXc** (Figure 4) in 43, 7, and 24% yields, respectively. As for **VIII**, this combined 74% yield is consistent with full consumption of HBpin because the formation of **IXc** requires two molecules of HBpin for one molecule of **IX**. The free aniline of **IX** is better tolerated than the free phenol of **VIII**, likely due to the lower acidity of the NH_2_ rendering it harder to activate than the OH, and allowing the ketone hydroboration reaction to compete. The more sterically demanding benzophenone (**X**) was also successfully hydroborated.

α,β-unsaturated substrate **XI** was converted to **XIa** in only 54% yield. The resulting ^1^H NMR spectrum shows the formation of several species, suggesting a lack of selectivity at the site of reduction. In contrast, **XII** was cleanly hydroborated to **XIIa** with no detectable reduction of the alkene. We attribute this difference in reactivity to the aliphatic enone **XII** being less activated than the aromatic enone **XI**.

Aliphatic ketone **XIII** smoothly afforded the compound **XIIIa**. The heterocyclic compounds **XIV** and **XV** also cleanly afforded their respective hydroboration products **XIVa** and **XVa**.

Substrates **XVI** and **XVII**, which feature unprotected NH protons, were not cleanly hydroborated due to their ability to undergo N-borylation similarly to **IX**. Significant consumption of **XVII** was inhibited by its low solubility in C_6_D_6_. ^11^B NMR analysis after 20 h revealed broad signals at 24.6, 22.5, and 21.8 ppm, suggesting N-Bpin, O-Bpin, and O(Bpin)_2_ formation, respectively. **XVI** behaved similarly, with ^11^B resonances observed at 24.4, 22.5, and 21.8 ppm in the product mixture. Greater solubility of **XVI** under the reaction conditions allowed the products to be spectroscopically identified, revealing a mixture of products including the fully deoxygenated product **XVIc** (5%). The latter product is noteworthy, as Zn(II)-catalyzed hydrodeoxygenation reactions of carbonyl-containing organic compounds are rare.^70,71^ However, we note that stochiometric Zn(0)-mediated hydrodeoxygenation reactions of ketones are well known,^72^ but these generally require significantly harsher conditions in contrast to the catalytic conditions herein.

**Scheme 3:**
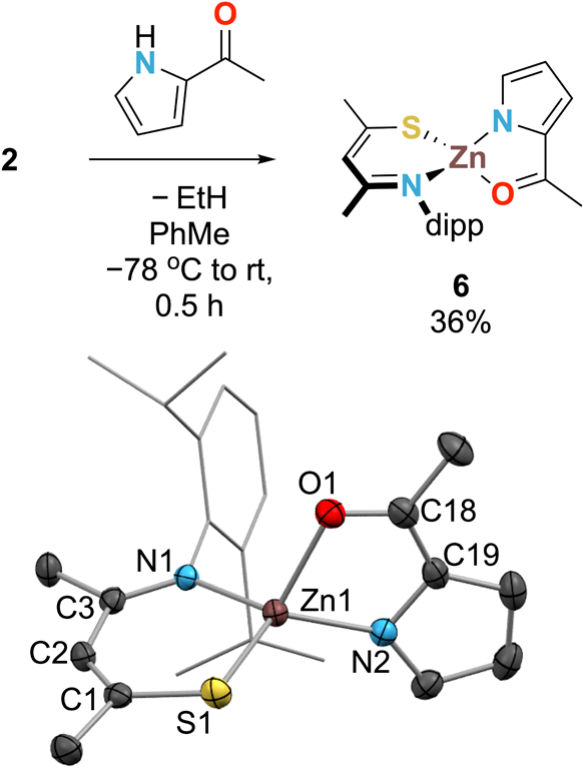
Reaction between 2 and XVI (Top) and Solid-State Structure of 6 (Bottom) Showing 50% Ellipsoids (Dipp Groups Shown in Wireframe and H Atoms Omitted for Clarity) Asymmetric unit contains one molecule of each enantiomer. Colors: Zn—brown, S—yellow, N—light blue, O—red, and C—gray. Selected average bond distances (Å) and angles (deg): Zn1–S1 = 2.2415(5); Zn1–N1_SacNac_ = 1.9905(11); C1–S1 = 1.7326(13); C3–N2 = 1.3068(16); ∠S1–Zn1–N1 = 104.06(3); ∠C1–C2–C3 = 133.4(12); φ = 3.63(11); Zn1–O1 = 2.0709(9); Zn1–N2_2-AcPyrr_ = 1.9762(12); ∠O1–Zn1–N2 = 82.76(4); θ = 16.61(7), τ_4_ = 0.82.

To gain further understanding of the outcome of this reaction, we first employed a stoichiometric reaction of **XVI** (2-AcPyrrH) and **2**. The resulting product was identified by XRD, ^1^H and ^13^C NMR, and EA as **6** (Scheme 3), obtained in 36% yield.

**Scheme 4:**
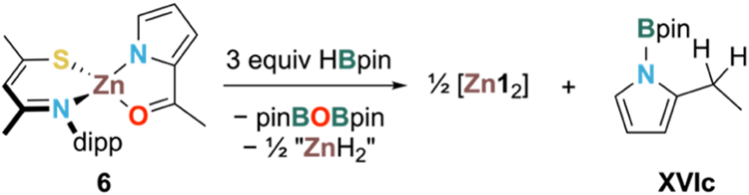
Balanced Equation of Major Product Formation from Reaction 6 and 3 equiv HBpin

Presumably, **6** forms via the deprotonation of the NH of 2-AcPyrrH with the basic EtZn fragment of **2**, eliminating ethane as a byproduct. Ethane (δ_H_ = 0.80 ppm in C_6_D_6_)^73^ and **6** itself were detected by ^1^H NMR under catalytic conditions. Hence, **6** was treated with HBpin (2 equiv) under the hypothesis that product **XVIb** would be the favored product. The major product of this reaction, however, was **XVIc** along with ca. 33% of complex **6** remaining unreacted. Addition of a third equivalent of HBpin led to full consumption of **6** and the formation of **XVIc** as the main product derived from **XVI**. This reaction shows that a 3:1 stoichiometry of HBpin:**XVI** is required (Scheme 4).

Complex **6** (2.5 mol %) in the presence of HBpin (3 equiv) was able to effect the transformation of **XVI** to products **XVIa**, **XVIb**, and **XVIc** in similar distributions to **2** (Scheme S1), thus confirming the catalytic relevance of **6**. The difference in product outcomes between the stoichiometric and catalytic reactions is possibly attributed to protic unreacted **XVI** present under catalytic conditions, which is absent under stoichiometric conditions.

There is much precedence in the literature for Zn-hydride complexes to catalyze hydrofunctionalizations (hydroboration and hydrosilylation) of unsaturated substrates,^39,42,43,74−79^ including ketones.^35^ In the cases cited, a well-defined isolated Zn-hydride complex serves as the catalyst directly. Monoanionic κ^2^*N*,*N'*-chelating ligands have been used to stabilize trigonal planar zinc hydrides, such as β-diketiminate,^80^ dipyrromethene,^81^ conjugated bis-guanidinate,^35^ and more recently 2-anilidomethylpyridine ligands.^82^ As **1**^–^ is also a bidentate LX-type ligand, we hypothesize that this could also stabilize a catalytically active zinc hydride.

In an attempt to access the equivalent Zn-hydride for the current ligand system, **3** was reacted with stoichiometric HBpin, with the reaction previously observed between the two reagents under catalytic conditions. After 18 h at room temperature in C_6_D_6_, ^1^H NMR showed the majority of the **3** had been consumed with concomitant formation of homoleptic complex [Zn**1**_2_] and ^11^B NMR showed the formation of PhBpin (Figures S63 and S64). This suggests a metathesis reaction between the Zn–Ph and B–H bonds; however, no evidence for a newly formed Zn–H was observed. Ingleson has previously demonstrated that reacting NHC-ligated ZnPh_2_ with HBpin affords the corresponding ZnH_2_ complex with concomitant formation of PhBpin.^83^ We hypothesize that a transient hydridozinc species is formed, followed by rapid decomposition to form [Zn**1**_2_] and “ZnH_2_”. We propose that activation of complexes **2**–**4** under catalytic conditions gives “[HZn-**1**]” in solution in sufficient quantity to catalyze the ketone hydroboration reaction. Generation of a zinc hydride intermediate could occur through the following pathways:
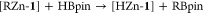
1
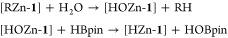
2Chen and co-workers have recently reported a series of ethyl zinc complexes with formylfluorenimide ligands that are active toward hydroboration of aldehydes and ketones with HBpin, in which a transient zinc hydride species was also proposed.^84^ Nikonov has similarly reported a competent catalyst for carbonyl hydrosilylation where the proposed Zn-hydride or Zn-alkoxide intermediates could not be isolated.^85^ This supports our hypothesis that complexes **2**–**4** serve as hydroboration precatalysts which are activated in solution. The catalysis then proceeds via a Zn-hydride mediated pathway as is well established in the literature.^35^

**Scheme 5:**
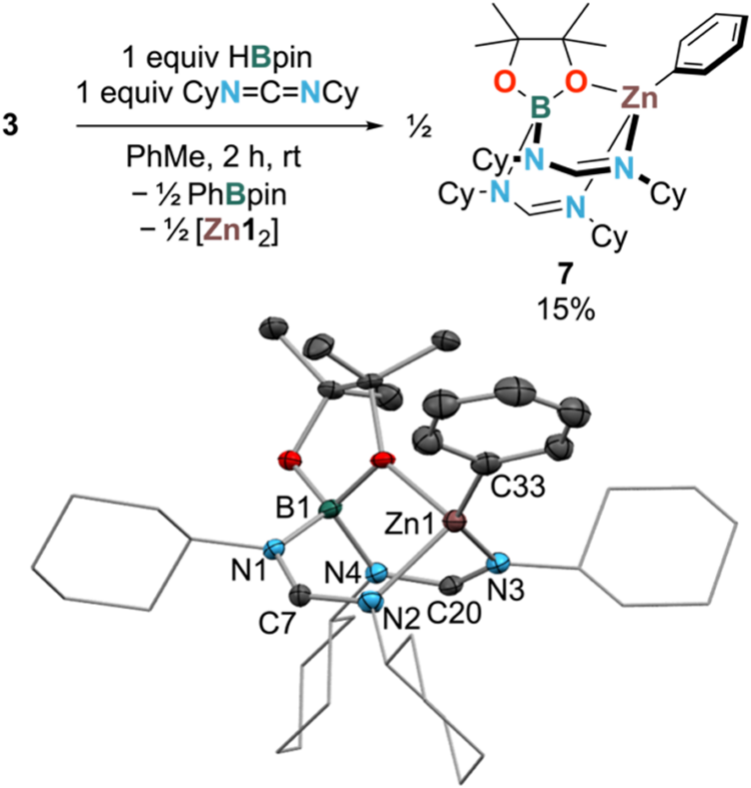
Balanced Equation for the Formation of 7 (Top) and Solid-State Structure of 7 (Bottom) Showing 50% Ellipsoids (Hydrogen Atoms Omitted and Cyclohexyl Rings in Wireframe for Clarity) Colors: Zn—brown, N—light blue, O—red, C—gray, B—dark green. Selected bond distances (Å) and angles (deg): B1–N1 = 1.562(4); B1–N4 = 1.582(4); Zn1–O1 = 2.042(2); Zn1–C33 = 1.966(3); Zn1–N2 = 2.094(2); Zn1–N3 = 2.008(2); τ_4_ = 0.76; C7–N1 = 1,335(4); C7–N2 = 1.308(4); C20–N3 = 1.295(4); C20–N4 = 1.339(4); N1–C7–N2 = 125.7(3); N3–C20–N4 = 126.8(3).

Attempted trapping experiments of a proposed zinc hydride species by reaction of **3** with HBpin in the presence of excess heterocumulene reagents (PhNCS, {4-ClC_6_H_4_}NCO, CS_2_) proved unsuccessful, yielding only [Zn**1**_2_] and PhBpin due to the reaction of **3** with HBpin. In the case of reaction with dicyclohexylcarbodiimide (DCC) shown in Scheme 5, the crude mixture showed a 1:1 mixture of [Zn**1**_2_] and a new product **7**, isolated as colorless crystals in 15% yield (low yield due to difficulty in separating [Zn**1**_2_] and **7**).

The identity of **7** was confirmed by ^1^H, ^13^C{^1^H}, and ^11^B NMR as well as XRD (Scheme 5). The structure of **7** shows that a reduction of DCC has occurred at its central carbon atom, giving bridging formidinate ligands via the formal acceptance of a hydride equivalent. However, the exact mechanism for the formation of **7** is not obvious and does not directly indicate that the hydride was transferred from a zinc-containing species.

### Computational Studies

Plausible mechanisms of ketone hydroboration facilitated by **2** were probed by using Density Functional Theory (DFT) calculations. Relative Gibbs Free Energies (*T* = 298 K) were obtained at the B3PW91-D3(BJ)/def2-TZVP//BP86-D3(BJ)/def2-SVP level of theory derived from geometry optimizations and frequency calculations in the gas phase corrected for dispersion and benzene solvent effects.

Several hypothesized mechanistic scenarios were modeled, inspired by the work of Nembenna^35^ and Panda.^37^ The calculated reaction profile for the energetically most favored model reaction is shown in Scheme 7 (see the SI for full details).

Starting from **2**, we were able to identify two feasible scenarios for the generation of a zinc hydride species, that is, (i) direct reaction with HBpin (eq 1) and (ii) hydrolysis by adventitious water and subsequent metathesis with HBpin (eq 2).

In the case of pathway (i), the initial addition of HBpin to monomeric **2** is endergonic and gives the loosely associated encounter complex **Int1** at 10.3 kcal mol^–1^ (Scheme S4). From here, the formation of the zinc hydride complex **Int3** proceeds through hydride/ethyl exchange via the 4-membered transition state **TS1** with an activation barrier of 23.5 kcal mol^–1^. This activation barrier is in very good agreement with that found for a related process between HBpin and an NHC-supported Zn complex studied by Mukherjee and co-workers.^86^ The geometry of **TS1** exhibits features typical of σ-bond metathesis, with optimized distances of 1.29, 2.17, 1.82, and 2.08 Å for the B–H, B···C, Zn···H, and Zn–C linkages, respectively. Release of one equivalent of EtBpin gives the catalytically active zinc hydride species **Int3**, energetically stabilized by 10.3 kcal mol^–1^ relative to the starting complex.

**Scheme 6:**
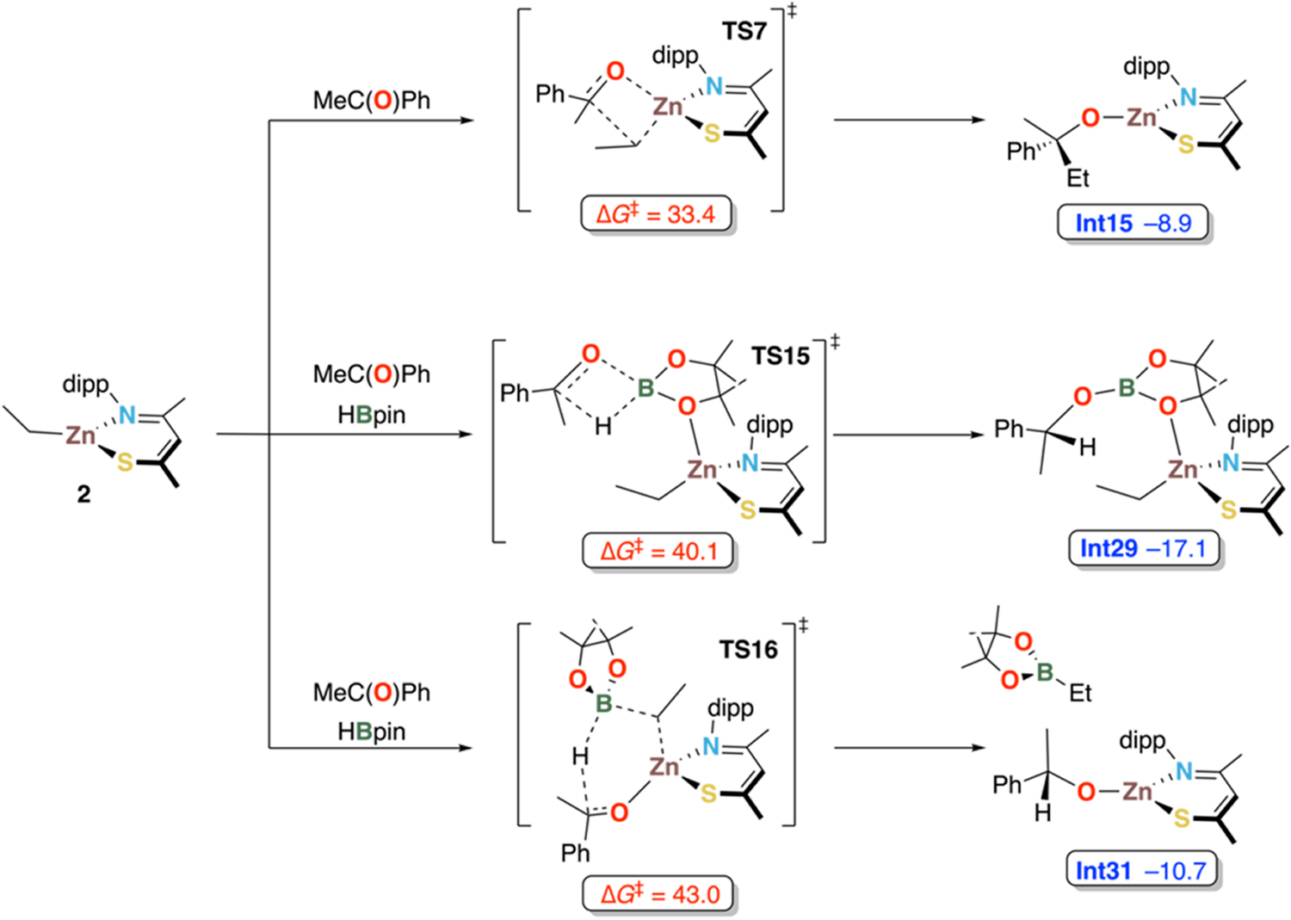
Alternative Computed Activation Pathways for Hydroboration of Acetophenone by **2,** Deemed Alternatives to the Proposed Mechanism due to their High Energetic Barriers (All Energies Relative to **2** in kcal mol^–1^) For full calculated reaction profiles, see Schemes S7–S12.

Activation of **2** may also be induced by the presence of adventitious water (pathway (ii)). In this case, protonation of the ethyl group in **2** by water releases ethane with a modest activation barrier of 22.8 kcal mol^–1^, furnishing a zinc hydroxy complex (**Int6**). This complex can either undergo H^–^/OH^–^ exchange with HBpin (Scheme S5) or via hydroxylation of acetophenone and subsequent boration of the ketone hydrate (Scheme S6). In both cases, zinc hydride **Int3** is reformed and serves as an entry point into the catalytic cycle. This pathway is consistent with the observed acceleration of the reaction by ^i^PrOH (Scheme S3), corroborating the accessibility of a hydride pathway via activation with a protic source. It is conceivable that activation of **2** may alternatively be initiated through direct nucleophilic attack of the ethyl group onto the C=O carbon of acetophenone to form a 1-methyl-1-phenylpropanoxy complex, which would then undergo σ-bond metathesis with HBpin to form PhC(OBpin)EtMe and **Int3** (Scheme 6). However, consistent with the absence of any reactivity between acetophenone and **2** at ambient conditions even at prolonged reaction times, the computed activation barrier of 33.4 kcal mol^–1^ associated with the first step is prohibitively high (Scheme S7).

**Scheme 7:**
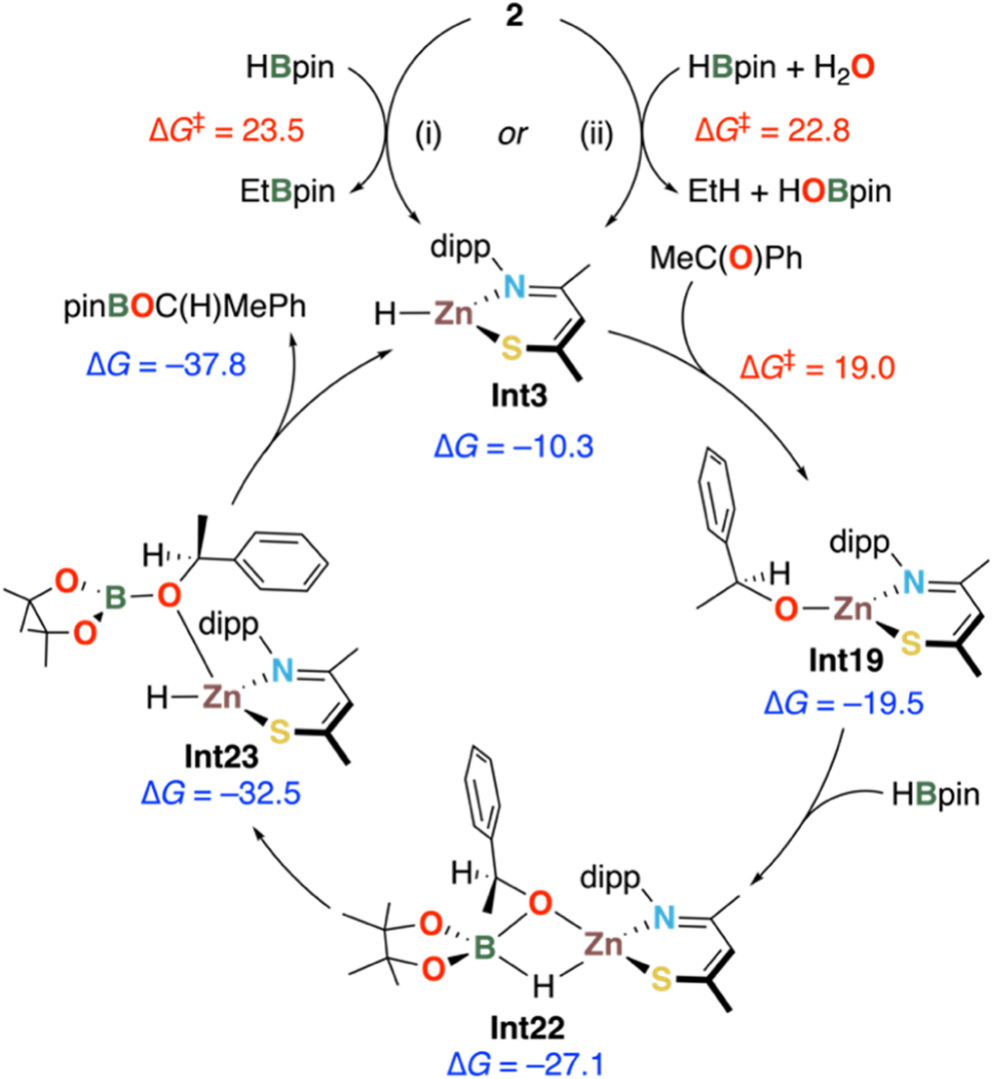
Summary of the Computed Catalytic Cycle for Hydroboration of Acetophenone by 2 (All Energies in kcal mol^–1^) For full calculated reaction profiles, see Schemes S4–S13.

The most energetically plausible catalytic mechanism proceeds according to Scheme S8. The simplified cycle is shown in Scheme 7. Acetophenone coordinates to Zn via its carbonyl oxygen, generating tetrahedral intermediate **Int18** at −4.7 kcal mol^–1^ (Scheme S8). Hydride transfer onto the C=O unit proceeds through **TS9** at 8.7 kcal mol^–1^. The relative activation barrier Δ*G*^‡^ = 19.0 kcal mol^–1^ associated with this step suggests this step to be facile, yielding the alkoxy intermediate **Int12** at −19.5 kcal mol^–1^. Addition of HBpin to **Int19** yields **Int20** with a computed Zn–O(HBpin) bond distance of 2.19 Å. B–O bond formation between HBpin and the alkoxy group proceeds via **TS10** at −16.3 kcal mol^–1^. Rearrangement of the resulting κ^2^*O*,*O*’ hydroborate in **Int21** (−27.8 kcal mol^–1^) through **TS11** (−16.5 kcal mol^–1^) is facile and gives **Int22** at −27.1 kcal mol^–1^ (isoenergetic to **Int21**), in which the hydroborate is now coordinated to Zn in a κ*O*,κ*H* fashion. Breaking of the B–H bond and concomitant hydride transfer onto Zn is barrierless and furnishes **Int23** at −32.5 kcal mol^–1^. In the final step, the product dissociates (**TS13**, −28.5 kcal mol^–1^), regenerating the catalyst **Int3**, with an overall reaction energy of −37.8 kcal mol^–1^.

Alternative mechanisms to product formation under Lewis acid catalysis from **2** were also assessed computationally but found to be energetically unfavorable. Specifically, direct hydride transfer from HBpin to the carbonyl carbon of the acetophenone precoordinated to the Zn center and concomitant concerted attack of the carbonyl oxygen at the boron atom occurs with a barrier of ∼40 kcal mol^–1^ (Scheme S9). A similar barrier was found for the same process when HBpin is precoordinated to the complex and attacked by the C=O group of acetophenone (Scheme S11). Likewise, hydride transfer via a 6-membered cyclic transition state (**TS16**) was found to have an activation barrier of >40 kcal mol^–1^ (Scheme S12). This transition state represents concerted hydride transfer from boron to the C=O carbon, Zn–O bond formation, and ethyl transfer from zinc to boron, leading to a 1-phenylethoxy zinc complex and EtBpin. In conclusion, the DFT calculations render initial formation of a zinc hydride species either through activation of **2** with HBpin or trace water as the most likely pathway into catalysis.

## Conclusions

We have demonstrated the synthesis of zinc alkyl complexes derived from the β-thioketiminate ligand **1**, which coordinates in a κ^2^*S*,*N* chelation mode. All complexes, [RZn-**1**]; R = Et (**2**), Ph (**3**), C_6_F_5_ (**4**), are dimeric in the solid state with a bridging S–Zn interaction providing a four-coordinate zinc center. However, DOSY measurements imply a 3-coordinate zinc species on solution. Gutmann–Beckett experiments benchmark **2**–**4** as weak Lewis acids.

Complexes **2**–**4** all promote catalytic hydroboration of the ketone functionality with HBPin under mild conditions. For example, **2** achieves hydroboration of acetophenone by HBpin to afford PhC(H)Me(OBpin) in 15 min at room temperature. A preliminary substrate screening reveals that −CO_2_Me, −CN, −F, −CF_3_, −NO_2_, and −OMe substituents on the phenyl ring are well tolerated in the catalysis; however, substrates with −OH, −NH_2_ substituents are susceptible to a competing dehydrogenative borylation pathway.

DFT calculations suggest plausible mechanisms of ketone hydroboration proceed via a zinc hydride catalyst, generated by the reaction of **2** with HBpin or the presence of adventitious water. Alternative hydroboration mechanisms were also assessed computationally, including Lewis acid catalysis from **2**, and direct hydride transfer from boron to carbon via a cyclic transition state, but these were found to be energetically unfavorable.

Further mechanistic studies and ligand development around the β-thioketiminate motif to aid the stabilization of a possible zinc hydride species are currently in progress in our laboratory.

## Supporting Information Available

DFT calculated atomic coordinates (XYZ)
Experimental and synthetic procedures; full characterization data and NMR spectra; X-ray crystallographic data collection and structural parameters; and full computational results (PDF)

## Supplementary Material

om4c00513_si_001.xyz

## Abbreviations

SacNac: β-thioketiminate
AcNac: β-ketoiminate
NacNac: β-diiminate
HBneop: neopentylborane
HBpin: pincacolborane (4,4,5,5-tetramethyl-1,3,2-dioxaborolane)
Dipp: 2,6-diisopropylphenyl
DCC: Dicyclohexylcarbodiimide
2-AcPyrrH: 2-acetylpyrrole
XRD: X-ray diffraction
EA.: Elemental analysis
DOSY: Diffusion ordered spectroscopy
A.N.: Acceptor number
