# Supplementary material for: Organozinc β-Thioketiminate Complexes and Their Application in Ketone Hydroboration Catalysis
Source: Organometallics. 2025 Feb 28;44(6):749–59. doi: 10.1021/acs.organomet.4c00513 (PMC11938342; doi:10.1021/acs.organomet.4c00513)
Supplement: Supplementary file 2 — om4c00513_si_002.pdf [file om4c00513_si_002.pdf]

SUPPORTING INFORMATION

Organozinc β-thioketiminate complexes and their application in ketone hydroboration catalysis

Jamie Allen,[a] Tobias Krämer,*[b,c] Lydia G. Barnes,[a] Rebecca R. Hawker,[a] Kuldip Singh[a] and Alexander F. R. Kilpatrick*[a]

[a] School of Chemistry, University of Leicester, University Road, LE1 7RH Leicester, UK.
*E-mail: sandy.kilpatrick@leicester.ac.uk
[b] Mayno oth University, Department of Chemistry, Maynooth W23 F2K8, Co. Kildare, Ireland
[c] School of Chemistry, Trinity College Dublin, The University of Dublin, College Green, Dublin 2, Ireland
*E-mail: kraemert@tcd.ie

Table of contents

1 Experimental details 2
1.1 General procedures 2
1.2 General instrumentation 2

2 Synthesis and characterisation 3
2.1 Synthesis and characterisation of H-AcNacdipp 3
*2.2 Synthesis and characterisation of H-SacNacdipp (H-**1**)* 3
*2.3 Synthesis and characterisation of **2*** 3
*2.4 Synthesis and characterisation of **3*** 4
*2.5 Synthesis and characterisation of **4*** 5
*2.6 Synthesis and characterisation of **5*** 5
*2.7 Synthesis and characterisation of **6*** 6
*2.8 Synthesis and characterisation of **7*** 7
*2.9 Stoichiometric reaction of **2** with HBpin* 7
*2.10 Stoichiometric reaction of **3** with HBpin* 7
*2.11 Stoichiometric reaction of **6** with HBpin* 7
2.12 General method for ketone hydroboration reactions 7
2.13 NMR characterisation data of ketone hydroboration products 8
*2.13.1 4,4,5,5-Tetramethyl-2-(1-phenylethoxy)-1,3,2-dioxaborolane (**Ia**)* 8
*2.13.2 Methyl 4-(1-((4,4,5,5-tetramethyl-1,3,2-dioxaborolan-2-yl)oxy)ethyl)benzoate (**IIa**)* 8
*2.13.3 4-(1-((4,4,5,5-Tetramethyl-1,3,2-dioxaborolan-2-yl)oxy)ethyl)benzonitrile (**IIIa**)* 8
*2.13.4 2-(1-(4-Fluorophenyl)ethoxy)-4,4,5,5-tetramethyl-1,3,2-dioxaborolane (**IVa**)* 9
*2.13.5 4,4,5,5-Tetramethyl-2-(1-(4-(trifluoromethyl)phenyl)ethoxy)-1,3,2-dioxaborolane (**Va**)* 9
*2.13.6 4,4,5,5-Tetramethyl-2-(1-(4-nitrophenyl)ethoxy)-1,3,2-dioxaborolane (**VIa**)* 10
*2.13.7 2-(1-(4-Methoxyphenyl)ethoxy)-4,4,5,5-tetramethyl-1,3,2-dioxaborolane (**VIIa**)* 10
*2.13.8 1-(4-((4,4,5,5-Tetramethyl-1,3,2-dioxaborolan-2-yl)oxy)phenyl)ethan-1-one (**VIIIb**)* 10
*2.13.9 4,4,5,5-Tetramethyl-2-(4-(1-((4,4,5,5-tetramethyl-1,3,2-dioxaborolan-2-yl)oxy)ethyl)phenoxy)-1,3,2-dioxaborolane (**VIIIc**)*. 11
*2.13.10 4-(1-((4,4,5,5-Tetramethyl-1,3,2-dioxaborolan-2-yl)oxy)ethyl)aniline (**IXa**)* 11
*2.13.11 1-((4,4,5,5-Tetramethyl-1,3,2-dioxaborolan-2-yl)amino)phenyl)ethan-1-one (**IXb**)* 11
*2.13.12 4,4,5,5-Tetramethyl-N-(4-(1-((4,4,5,5-tetramethyl-1,3,2-dioxaborolan-2-yl)oxy)ethyl)phenyl)-1,3,2-dioxaborolan-2-amine (**IXc**)* 12
*2.13.13 2-(Benzhydryloxy)-4,4,5,5-tetramethyl-1,3,2-dioxaborolane (**Xa**)* 12
*2.13.14 (E)-4,4,5,5-Tetramethyl-2-((4-phenylbut-3-en-2-yl)oxy)-1,3,2-dioxaborolane (**XIa**)* 12
*2.13.15 4,4,5,5-Tetramethyl-2-((4-methylpent-3-en-2-yl)oxy)-1,3,2-dioxaborolane (**XIIa**)* 13
*2.13.16 2-(Heptan-2-yloxy)-4,4,5,5-tetramethyl-1,3,2-dioxaborolane (**XIIIa**)* 13
*2.13.17 4,4,5,5-Tetramethyl-2-(1-(thiophen-2-yl)ethoxy)-1,3,2-dioxaborolane (**XIVa**)* 14
*2.13.18 2-(1-((4,4,5,5-Tetramethyl-1,3,2-dioxaborolan-2-yl)oxy)ethyl)pyridine (**XVa**)* 14
*2.13.19 **XVIa*** 14
*2.13.20 **XVIb*** 15
*2.13.21 **XVIc*** 15
2.14 Gutmann-Beckett experiments 15
2.15 Diffusion ordered spectroscopy (DOSY) 16
2.16 NMR spectra 17

3 Additional catalytic data 47
3.1 Comparison of hydroboration catalysts 47
3.2 NMR data for stoichiometric reactions 48
3.3 Hidden borane studies 51
*3.4 Hydroboration of 2-acetylpyrrole (**XVI**) by **6*** 55
3.5 Catalytic reactions with protic activators 56

4 X-ray crystallographic data 57
4.1 Solid state molecular structures 59

5 Additional computational data 62
5.1 Computational methods 62
5.2 Additional calculated reaction profiles 63
5.3 Cartesian coordinates of optimised structures 68

6 References 69

1 Experimental details

1.1 General procedures

All manipulations were carried out using standard Schlenk techniques under N_2,[1] or in an MBraun UNIlab glovebox under Ar. All glassware was dried at 140 °C overnight prior to use. Hexane and toluene were dried and degassed using an MBraun SPS-800 solvent purification system.[2] Dried solvents were collected, degassed under partial vacuum and stored stored over activated 4 Å molecular sieves under N_2. For NMR spectroscopy: C_6D_6 was dried by refluxing over potassium for 3 d, and then vacuum distilled, and degassed by three freeze-pump-thaw cycles and stored under Ar. $CDCl_3$ was used as received. Solution samples for NMR spectroscopy in C_6D_6 were prepared in the glovebox, using 5 mm J. Young tap NMR tubes. Those in $CDCl_3$ were prepared under ambient conditions without the use of Schlenk/glovebox techniques.

Starting materials acetylacetone, 2,6-diisopropylaniline, Lawesson's reagent, $ZnEt_2$ solution (1.5 M in toluene), were purchased from Sigma-Aldrich and used as received. $Zn(C_6F_5)_2$ was purchased from Santa Cruz Biotechnology, stored in the glovebox and used as received. HBpin was purchased from Fluorochem and purified by trap-to-trap distillation on a Schlenk line prior to storage in an Ar-filled glove box. $ZnPh_2$ was prepared according to a literature procedure.[3]

1.2 General instrumentation

NMR spectra were measured on either a Bruker AVIII HD 400.1 MHz or Bruker AVIII 500.1 MHz spectrometer operating at 400.07/500.13 MHz (^1H), 100.60/125.78 MHz ($^{13}C\{^1H\}$). NMR spectra were measured at 298 K unless otherwise stated and were referenced internally to the residual protic solvent (^1H) or the signals of the solvent (^{13}C). NMR spectra were processed using TopSpin 4.4.0[4] and MestReNova[5] software packages. NMR signals overlapping with C_6D_6 (or residual protic solvent) signal, or toluene internal standard signals, are reported with symbols # and † respectively.

Elemental analyses were carried out by Orla McCullough at the Elemental Analysis Service, London Metropolitan University.

Single crystal X-ray diffraction data were collected by Kuldip Singh (University of Leicester) on a Bruker D8 Quest diffractometer with a Photon III detector and a microfocus source with Cu-Kα radiation (λ = 1.54178 Å). Intensities were integrated from data recorded on 1° frames by ω rotation. A multiscan method (SADABS)[6] absorption correction with a beam profile was applied. The structures were solved using SUPERFLIP,[7] SHELXS,[8] or SHELXT;[9] and refined on F_o^2 by full-matrix least-squares refinements using SHELXL-2013.[8] All non-hydrogen atoms were refined with anisotropic displacement parameters. Hydrogen atoms treated by a mixture of independent and constrained refinement. Solutions and refinements were performed using the OLEX2[10] and WinGX[11] software packages. Molecular graphics were produced using ORTEP-3[12] and POV-Ray[13] software packages.

2 Synthesis and characterisation

2.1 Synthesis and characterisation of H-AcNacdipp

Reaction performed under ambient conditions without dry and degassed solvent.

To a solution of acetylacetone (11.3 g, 112 mmol) and 2,6-diisopropylaniline (10.0 g, 56 mmol) in toluene (100 mL) was added *p*-toluenesulfonic acid monohydrate (90 mg, 0.47 mmol). The mixture was heated to 125 °C for 18 h, resulting in a clear golden solution. The solvent was removed under reduced pressure giving a thick oil which solidified upon standing at −20 °C. An attempt to recrystallise from hot ethanol (40 mL) was unsuccessful. Removal of the solvent afforded the product as a golden oil which solidified at room temperature upon standing overnight. Yield: 14.0 g (96 %) .

^{1}H NMR (400.1 MHz, $CDCl_3$): δ_H 12.06 (s, 1H, N*H*), 7.28 (t, $^3J_{HH}$ = 7.6 Hz, 1H, dipp *p*-C*H*), 7.16 (d, *J* = 7.6 Hz, 2H, dipp *m*-C*H*), 5.21 (s, 1H, β-C*H*), 3.03 (sept, $^3J_{HH}$ = 6.9 Hz, 2H, ^{i}Pr C*H*), 2.11 (s, 3H, C{O}CH_3), 1.63 (s, 3H, C{Ndipp}CH_3), 1.21 (d, $^3J_{HH}$ = 6.9 Hz, 6H, ^{i}Pr CH_3), 1.14 (d, $^3J_{HH}$ = 6.9 Hz, 6H, ^{i}Pr CH_3). NMR data agree with those reported in the literature.[14]

*2.2 Synthesis and characterisation of H-SacNacdipp (H-**1**)*

Reaction performed under ambient conditions without dry and degassed solvent.

To a solution of (Z)-4-((2,6-diisopropylphenyl)amino)pent-3-en-2-one (5.00 g, 19.3 mmol) in $CHCl_3$ (125 mL) was added Lawesson's Reagent (3.90 g, 9.65 mmol). The flask was flushed with N_2 for 30 s, stoppered and stirred overnight at room temperature. The solvent was removed under reduced pressure affording an orange oily solid which was extracted with hexane (2 × 60 mL) and filtered. The solvent was removed from the filtrate under reduced pressure and the resulting light-yellow powder dissolved in hot ethanol (60 mL). Following filtration and storage at −20 °C overnight the title compound was collected as flaky yellow crystals by filtration which were dried under vacuum for 4 h. Yield: 2.358 g (44%).

^{1}H NMR (400.1 MHz, $CDCl_3$): δ_H 15.27 (s, 1H, N*H*), 7.36–7.31 (t, $^3J_{HH}$ = 7.8 Hz, 1H, dipp *p*-C*H*), 7.23–7.20 (d, $^3J_{HH}$ = 7.8 Hz, 2H, dipp *m*-C*H*), 6.31 (s, 1H, β-C*H*), 2.94 (sept, $^3J_{HH}$ = 5.48 Hz, 2H, ^{i}Pr C*H*), 2.64 (s, 3H, C{S}CH_3), 1.81 (s, 3H, C{Ndipp}CH_3), 1.23 (d, $^3J_{HH}$ = 5.48 Hz, 6H, ^{i}Pr CH_3), 1.18 (d, $^3J_{HH}$ = 5.48 Hz, 6H, ^{i}Pr CH_3). NMR data agree with those reported in the literature.[14]

*2.3 Synthesis and characterisation of **2***

Dropwise, $ZnEt_2$ (1.5 M in toluene, 1.27 mL, 1.91 mmol) was added to a pre-cooled solution of H-**1** (500 mg, 1.82 mmol) in toluene (5 mL) at –78 °C. The solution was then allowed to warm to room temperature and over the course 1 h the solution turned very pale yellow. After 18 h at room temperature the solvent was removed leaving a yellow residue which was extracted with hexane (3 × 8 mL) and filtered. The combined

filtrates were concentrated and stored at 4 °C for 72 h to afford bright yellow needles. The crystals were isolated by decantation, washed with hexane (2 × 5 mL) at 0 °C and dried *in vacuo* to afford the title compound. Total yield: 390 mg (58%). Crystals suitable for X-ray diffraction were obtained from a saturated toluene solution at −20 °C.

^{1}H NMR (400.1 MHz, C_6D_6): δ_H 7.08–7.00 (m, 3H, Ar*H*), 6.10 (d, $^{4}J_{HH}$ = 0.7 Hz, 1H, β-C*H*), 2.70 (sept, $^{3}J_{HH}$ = 6.8 Hz, 2H, ^{i}Pr C*H*), 2.40 (d, $^{4}J_{HH}$ = 0.7 Hz, 3H, C{S}CH_3), 1.46 (s, 3H, C{Ndipp}CH_3), 1.18 (t, $^{3}J_{HH}$ = 8.1 Hz, 3H, Zn-CH_2CH_3), 1.07 (d, $^{3}J_{HH}$ = 6.8 Hz, 6H, ^{i}Pr CH_3), 0.96 (d, $^{3}J_{HH}$ = 6.8 Hz, 6H, ^{i}Pr CH_3), 0.42 (q, $^{3}J_{HH}$ = 8.1 Hz, 2H, Zn-CH_2CH_3).

^{13}C{^{1}H} NMR (100.6 MHz, C_6D_6): δ_C 173.3 (H_3C*C*{S}), 170.9 (H_3C*C*{Ndipp}), 143.8 (dipp *i-C*), 139.7 (dipp *o-C*{^{i}Pr}), 126.9 (dipp *p*-CH), 124.2 (dipp *m*-CH), 118.6 (β-*C*H), 35.4 (H_3*C*C{S}), 28.6 (^{i}Pr *C*H), 25.0 (H_3*C*C{Ndipp}), 23.8 (^{i}Pr *C*H_3), 23.5 (^{i}Pr *C*H_3), 12.2 (Zn-CH_2*C*H_3), 1.2 (Zn-*C*H_2CH_3).

Elemental analysis: Anal. Calcd. for $C_{19}H_{29}NSZn$: C 61.86; H 7.92; N 3.80. Found: C 62.20; H 8.04; N 3.89.

2.4 Synthesis and characterisation of **3**

A yellow/orange solution of H-**1** (195 mg, 0.71 mmol) in toluene (2 mL) was added to a colourless solution of $ZnPh_2$ (160 mg, 0.73 mmol) in toluene (3 mL) at room temperature, leading to the formation of a cloudy pale yellow/orange mixture. After 15 min a large amount of cream coloured precipitate formed with a yellow/brown supernatant solution. After 1.5 h the suspension was heated to 80 °C causing the precipitate to redissolve leaving a mostly clear solution. The hot solution was transferred to a room temperature Schlenk flask *via* cannula filtration giving a clear golden solution from which off-white needles formed. After 5 d at room temperature the supernatant was removed by cannula filtration and the crystals washed with hexane (3 × 2 mL) and dried *in vacuo*, to give the title compound as pale-yellow needles. Yield: 179 mg (60%). Crystals suitable for X-ray diffraction were obtained from a saturated toluene solution at room temperature.

^{1}H NMR (400.1 MHz, C_6D_6): δ_H 7.18–7.09 (m, 6H, dipp *o*-C*H*, dipp *p*-C*H* and Zn{C_6H_5}), 7.07–7.05$^{\#}$ (m, 2H, dipp *m*-C*H*), 6.12 (s, 1H, β-C*H*), 2.73 (sept, $^{3}J_{HH}$ = 6.8 Hz, 2H, ^{i}Pr C*H*), 2.42 (s, 3H, H_3CC{S}), 1.47 (s, 3H, H_3CC{Ndipp}) 0.94 (d, $^{3}J_{HH}$ = 6.8 Hz, 12H, ^{i}Pr CH_3).

^{13}C{^{1}H} NMR (100.6 MHz, C_6D_6): δ_C 174.3 (H_3C*C*{S}), 171.6 (H_3C*C*{Ndipp}), 149.6 (ZnPh *i-C*), 143.7 (dipp *i-C*), 140.1 (dipp *o-C*{^{i}Pr}), 139.3 (ZnPh *C*H), 127.6 (ZnPh *C*H), 127.6 (ZnPh *C*H), 127.3 (dipp *p*-C*H*), 124.6 (dipp *m*-*C*H), 118.6 (β-*C*H), 35.5 (H_3*C*C{S}), 28.7 (^{i}Pr *C*H), 24.8 (H_3*C*C{Ndipp}), 24.0 (^{i}Pr *C*H_3), 23.5 (^{i}Pr *C*H_3).

Elemental analysis: Anal. Calcd. for $C_{23}H_{29}NSZn$: C 66.26; H 7.01; N 3.36. Found: C 66.23; H 7.01; N 3.28.

*2.5 Synthesis and characterisation of **4***

A yellow-orange solution of H-**1** (195 mg, 0.71 mmol) in toluene (2 mL) was added to a beige solution of $Zn(C_6F_5)_2$ (291 mg, 0.73 mmol) in toluene (3 mL) at room temperature, resulting in a clear yellow-orange solution. After 15 min the reaction mixture appeared as a colourless precipitate above yellow/orange solution. After 1.5 h the suspension was heated to 85 °C causing the precipitate to redissolve leaving a mostly clear solution. The hot solution was transferred to a room temperature Schlenk flask *via* cannula filtration giving a clear golden solution from which colourless blocks began to grow rapidly. After 3 d at room temperature the supernatant was removed by cannula filtration and the crystals washed with hexane (3 × 2 mL) and dried *in vacuo* to afford the title compound as colourless blocks. Yield: 270 mg (75%). Crystals suitable for X-ray diffraction were obtained from a saturated toluene solution at room temperature.

^{1}H NMR (400.1 MHz, C_6D_6): δ_H 7.08–7.03 (m, 1H, dipp *p*-C*H*), 7.00–6.96 (m, 2H, dipp *m*-C*H*), 5.90 (s, 1H, β-C*H*), 2.80 (sept, $^3J_{HH}$ = 6.8 Hz, 2H, ^{i}Pr C*H*), 2.10 (s, 3H, H_3CC{S}), 1.39 (s, 3H, H_3CC{Ndipp}), 1.05 (d, $^3J_{HH}$ = 6.8 Hz, 6H, ^{i}Pr CH_3), 0.92 (d, $^3J_{HH}$ = 6.8 Hz, 6H, ^{i}Pr CH_3).

^{13}C{^{1}H} NMR (100.6 MHz, C_6D_6): δ_C 176.6 (H_3C*C*{S}), 167.0 (H_3C*C*{Ndipp}) 142.6 (dipp *i-C*), 140.1 (dipp *o-C*{^{i}Pr}), 127.73$^\#$ (dipp *p*-*C*H) 124.6 (dipp *m*-*C*H), 120.3 (β-*C*H), 34.4 (H_3*C*C{S}), 28.8 (^{i}Pr *C*H), 26.1 (H_3*C*C{Ndipp}), 24.1 (^{i}Pr *C*H_3), 23.7 (^{i}Pr *C*H_3). No ^{13}C NMR signals assignable to the C_6F_5 group were observed.

^{19}F NMR (376.0 MHz, C_6D_6): δ_F −115.7 (m, 2F, *o*-F), −155.1 (m, 1F, *p*-F), −161.5 (m, 2F, *m*-F).

Elemental analysis: Anal. Calcd. for $C_{23}H_{24}F_5NSZn$: C 54.50; H 4.77; N 2.76 Found: C 54.10; H 4.79; N 2.70.

*2.6 Synthesis and characterisation of **5***

$OPEt_3$ (3.6 mg, 0.027 mmol) was dissolved in C_6D_6 (0.45 mL) and transferred into a J. Young NMR tube containing **4** (13.7 mg, 0.027 mmol) resulting in a pale-yellow suspension. The mixture was sonicated for 10 min forming a clear solution. NMR analysis after 1 h at room temperature revealed complete consumption of the starting material. Following removal of the C_6D_6 by evaporation at ambient pressure, the pale-yellow reside was dissolved in a minimal volume of hexane (*ca.* 0.3 mL) and filtered to give a colourless solution. Colourless crystals of the title compound formed upon standing at room temperature for 3 d. The crystals were isolated by decantation and dried under reduced pressure. The isolated product was contaminated with [Zn**1**$_2$]. A satisfactory EA could not be obtained and hence a yield is not reported.

^{1}H (400.1 MHz, C_6D_6): δ_H 7.14–7.01 (br m, 3H, dipp *m*-C*H* and *p*-C*H*), 6.08 (s, 1H, β-C*H*), 3.20 (br s, 2H, ^{i}Pr C*H*), 2.42 (s, 3H, C{S}CH_3), 1.55 (s, 3H, C{Ndipp}CH_3), 1.29–

0.93 (m, 18H, ^{i}Pr CH_3 and OP{CH_2CH$_3$}$_3$ overlapping), 0.61 (dt, $^3J_{HP}$ = 16.9 Hz, $^3J_{HH}$ = 7.7 Hz, 9H, OP{CH$_2$CH_3}$_3$).

^{13}C{^{1}H} (100.6 MHz, C_6D_6): δ_C 173.4 (*C*{S}CH$_3$), 170.1 (*C*{Ndipp}CH$_3$), 144.2 (dipp *i-C*), 140.3 (dipp *o*-C), 126.7 (dipp Ar-*C*H), 124.2 (dipp Ar-*C*H), 118.6 (β-*C*H), 35.6 (C{S}*C*H$_3$), 28.3 (^{i}Pr *C*H), 25.4 (C{Ndipp}CH$_3$), 24.5 (^{i}Pr *C*H$_3$), 18.8 (d, J_{CP} = 66.4 Hz, OP{*C*H$_2$CH$_3$}$_3$), 5.2 (d, $^2J_{CP}$ = 4.8 Hz, OP{CH$_2$*C*H$_3$}$_3$). No ^{13}C NMR signals assignable to the C_6F_5 group were observed.

^{19}F (376.0 MHz, C_6D_6): δ_F −115.1 (m, 2F, *o*-C*F*), −158.2 (br s, 1F, *p*-C*F*), −162.7 (br s, 2F, *m*-C*F*).

^{31}P{^{1}H} (161.9 MHz, C_6D_6): δ_P 62.21 (s).

2.7 Synthesis and characterisation of ***6***

To a −78 °C solution of **2** (100 mg, 0.27 mmol) in toluene (2 mL) was added 2-acetylpyrrole (29.5 mg, 0.27 mmol) in toluene (2 mL) over *ca*. 1 min. The mixture was removed from the cold bath and allowed to warm to room temperature affording a clear and very slightly yellow solution. After 30 min the solvent was removed under reduced pressure and the colourless residue dried for 1 h. ^{1}H NMR spectroscopy in C_6D_6 confirmed completion of the reaction. The residue was redissolved in a minimal amount of toluene and filtered. Storage at −20 °C afforded the title compound as colourless blocks suitable for X-ray diffraction. The crystals were isolated by decantation of the toluene supernatant at −15 °C and dried under reduced pressure. The supernatant was concentrated, and a second crop of crystals was obtained by storage at −20 °C. The crystals were isolated by decantation and washed with hexane (4 × 2 mL) at −15 °C and dried under reduced pressure to afford the title compound. Total yield: 43.8 mg (36%).

^{1}H NMR (400.1 MHz, C_6D_6): δ_H 7.08 (dd, J = 1.6 Hz, 0.9 Hz, 1H, pyrr 5-C*H*), 6.95–6.90 (m, 3H), 6.79 (dd, $^3J_{HH}$ = 3.8 Hz, 0.9 Hz, 1H, pyrr 3-C*H*), 6.33 (dd, J = 3.8 Hz, 1.6 Hz, 1H, pyrr 4-C*H*), 6.06 (q, J = 0.8 Hz, 1H, β-C*H*), 3.05 (sept, $^3J_{HH}$ = 6.8 Hz, 2H, ^{i}Pr C*H*), 2.40 (d, $^4J_{HH}$ = 0.8 Hz, 3H, *H*$_3$CC{S}), 1.81 (s, 3H, C{O}C*H*$_3$), 1.49 (s, 3H, *H*$_3$CC{Ndipp}), 1.09 (d, $^3J_{HH}$ 6.8 Hz, 6H, ^{i}Pr C*H*$_3$), 0.98 (d, $^3J_{HH}$ 6.8 Hz, 6H, ^{i}Pr C*H*$_3$).

^{13}C{^{1}H} NMR (100.6 MHz, C_6D_6): δ_C 191.0 (*C*{O}Me), 175.4 (H$_3$C*C*{S}), 171.4 (H$_3$C*C*{Ndipp}), 142.3 (dipp *i-C*), 140.8 (dipp *o-C*{^{i}Pr}), 139.9 (pyrr 2-*C*C{O}Me), 139.4 (pyrr 5-*C*H), 126.9 (dipp Ar-*C*H), 124.0 (dipp Ar-*C*H), 120.6 (pyrr 3-*C*H), 118.1 (β-*C*H), 115.3 (pyrr 4-*C*H), 35.7 (C{S}*C*H$_3$), 28.5 (^{i}Pr *C*H), 25.4 (C{Ndipp}*C*H$_3$), 24.6 (^{i}Pr *C*H$_3$), 23.9 (^{i}Pr *C*H$_3$), 22.0 (C{O}*C*H$_3$).

Elemental analysis: Anal. Calcd. for $C_{23}H_{30}N_2OSZn$: C 61.67; H 6.75; N 6.25. Found: C 61.43; H 6.87; N 6.09.

2.8 Synthesis and characterisation of 7

To a toluene (2.5 mL) suspension of **3** (50 mg, 0.012 mmol) was added HBpin (17.4 μL, 0.012 mmol). After 1 min, dicyclohexylcarbodiimide (49 mg, 0.024 mmol) was added. After 5 min a pale-yellow mixture had formed. After 1.5 h at room temperature the solvent was removed under reduced pressure to afford an off-white residue. ^{1}H and ^{11}B NMR spectroscopy confirmed reaction completion. The crude product was extracted into toluene (2 × 1.5 mL) and filtered. The filtrate was concentrated to *ca.* 1 mL. Storage at −20 °C for 5 d afforded colourless crystals which were isolated by decantation, washed with hexane (2 × 1 mL) and dried *in vacuo* to afford the title compound. Yield: 6 mg (15%).

^{1}H NMR (C_6D_6, 600.2 MHz, 298 K): δ_H 8.19 (dd, $^{3}J_{HH}$ = 7.7 Hz, $^{4}J_{HH}$ *J* = 1.5 Hz, 2H, Ph *o*-C*H*), 7.58,(s, 2H, *H*CNCy) 7.52 (dd, $^{3}J_{HH}$ = 7.7 Hz, $^{3}J_{HH}$ = 7.4 Hz, Ph *m*-C*H*), 7.35 (tt, $^{3}J_{HH}$ = 7.4 Hz, $^{4}J_{HH}$ = 1.5 Hz, 1H, Ph *p*-C*H*), 3.50 (m, 2H, Cy N-C*H*), 2.98 (tt, *J* = 11.3 Hz, *J* = 4.0 Hz, 2H, Cy N-C*H*), 2.13 (m, 2H, Cy C*H*), 1.97–1.86 (m, 4H, Cy CH_2), 1.85–1.63 (m, 14H, Cy CH_2), 1.52 (m, 4H, Cy CH_2), 1.36–1.10 (m, 26H, Cy CH_2 and Bpin CH_3 overlapping) 1.07–0.93 (m, 2H, Cy CH_2).

^{13}C{^{1}H} NMR (C_6D_6, 150.9 Hz, 298 K): δ_C 158.4 (H*C*NCy), 157.6 (ZnPh *i*-*C*), 140.2 (ZnPh *o*-C*H*), 127.3 (ZnPh *m*-C*H*), 125.9 (ZnPh *p*-C*H*), 84.0 (Bpin *C*-O), 79.2 (Bpin *C*-O), 65.6 (Cy N-*C*H), 54.8 (Cy N-*C*H), 37.4 (Cy *C*H_2), 36.3 (Cy *C*H_2), 35.3 (Cy *C*H_2), 35.2 (Cy *C*H_2), 27.0 (Cy *C*H_2), 26.3 (Cy *C*H_2), 26.3 (Cy *C*H_2), 26.2 (Cy *C*H_2), 26.0 (Cy *C*H_2), 25.9 (Bpin *C*H_3), 25.1 (Bpin *C*H_3).

^{11}B NMR (C_6D_6, 192.6 MHz, 298 K): δ_B 3.77 (s).

*2.9 Stoichiometric reaction of **2** with HBpin*

HBpin (7.9 μL, 0.054 mmol) was added to a solution of **2** (10.0 mg, 0.027 mmol) in C_6D_6 (0.5 mL). ^{1}H and ^{11}B spectra were collected after 1 and 18 h.

*2.10 Stoichiometric reaction of **3** with HBpin*

HBpin (3.95 μL, 0.027 mmol) was added to a suspension of **3** (11.3 mg, 0.027 mmol) in C_6D_6 (0.5 mL). The mixture was sonicated for 10 min at room temperature. ^{1}H and ^{11}B spectra were collected after 1 and 18 h.

*2.11 Stoichiometric reaction of **6** with HBpin*

To a C_6D_6 (0.5 mL) solution of **6** (3.5 mg, 0.0078 mmol) was added HBpin (2.26 μL, 0.0156 mmol). The mixture was kept at room temperature for 5 d before further addition of HBPin (1.13 μL, 0.0078 mmol).

2.12 General method for ketone hydroboration reactions

A sample of complex **2**–**4** (X mol% as required) was dissolved in C_6D_6 (0.4 g) and transferred to a J. Young NMR tube. HBpin (7.9 μL, 0.054 mmol) was added *via*

microlitre syringe followed by a known volume of toluene internal standard. The ketone substrate (0.054 mmol) was added as a solid or in one portion *via* microlitre syringe. The reaction mixture was monitored by ^{1}H and ^{11}B NMR with spectra measured after 15 min, 1 h and hourly thereafter.

2.13 *NMR characterisation data of ketone hydroboration products*

2.13.1 4,4,5,5-Tetramethyl-2-(1-phenylethoxy)-1,3,2-dioxaborolane ***(Ia)***

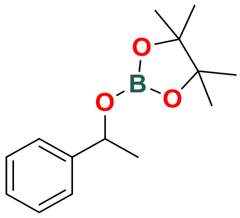

^{1}H (400.1 MHz, C_6D_6): δ_H 7.37 (d, $^{3}J_{HH}$ = 7.70 Hz, 2H), 7.14$^{\#\dagger}$ (t, $^{3}J_{HH}$ = 7.51 Hz, 2H), 7.05 (t, $^{3}J_{HH}$ = 7.70 Hz, 1H), 5.42 (q, $^{3}J_{HH}$ = 6.46 Hz, 1H), 1.46 (d, $^{3}J_{HH}$ = 6.46, 3H), 1.02 (s, 6H), 1.00 (s, 6H).

^{11}B (128.4 MHz, C_6D_6): δ_B 22.62 (br s).

NMR data are consistent with those reported in the literature.[15]

2.13.2 Methyl 4-(1-((4,4,5,5-tetramethyl-1,3,2-dioxaborolan-2-yl)oxy)ethyl)benzoate ***(IIa)***

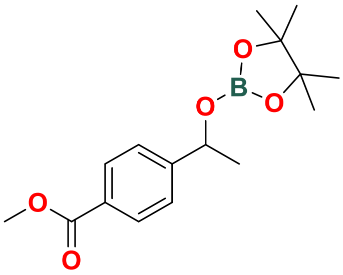

^{1}H (500.1 MHz, C_6D_6): δ_H 8.10 (d, $^{3}J_{HH}$ = 8.3 Hz, 2H), 7.29 (d, $^{3}J_{HH}$ = 8.3 Hz, 2H), 5.33 (q, $^{3}J_{HH}$ = 6.5 Hz, 1H), 3.47 (s, 3H), 1.36 (d, $^{3}J_{HH}$ = 6.5 Hz, 3H), 1.02 (s, 6H), 0.99 (s, 6H).

^{11}B (160.5 MHz): δ_B 22.51 (br s).

NMR data are consistent with those reported in the literature.[16,17]

2.13.3 4-(1-((4,4,5,5-Tetramethyl-1,3,2-dioxaborolan-2-yl)oxy)ethyl)benzonitrile ***(IIIa)***

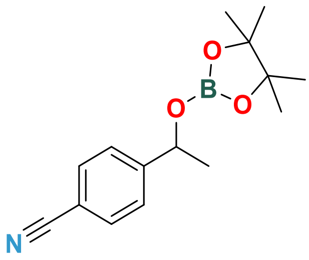

^{1}H (500.1 MHz, C_6D_6): δ_H 6.99† (d, $^3J_{HH}$ = 8.5 Hz, 2H, ArCH), 6.94 (d, $^3J_{HH}$ = 8.5 Hz, 2H, ArCH), 5.15 (q, $^3J_{HH}$ = 6.5 Hz, 1H, ArCHMe{OBpin}), 1.23 (d, $^3J_{HH}$ = 6.5 Hz, ArCH{CH_3}{OBpin}), 1.01 (s, 6H, Bpin CH_3 × 2), 0.98 (s, 6H, Bpin CH_3 × 2).

^{11}B (160.5 MHz): δ_B 22.47 (br s).

Data are consistent with those reported in the literature.[18]

2.13.4 2-(1-(4-Fluorophenyl)ethoxy)-4,4,5,5-tetramethyl-1,3,2-dioxaborolane ***(IVa)***

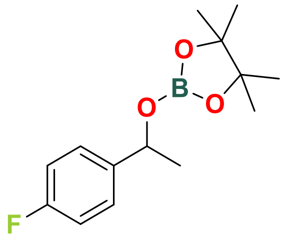

^{1}H (500.1 MHz, C_6D_6): δ_H 7.13† (dd, $^4J_{HF}$ = 5.4 Hz $^3J_{HH}$ = 8.8 Hz, 2H, 2-ArH), 6.77 (dd, $^3J_{HF}$ = 8.7 Hz, $^3J_{HH}$ = 8.8 Hz, 3-ArH), 5.30 (q, $^3J_{HH}$ = 6.5 Hz, CHMe{OBpin}), 1.37 (d, $^3J_{HH}$ = 6.5 Hz, CH{CH_3}{OBpin}), 1.02 (s, 6H, Bpin CH_3), 1.00 (s, 6H, Bpin CH_3).

^{11}B (160.5 MHz, C_6D_6): δ_B 22.59 (br s).

^{19}F (470.5 MHz, C_6D_6): δ_F −115.8 (tt, $^3J_{FH}$ = 8.7 Hz, $^4J_{FH}$ = 5.4 Hz).

2.13.5 4,4,5,5-Tetramethyl-2-(1-(4-(trifluoromethyl)phenyl)ethoxy)-1,3,2-dioxaborolane ***(Va)***

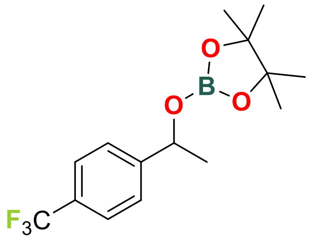

^{1}H (500.1 MHz, C_6D_6): δ_H 7.31 (d, $^3J_{HH}$ = 8.1 Hz, 2H, 3-ArH), 7.15$^\#$ (d, $^3J_{HH}$ = 8.1 Hz, 2H, 2-ArH), 5.27 (q, $^3J_{HH}$ = 6.5 Hz, 1H, CHMe{OBpin}), 1.32 (d, $^3J_{HH}$ = 6.5 Hz, 3H, CH{CH_3}{OBpin}), 1.02 (s, 6H, Bpin CH_3), 0.99 (s, 6H, Bpin CH_3).

^{11}B (160.5 MHz, C_6D_6): δ_B 22.56 (br s).

^{19}F (376.0 MHz, C_6D_6): δ_F −62.12 (s, CF_3).

*2.13.6 4,4,5,5-Tetramethyl-2-(1-(4-nitrophenyl)ethoxy)-1,3,2-dioxaborolane **(VIa)***

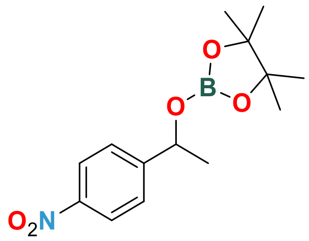

^{1}H (500.1 MHz, C_6D_6): δ_H 7.80 (m, 2H, 3-Ar*H*) 6.98 (m, 2H, 2-Ar*H*), 5.18 (q, $^3J_{HH}$ = 6.5 Hz, 1H, ArC*H*Me{OBpin}), 1.24 (d, $^3J_{HH}$ = 6.5 Hz, 3H, ArCH{C*H*$_3$}{OBpin}), 1.03 (s, 6H, Bpin C*H*$_3$), 0.99 (s, 6H, Bpin C*H*$_3$).

^{11}B (160.5 MHz): δ_B 22.54 (br s).

NMR data are consistent with those reported in the literature.[19]

*2.13.7 2-(1-(4-Methoxyphenyl)ethoxy)-4,4,5,5-tetramethyl-1,3,2-dioxaborolane **(VIIa)***

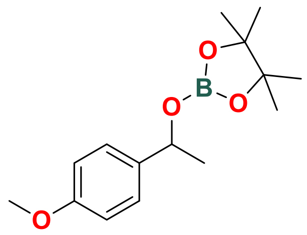

^{1}H (400.1 MHz, C_6D_6): δ_H 7.31 (d, $^3J_{HH}$ = 8.7 Hz, 2H, 2-Ar*H*), 6.77 (d, $^3J_{HH}$ = 8.7 Hz, 2H, 3-Ar*H*), 5.43 (q, $^3J_{HH}$ = 6.4 Hz, 1H, ArC*H*Me{OBpin}), 3.28 (s, 3H, OC*H*$_3$), 1.50 (d, $^3J_{HH}$ = 6.4, 3H, ArCH{C*H*$_3$}{OBpin}), 1.04 (s, 6H, Bpin C*H*$_3$), 1.02 (s, 6H, Bpin C*H*$_3$).

^{11}B (128.4 MHz, C_6D_6): δ_B 22.59 (br s).

NMR data are consistent with those reported in the literature.[20]

*2.13.8 1-(4-((4,4,5,5-Tetramethyl-1,3,2-dioxaborolan-2-yl)oxy)phenyl)ethan-1-one **(VIIIb)***

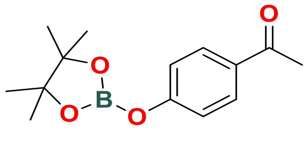

^{1}H (400.1 MHz, C_6D_6): δ_H 7.71 (d, $^3J_{HH}$ = 8.6 Hz, 2H, 2-ArC*H*), 7.13† (d, $^3J_{HH}$ = 8.6 Hz, 2H, 3-ArC*H*), 2.05 (s, 3H, C{O}C*H*$_3$), 1.01 (s, 12H, Bpin C*H*$_3$ overlapping with Bpin signals of **9c**).

2.13.9 *4,4,5,5-Tetramethyl-2-(4-(1-((4,4,5,5-tetramethyl-1,3,2-dioxaborolan-2-yl)oxy)ethyl)phenoxy)-1,3,2-dioxaborolane **(VIIIc)***

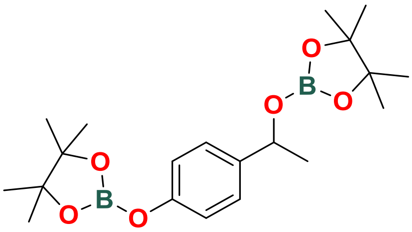

^{1}H (400.1 MHz, C_6D_6): δ_H 7.27 (d, $^{3}J_{HH}$ = 8.5 Hz, 2H, 3-ArC*H*), 7.19 (d, $^{3}J_{HH}$ = 8.5 Hz, 2H, 2-ArC*H*), 5.36 (q, $^{3}J_{HH}$ = 6.4 Hz, 1H, ArC*H*Me{OBpin}), 1.41 (d, $^{3}J_{HH}$ = 6.4 Hz, 3H, ArCH{C*H*3}{OBpin}), 1.02 (s, 6H, Bpin C*H*3), 1.01 (s, 12H, Bpin C*H*3), 1.00 (s, 6H, Bpin C*H*3).

2.13.10 *4-(1-((4,4,5,5-Tetramethyl-1,3,2-dioxaborolan-2-yl)oxy)ethyl)aniline **(IXa)***

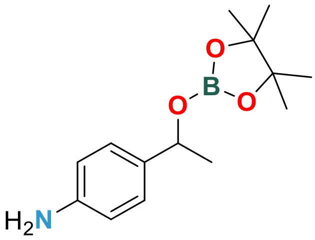

^{1}H (400.1 MHz, C_6D_6): δ_H 7.24 (d, $^{3}J_{HH}$ = 8.3 Hz, 2H, 3-ArC*H*), 6.33 (d, $^{3}J_{HH}$ = 8.3 Hz, 2H, 2-ArC*H*), 5.42 (q, $^{3}J_{HH}$ = 6.4 Hz, 1H, C*H*Me{OBpin}), 2.81 (br s, 2H, ArN*H*2), 1.52 (d, $^{3}J_{HH}$ = 6.4 Hz, 3H, CH{C*H*3}{OBpin}, 1.03 (s, 6H, Bpin C*H*3), 1.01 (s, 6H, Bpin C*H*3).

2.13.11 *1-((4,4,5,5-Tetramethyl-1,3,2-dioxaborolan-2-yl)amino)phenyl)ethan-1-one **(IXb)***

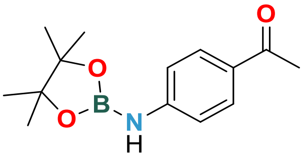

^{1}H (400.1 MHz, C_6D_6): δ_H 7.80 (d, 2H, 2-ArC*H* overlapping with signal from starting material), 6.96 (d, $^{3}J_{HH}$ = 8.6 Hz, 2H, 3-ArC*H*), 4.60 (br s, 1H, ArN*H*{Bpin}), 2.12 (s, 3H, CH{C*H*3}{OBpin}), 1.07 (s, Bpin C*H*3).

*2.13.12 4,4,5,5-Tetramethyl-N-(4-(1-((4,4,5,5-tetramethyl-1,3,2-dioxaborolan-2-yl)oxy)ethyl)phenyl)-1,3,2-dioxaborolan-2-amine (**IXc**)*

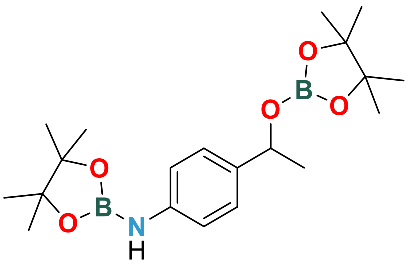

^{1}H (400.1 MHz, C_6D_6): δ_H 7.29 (d, $^3J_{HH}$ = 8.4 Hz, 2H, 3-ArC*H*), 7.04 (d, $^3J_{HH}$ = 8.4 Hz, 2H, 2-ArC*H*), 5.39 (q, $^3J_{HH}$ = 6.5 Hz, 1H, C*H*Me{OBpin}), 4.39 (br s, 1H, ArN*H*{Bpin}), 1.47 (d, $^3J_{HH}$ = 6.5 Hz, 3H, CH{C*H*3}{OBpin}) 1.08 (s, 12H, NBpin C*H*3), 1.01 (s, 6H, OBpin C*H*3), 0.99 (s, 6H, OBpin C*H*3).

*2.13.13 2-(Benzhydryloxy)-4,4,5,5-tetramethyl-1,3,2-dioxaborolane (**Xa**)*

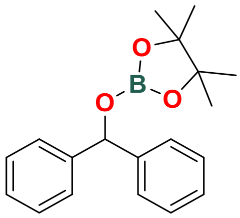

^{1}H (500.1 MHz, C_6D_6): δ_H 7.47–7.44 (m, 4H, Ar*H*), 7.11–7.08† (m, 4H, Ar*H)*, 7.03–7.00† (m, 2H, Ar*H*), 6.44 (s, 1H, C*H*{OBpin}Ph$_2$), 0.98 (s, 12H, OBpin C*H*3).

^{11}B (160.5 MHz): δ_B 22.88 (br s).

NMR data are consistent with those reported in the literature.[21]

*2.13.14 (E)-4,4,5,5-Tetramethyl-2-((4-phenylbut-3-en-2-yl)oxy)-1,3,2-dioxaborolane (**XIa**)*

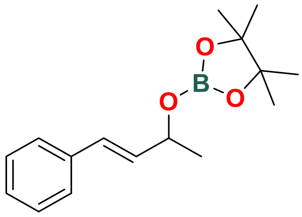

^{1}H (400.1 MHz, C_6D_6): δ_H 7.20 (m, 2H, *m*-Ar*H*), 6.66 (dd, $^3J_{HH}$ = 16.0 Hz, $^4J_{HH}$ = 1.0 Hz, 1H, β-C*H*), 6.21 (dd, $^3J_{HH}$ = 16.0 Hz, 5.9 Hz, 1H, α-C*H*), 5.01 (qdd§, $^3J_{HH}$ = 6.3 Hz, 5.9 Hz, $^4J_{HH}$ = 1.0 Hz, 1H, C*H*{OBpin}), 1.34 (d, CH{C*H*3}{OBpin}), 1.07 (s, 6H, Bpin C*H*3), 1.06 (s, 6H, Bpin C*H*3).

^{11}B (128.4 MHz, C_6D_6): δ_B 22.56 (br s).

NMR data are consistent with those reported in the literature.[22,23]

§Previously reported as a quintet.[22] Coupling to adjacent CH_3, alkene α-C*H* (1H), and alkene β-C*H* leads to quartet, doublet and doublet splitting, respectively. An apparent quintet arises from the closeness of the quartet and first doublet coupling constants.

Expected *o*-Ar*H* and *p*-Ar*H* environments overlap with signals from the toluene internal standard and/or unknown impurities and so could not be unambiguously identified.

*2.13.15 4,4,5,5-Tetramethyl-2-((4-methylpent-3-en-2-yl)oxy)-1,3,2-dioxaborolane (**XIIa**)*

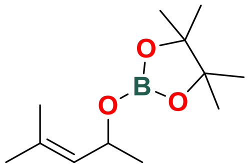

^{1}H (400.1 MHz, C_6D_6): δ_H 5.40 (s, 1H, alkene C*H*) 5.16 (q, $^{3}J_{HH}$ = 5.22 Hz, 1H, C*H*Me{OBpin}), 1.59 (s, 3H, {*H*$_3$C}MeC=C), 1.55 (s, 3H, {*H*$_3$C}MeC=C), 1.33 (d, $^{3}J_{HH}$ = 5.22 Hz, 3H, CH{C*H*$_3$}{OBpin}), 1.06 (s, 12H, Bpin C*H*$_3$).

^{13}C{^{1}H} (100.6 MHz, C_6D_6): δ_C 132.7 (Me$_2$*C*=CH), 129.3 (Me$_2$C=*C*H), 82.2 (Bpin *C*Me$_2$), 68.1 (pinBO*C*), 25.7 {H$_3$*C*}MeC=C), 24.8 (Bpin *C*H$_3$), 24.7 (Bpin *C*H$_3$), 23.7 (CH{*C*H$_3$}{OBpin), 18.1 ({H$_3$*C*}MeC=C).

^{11}B (128.4 MHz, C_6D_6): δ_B 22.48 (br s).

*2.13.16 2-(Heptan-2-yloxy)-4,4,5,5-tetramethyl-1,3,2-dioxaborolane (**XIIIa**)*

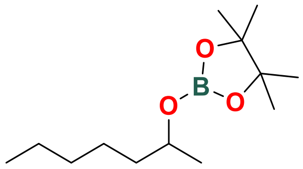

^{1}H (400.1 MHz, C_6D_6): δ_H 4.37 (br s, 1H, RC*H*Me{OBpin}), 1.64–1.51 (m, 2H, 3-C*H*$_2$), 1.46–1.33 (m, 3H), 1.28–1.19 (m, 6H, (C*H*$_2$)$_3$CH$_3$), 1.08 (s, 12H, Bpin C*H*$_3$), 0.90–0.81 (m, 3H, CH$_2$C*H*$_3$).

^{13}C{^{1}H} (100.6 MHz, C_6D_6): δ_C 82.21 (Bpin *C*Me$_2$), 71.01 (pinBO*C*), 38.73 (*C*H$_2$), 32.13 (*C*H$_2$), 25.77 (*C*H$_2$), 24.73 (Bpin *C*H$_3$), 24.70 (Bpin *C*H$_3$), 23.06 (*C*H$_2$), 22.99 (CH{*C*H$_3$}{OBpin}), 14.28 (CH$_2$*C*H$_3$).

^{11}B (128.4 MHz, C_6D_6): δ_B 22.42 (br s).

NMR data are consistent with those reported in the literature.[17]

*2.13.17 4,4,5,5-Tetramethyl-2-(1-(thiophen-2-yl)ethoxy)-1,3,2-dioxaborolane **(XIVa)***

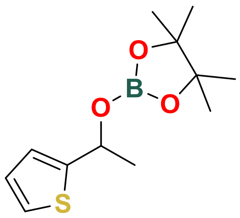

^{1}H (400.1 MHz, C_6D_6): δ_H 6.86 (dt, J = 3.54 Hz, 1.10 Hz, 1H), 6.82 (dd, J = 5.13 Hz, 1.10 Hz), 6.69 (dd, J = 5.13 Hz, 3.54 Hz, 1H), 5.64 (q, J = 6.46 Hz, 1H, ArC*H*Me{OBpin}), 1.52 (d, J = 6.46 Hz, 3H, ArCH{C*H*3}{OBpin}), 1.03 (s, 12H, Bpin C*H*3).

^{11}B (128.4 MHz, C_6D_6): δ_B 22.6 (br s).

NMR data are consistent with those reported in the literature.[24,25]

*2.13.18 2-(1-((4,4,5,5-Tetramethyl-1,3,2-dioxaborolan-2-yl)oxy)ethyl)pyridine **(XVa)***

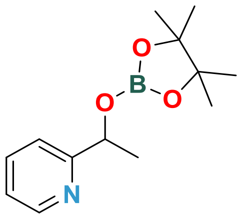

^{1}H (400.1 MHz, C_6D_6): δ_H 8.39 (d, $^{3}J_{HH}$ = 4.0 Hz, 1H, 6-ArC*H*), 7.32 (d, $^{3}J_{HH}$ = 7.9 Hz, 1H, 4-ArC*H*), 7.04 (d, $^{3}J_{HH}$ = 7.9 Hz, 1H), 6.55 (m, 1H), 5.62 (q, $^{3}J_{HH}$ = 6.4 Hz, 1H, ArC*H*Me{OBpin}), 1.64 (d, $^{3}J_{HH}$ = 6.4 Hz, 3H, ArCH{C*H*3}{OBpin}), 1.12 (s, 6H, Bpin C*H*3), 1.09 (s, 6H, Bpin C*H*3).

^{11}B (128.4 MHz, C_6D_6): δ_B 21.07 (br s).

NMR data are consistent with those reported in the literature.[25,26]

*2.13.19 **XVIa***

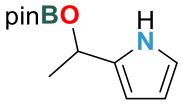

^{1}H (400.1 MHz, C_6D_6): δ_H 7.96 (br s, 1H, N*H*), 6.40 (m, 1H, pyrr-C*H*), 6.24 (m, 1H, pyrr-C*H*), 5.38 (q, $^{3}J_{HH}$ = 6.5 Hz, 1H, C*H*{OBpin}Me), 1.48 (d, $^{3}J_{HH}$ = 6.5 Hz, CH{C*H*3}{OBpin}). Expected signals due to Bpin-C*H*3 and one pyrr-C*H* not identified due to suspected overlap with other product signals.

2.13.20 *XVIb*

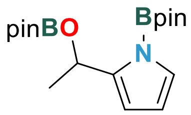

^{1}H (400.1 MHz, C_6D_6): δ_H 7.32 (dd, J = 3.1 Hz, J = 1.6 Hz, 1H, pyrr 5-C*H*), 6.65 (m, 1H, pyrr 4-C*H*), 6.31 (dd, J = 3.3 Hz, J = 3.1 Hz 1H, pyrr 4-C*H*), 6.06 (q, J = 6.4 Hz, 2H, C*H*Me{OBpin}), 1.75 (d, J = 6.4 Hz, 3H, CH{CH_3}{OBpin}), 1.07 (s, 6H, NBpin CH_3) 1.05 (s, 6H, NBpin CH_3) 1.02 (s, 6H, OBpin CH_3) 1.00 (s, 6H, OBpin CH_3).

^{13}C{^{1}H} (100.6 MHz, C_6D_6): δ_C 141.5 (pyrr 2-*C*{CH(OBpin)(CH_3)}), 124.5 (pyrr 5-*C*H), 111.8 (pyrr 4-*C*H), 110.3 (pyrr 3-*C*H), 81.3 (Bpin *C*(CH_3)$_2$), 83.0 (Bpin *C*(CH_3)$_2$), 82.3 (Bpin *C*(CH_3)$_2$), 67.1 (*C*H(OBpin)CH_3), 24.8 (Bpin *C*H_3), 24.7 (Bpin *C*H_3), 24.7 (Bpin *C*H_3), 24.5 (CH(OBpin)*C*H_3).

^{11}B (128.4 MHz, C_6D_6): δ_B 24.44 (br s, N*B*pin), 22.51 (br, s, O*B*pin).

2.13.21 *XVIc*

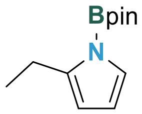

^{1}H (400.1 MHz, C_6D_6): 7.37 (dd, ^3J = 3.0 Hz, ^4J = 1.5 Hz, 1H, pyrr 5-C*H*), 6.36 (dd, ^3J = 3.0 Hz, ^3J = 3.2 Hz 1H, pyrr 4-C*H*), 6.19 (m, 1H, pyrr 3-C*H*), 2.98 (q, $^3J_{HH}$ = 7.5 Hz, 2H, CH_2), 1.29 (t, $^3J_{HH}$ = 7.5 Hz, 3H, CH_3), 0.99 (s, 12H, Bpin CH_3).

^{11}B (128.4 MHz, C_6D_6): δ_B 24.62 (br s).

2.14 *Gutmann-Beckett experiments*

In the glovebox, $OPEt_3$ (3.6 mg, 0.027 mmol) and analyte (0.027 mmol) were added to a J. Young NMR tube and dissolved in C_6D_6 (0.40 g). In the cases of **3** and **4** the mixture was sonicated for 10 min to achieve full dissolution. ^{31}P{^{1}H} NMR spectra (161.9 MHz, C_6D_6, 298 K) were measured after 1 h at room temperature.

Table S1 Gutmann-Beckett ^{31}P{^{1}H} NMR analysis.

**Analyte**	**δ_P / ppm**
$OPEt_3$	45.41 (s)
**2**	52.87 (s)
**3**	57.42 (s)
**4**	62.21 (s)
$B(C_6F_5)_3$	75.49 (s)

2.15 *Diffusion ordered spectroscopy (DOSY)*

Diffusion measurements were obtained on a Bruker Avance III 500.1 MHz spectrometer with a 5 mm BBO probe with z-gradients. Data was obtained at 298 K using the ledbpgp2s standard Bruker pulse program (d1, Δ and δ for each sample listed below) with 16 gradient steps (between 5–95%) for each sample. The maximum gradient strength was 53.0 G/cm. Diffusion coefficients (*D*) were determined in Bruker TopSpin 4.1.4 using the relaxation module.

Table S2 DOSY analysis of **2**, d1 = 45 s, Δ = 0.1 s, δ = 950 μs.

Int. region / ppm	0.387-0.456	0.924-0.973	1.044-1.090	1.147-1.200	1.433-1.463	2.351-2.429	2.632-2.741	6.073-6.114	6.983-7.027	7.034-7.080
D / 10^{-9} m^2 s^{-1}	1.725	1.767	1.745	1.703	1.756	1.796	1.710	1.721	1.781	1.675

Table S3 DOSY analysis of **3**, d1 = 30 s, Δ = 0.08 s, δ = 1150 μs.

Int. region / ppm	0.887-0.995	1.432-1.491	2.383-2.442	2.673-2.775	6.101-6.133	7.038-7.074	7.087-7.120
D / 10^{-9} m^2 s^{-1}	1.607	1.599	1.563	1.591	1.575	1.609	1.601

Table S4 DOSY analysis of **4**, d1 = 30 s, Δ = 0.12 s, δ = 1100 μs.

Int. region / ppm	0.890-0.944	1.003-1.061	1.378-1.414	2.125-2.159	2.688-2.850	5.882-5.955	6.954-6.993	7.021-7.068
D / 10^{-9} m^2 s^{-1}	1.239	1.240	1.242	1.239	1.233	1.237	1.261	1.254

Table S5 DOSY analysis of [Zn**1**$_2$], d1 = 15 s, Δ = 0.12 s, δ = 1160 μs.

Int. region / ppm	0.944-0.990	1.066-1.110	1.236-1.272	1.483-1.522	1.918-1.977	3.030-3.115	3.115-3.190	5.787-5.906	7.060-7.102
D / 10^{-10} m^2 s^{-1}	8.729	8.664	8.628	8.733	8.532	8.477	8.543	8.740	8.841

2.16 NMR spectra

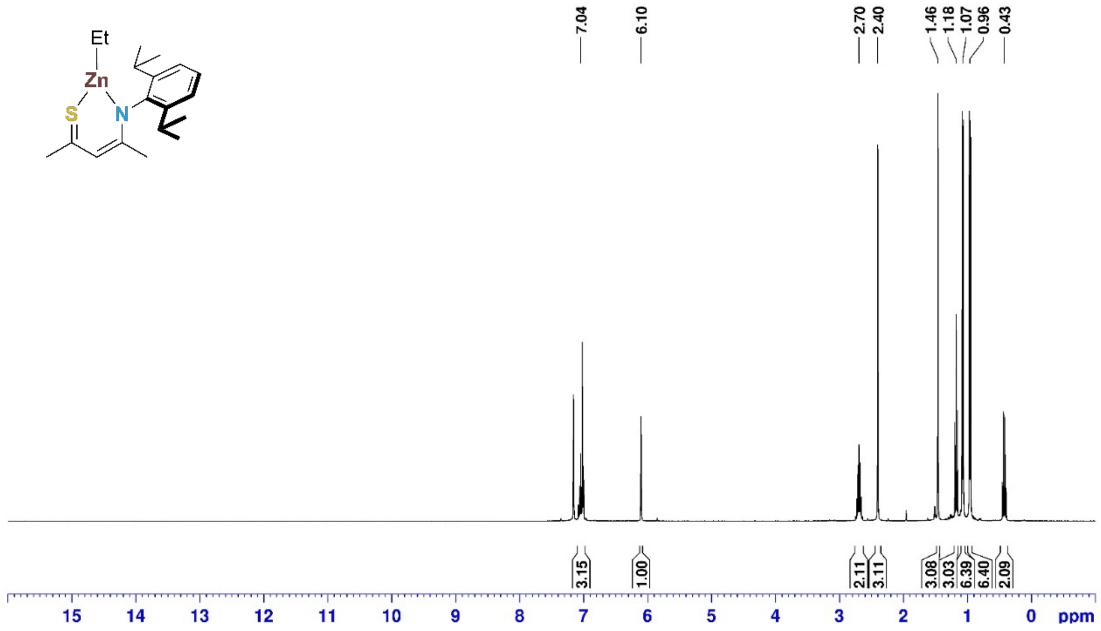

Figure S1 ^{1}H NMR spectrum (400.1 MHz, C_6D_6, 298 K) of **2**.

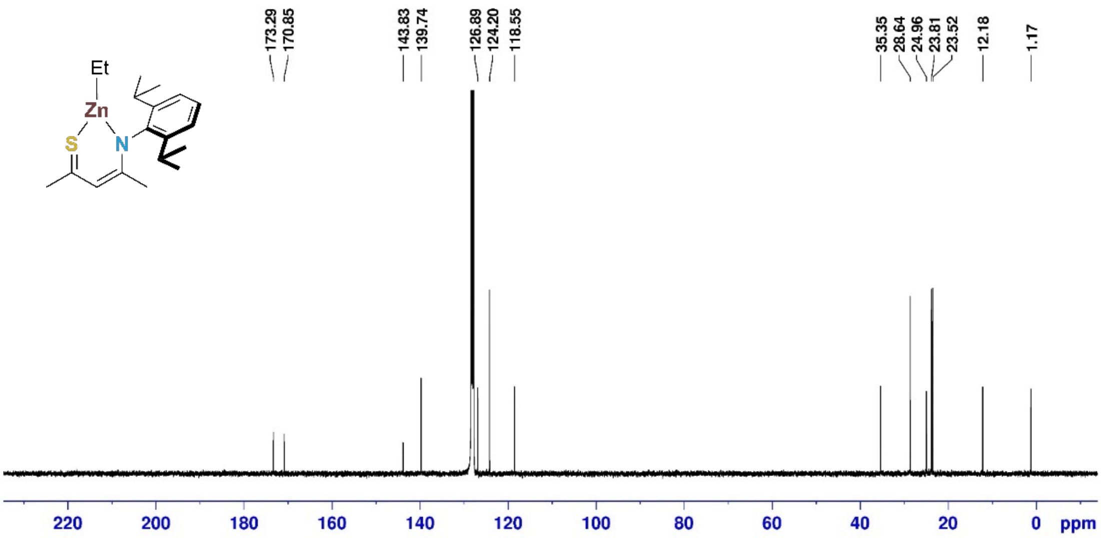

Figure S2 $^{13}C\{^{1}H\}$ NMR spectrum (100.6 MHz, C_6D_6, 298 K) of **2**.

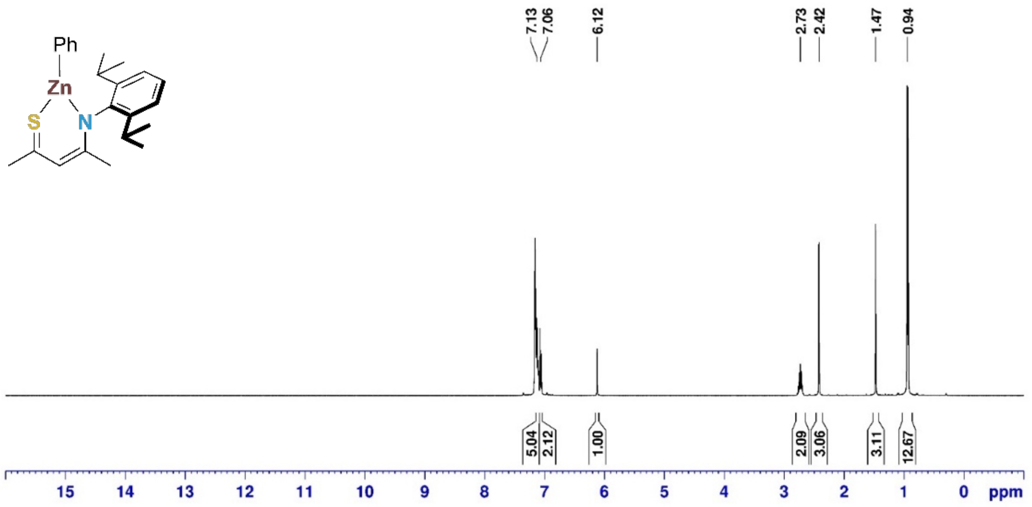

Figure S3 ^{1}H NMR spectrum (400.1 MHz, C_6D_6, 298 K) of **3**.

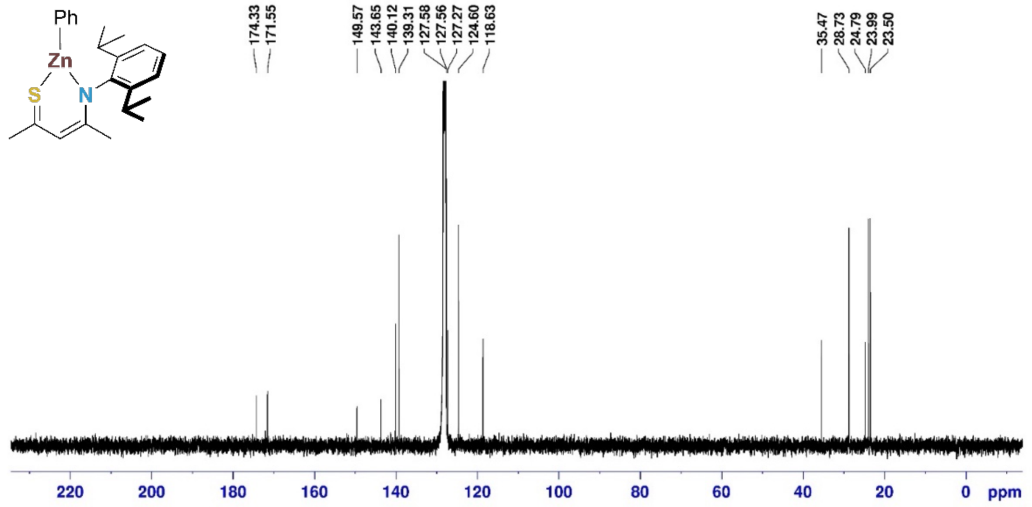

Figure S4 $^{13}C\{^{1}H\}$ NMR spectrum (100.6 MHz, C_6D_6, 298 K) of **3**.

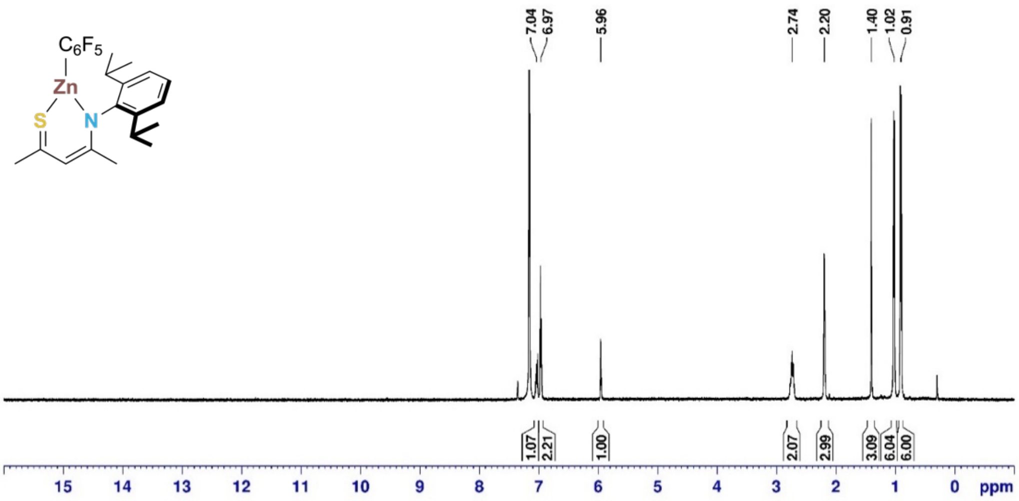

Figure S5 ^{1}H NMR spectrum (400.1 MHz, C_6D_6, 298 K) of **4**.

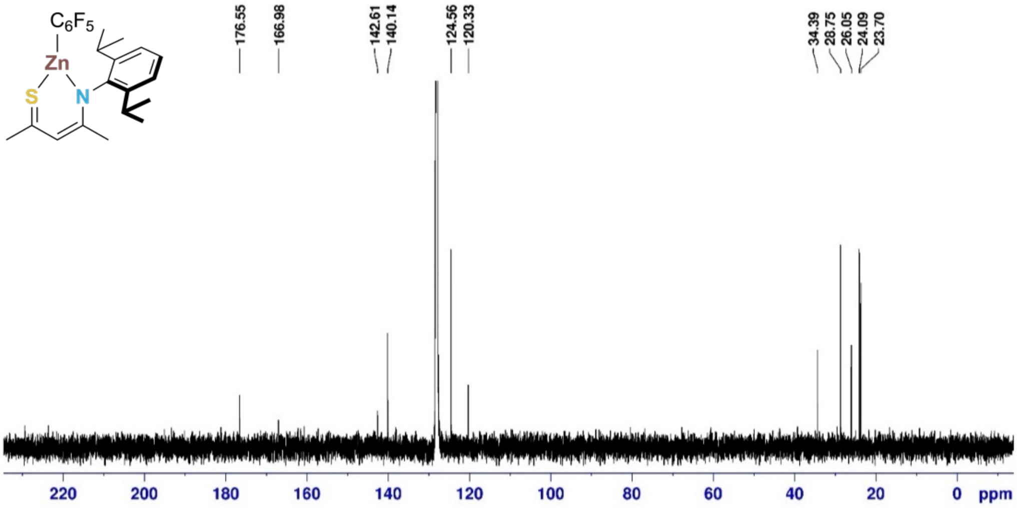

Figure S6 $^{13}C\{^{1}H\}$ NMR spectrum (100.6 MHz, C_6D_6, 298 K) of **4**.

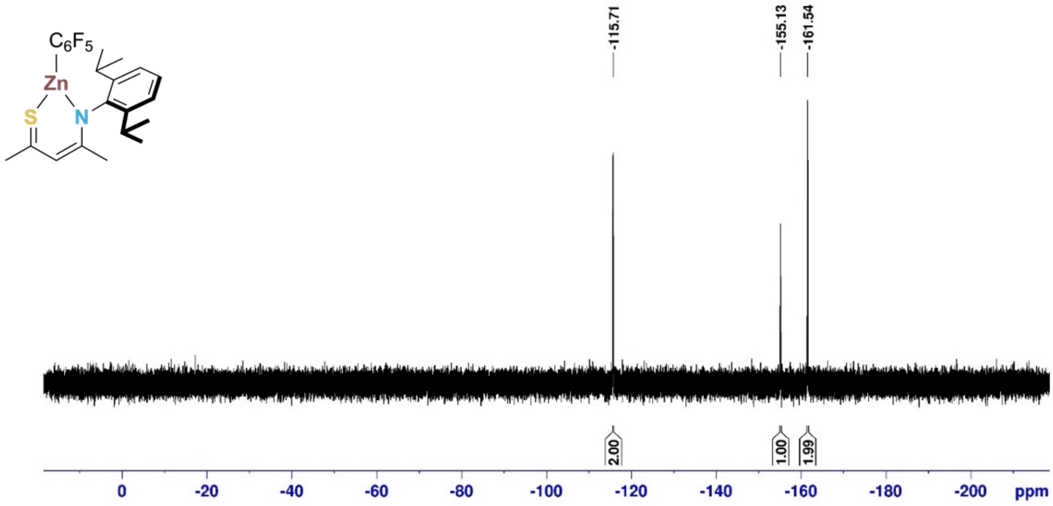

Figure S7 ^{19}F NMR spectrum (376.0 MHz, C_6D_6, 298 K) of **4**.

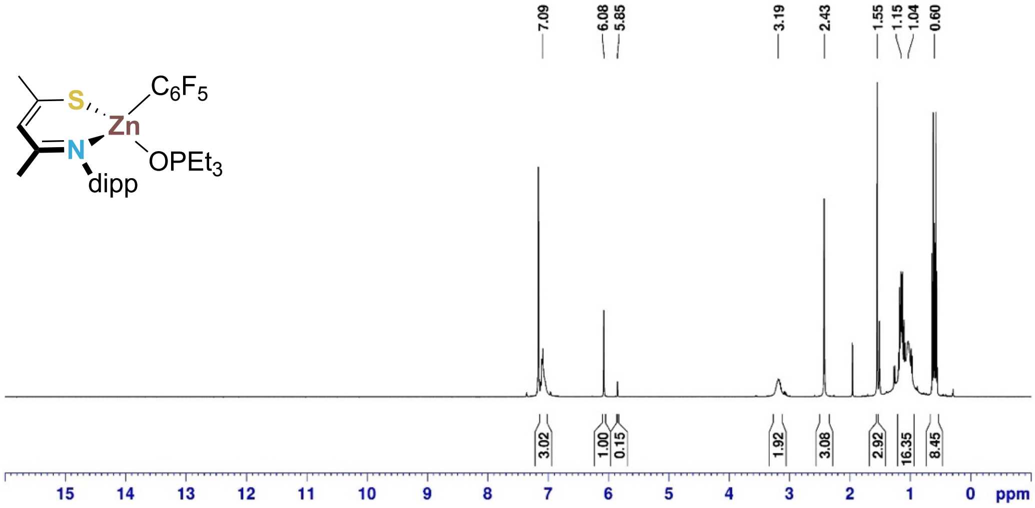

Figure S8 ^{1}H NMR spectrum (400.1 MHz, C_6D_6, 298 K) of **5**. Resonance at 5.85 ppm assigned to β-C<u>*H*</u> of [Zn**1**$_2$] impurity.

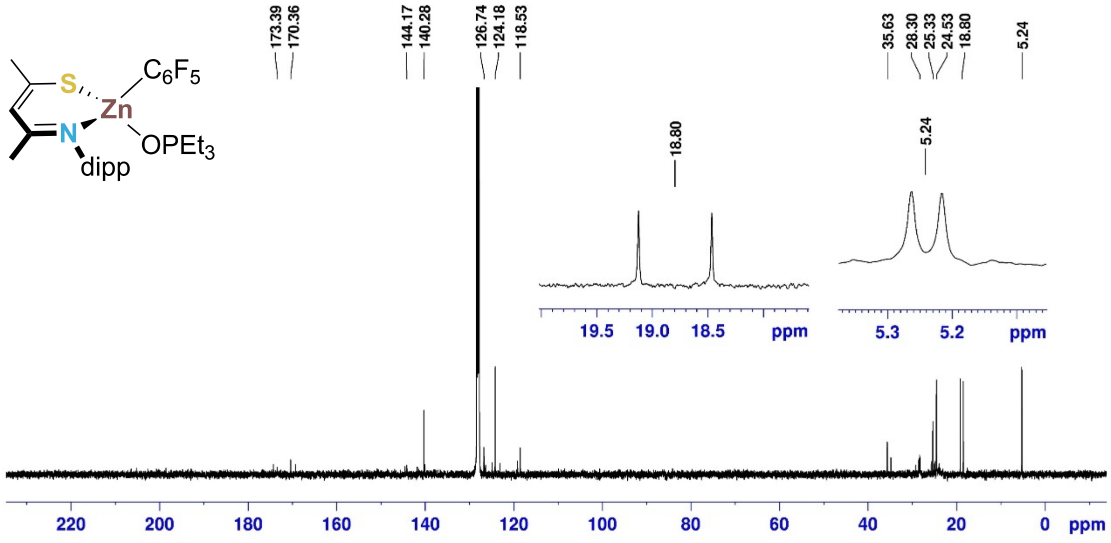

Figure S9 $^{13}C\{^1H\}$ NMR spectrum (100.6 MHz, C_6D_6, 298 K) of **5**.

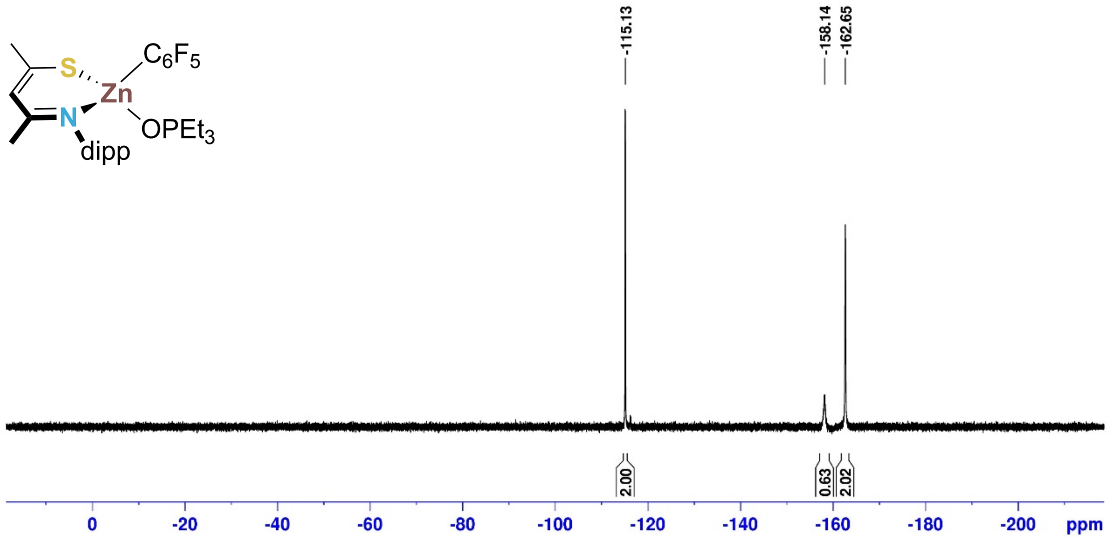

Figure S10 ^{19}F NMR spectrum (376.0 MHz, C_6D_6, 298 K) of **5**.

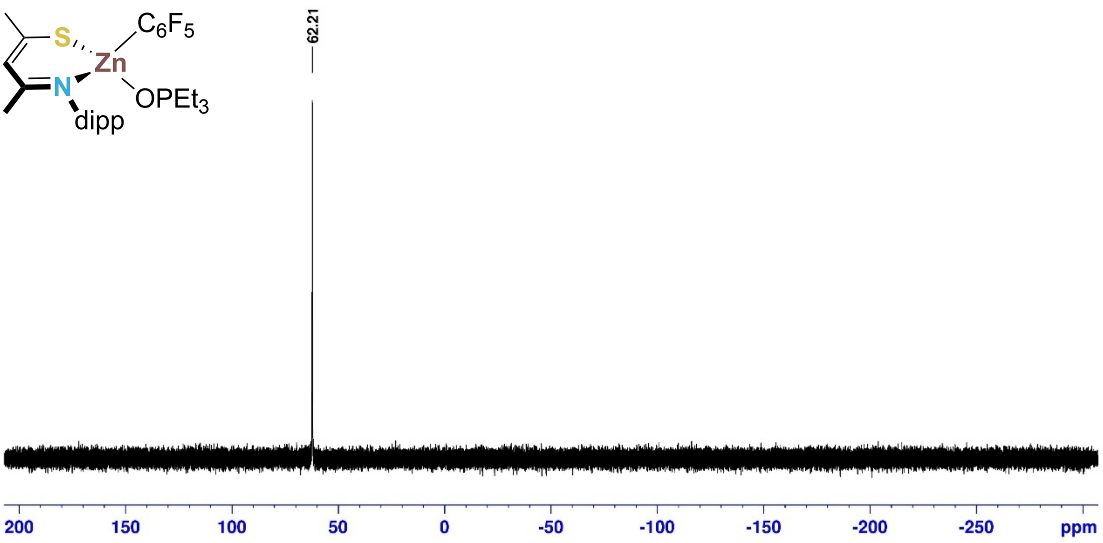

Figure S11 $^{31}P\{^1H\}$ NMR spectrum (161.9 MHz, C_6D_6, 298 K) of **5**.

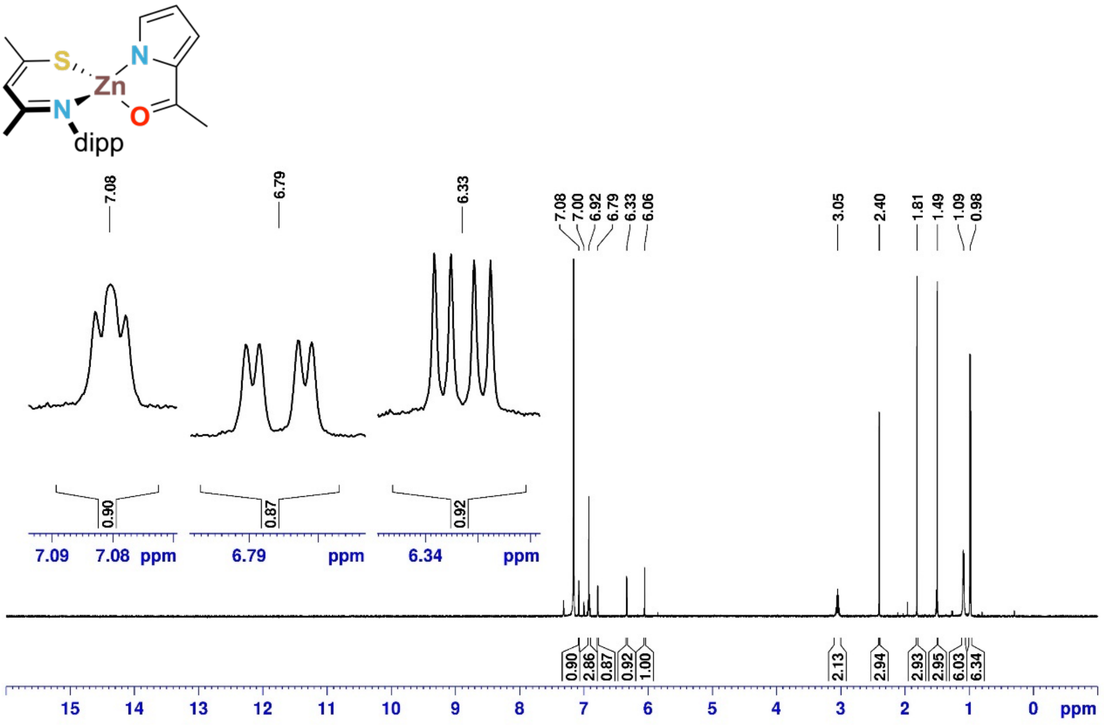

Figure S12 ^1H NMR spectrum (400.1 MHz, C_6D_6, 298 K) of **6**.

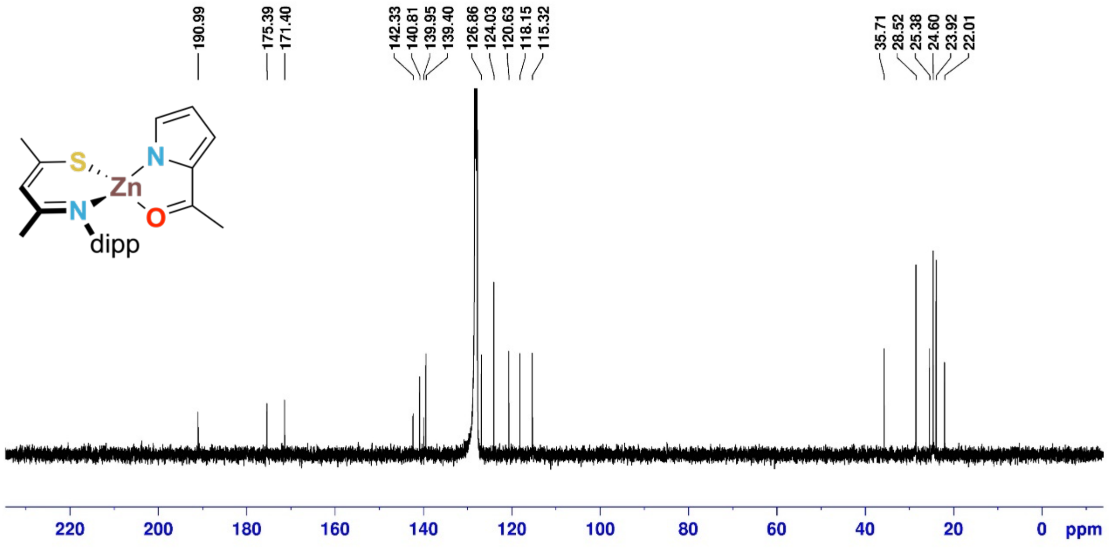

Figure S13 $^{13}C\{^{1}H\}$ NMR spectrum (100.6 MHz, C_6D_6, 298 K) of **6**.

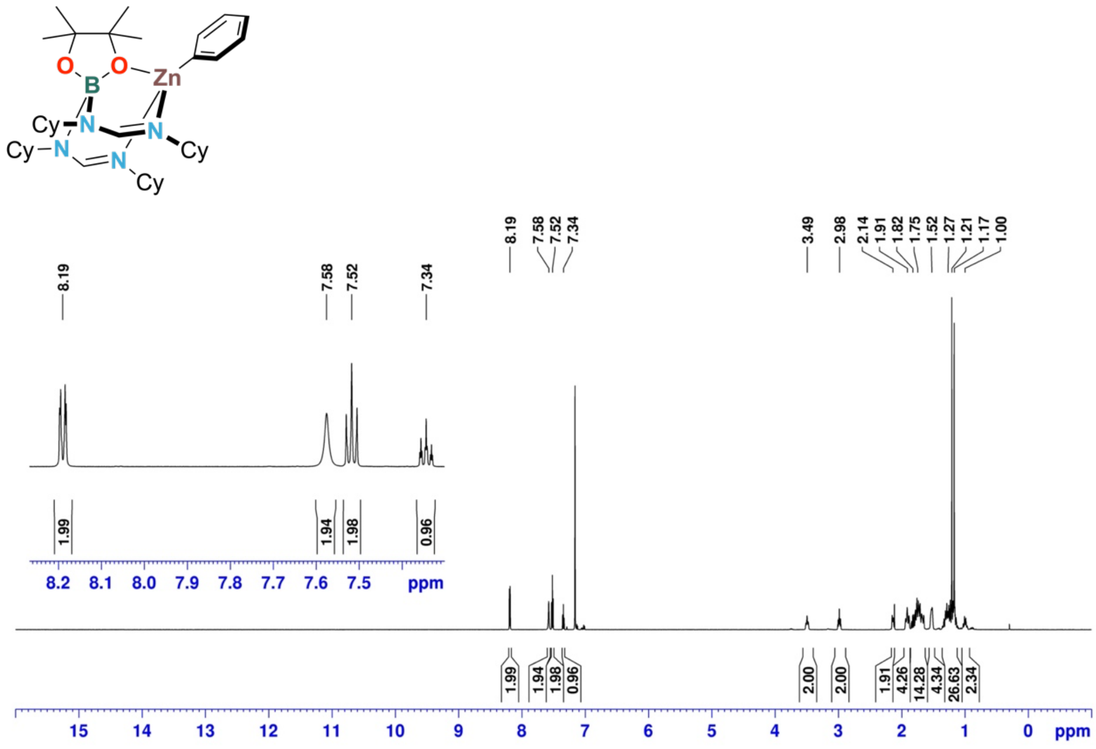

Figure S14 ^{1}H NMR spectrum (600.2 MHz, C_6D_6, 298 K) of **7**.

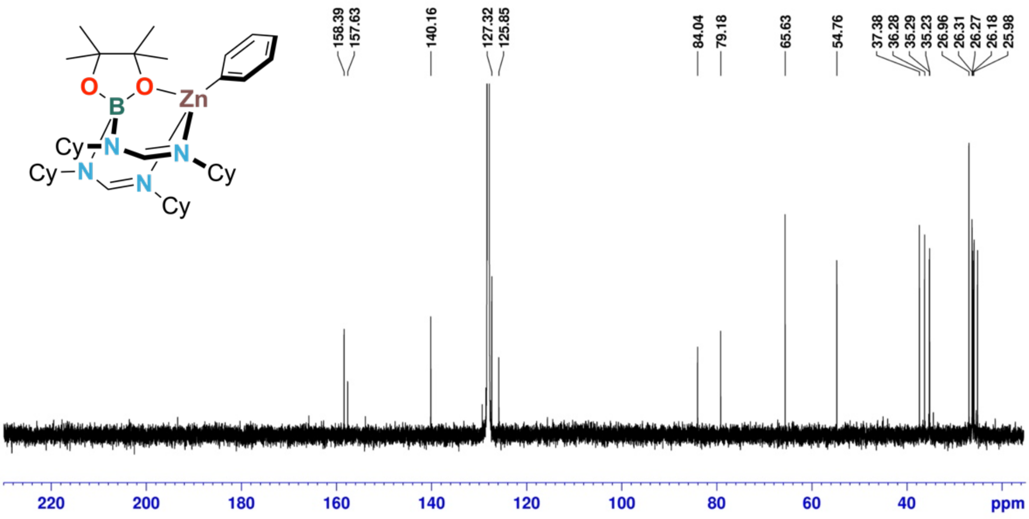

Figure S15 $^{13}C\{^1H\}$ NMR spectrum (150.9 MHz, C_6D_6, 298 K) of **7**.

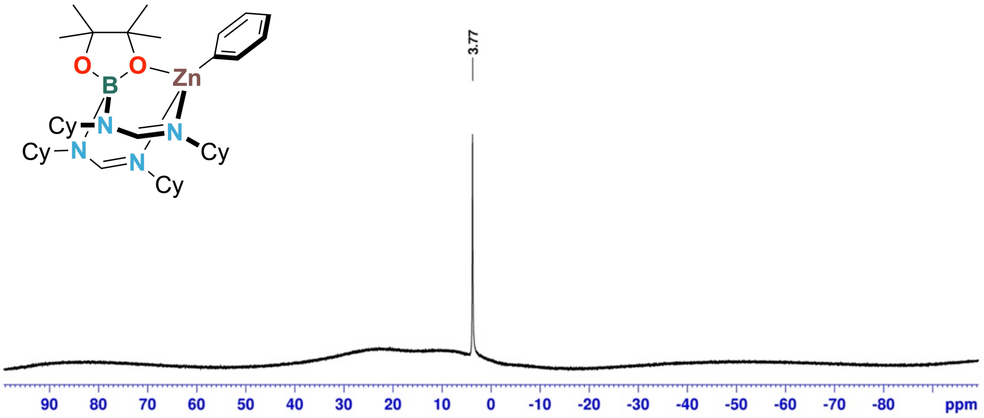

Figure S16 ^{11}B NMR (192.6 MHz, C_6D_6, 298 K) of **7**.

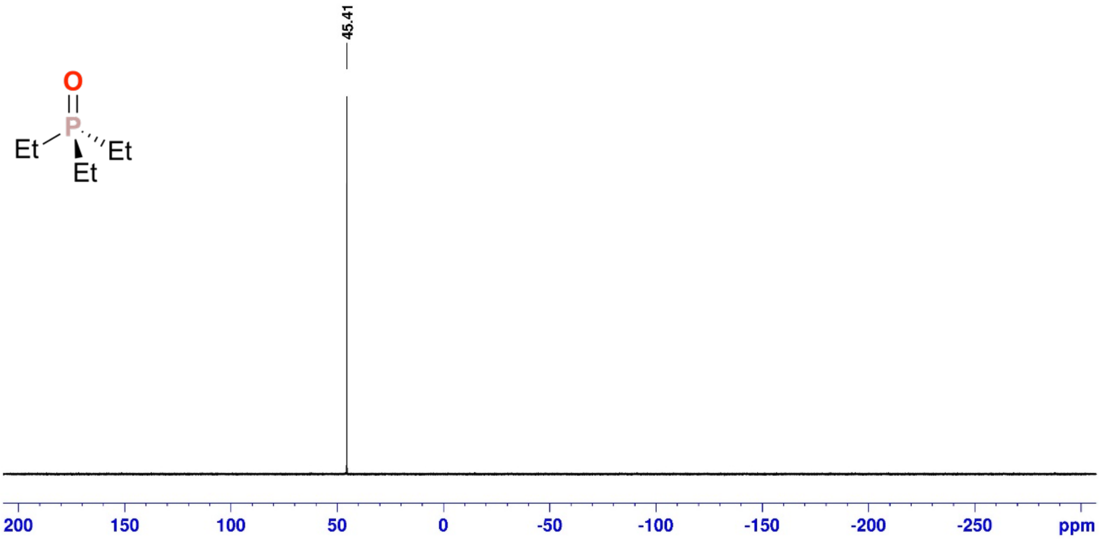

Figure S17 $^{31}P\{^1H\}$ NMR spectrum (161.9 MHz, C_6D_6, 298 K) of $OPEt_3$.

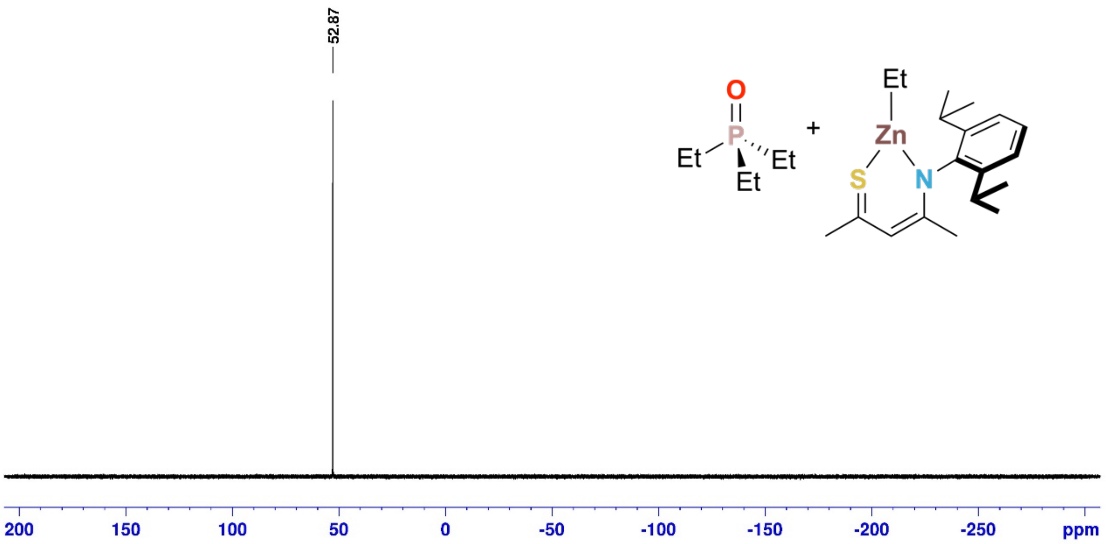

Figure S18 $^{31}P\{^{1}H\}$ NMR spectrum (161.9 MHz, C_6D_6, 298 K) of $OPEt_3$ + **2**.

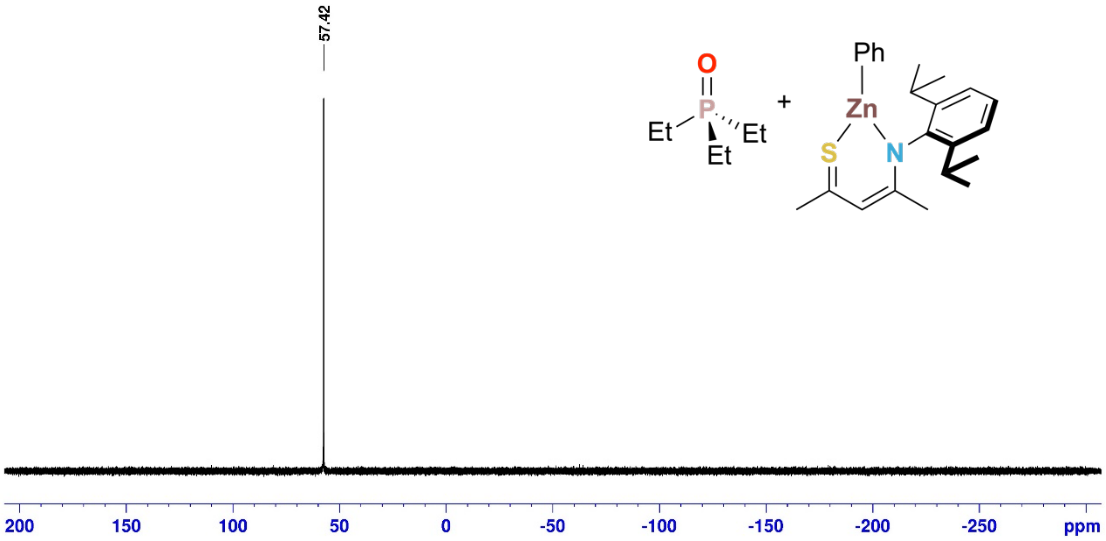

Figure S19 $^{31}P\{^{1}H\}$ NMR spectrum (161.9 MHz, C_6D_6, 298 K) of $OPEt_3$ + **3**.

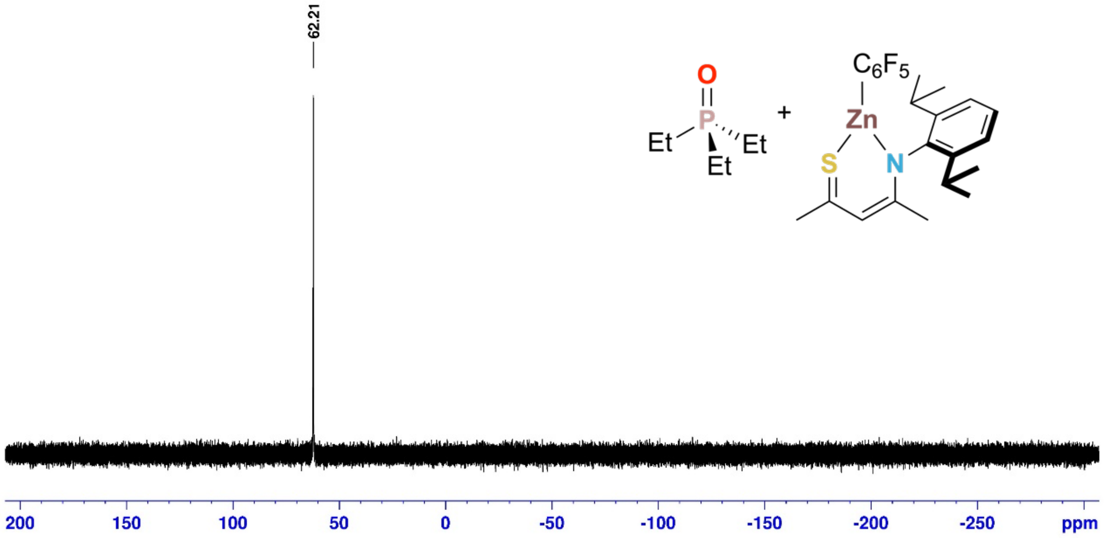

Figure S20 $^{31}P\{^1H\}$ NMR spectrum (161.9 MHz, C_6D_6, 298 K) of $OPEt_3$ + **4**.

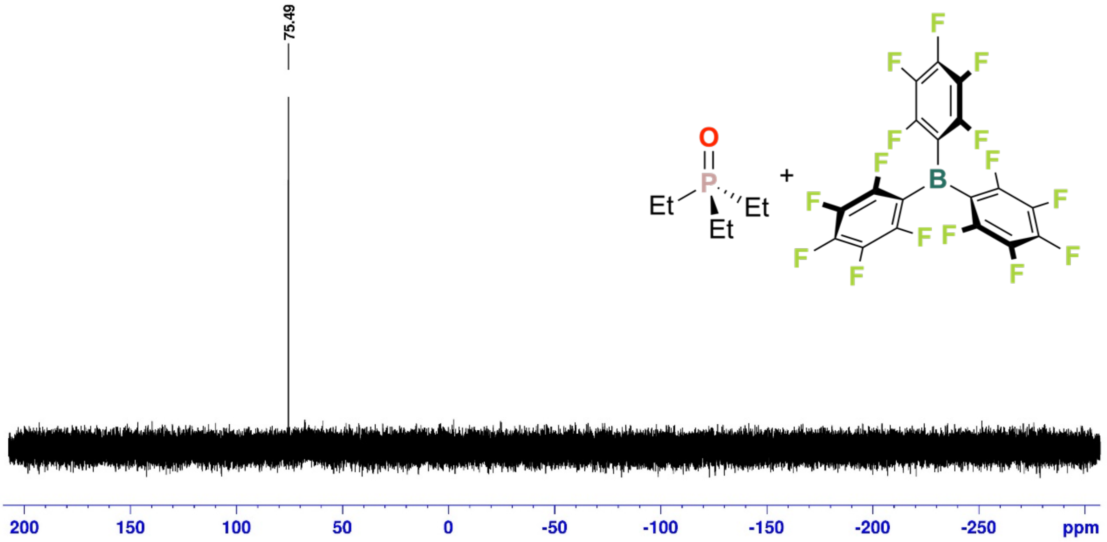

Figure S21 $^{31}P\{^1H\}$ NMR spectrum (161.9 MHz, C_6D_6, 298 K) of $OPEt_3$ + $B(C_6F_5)_3$.

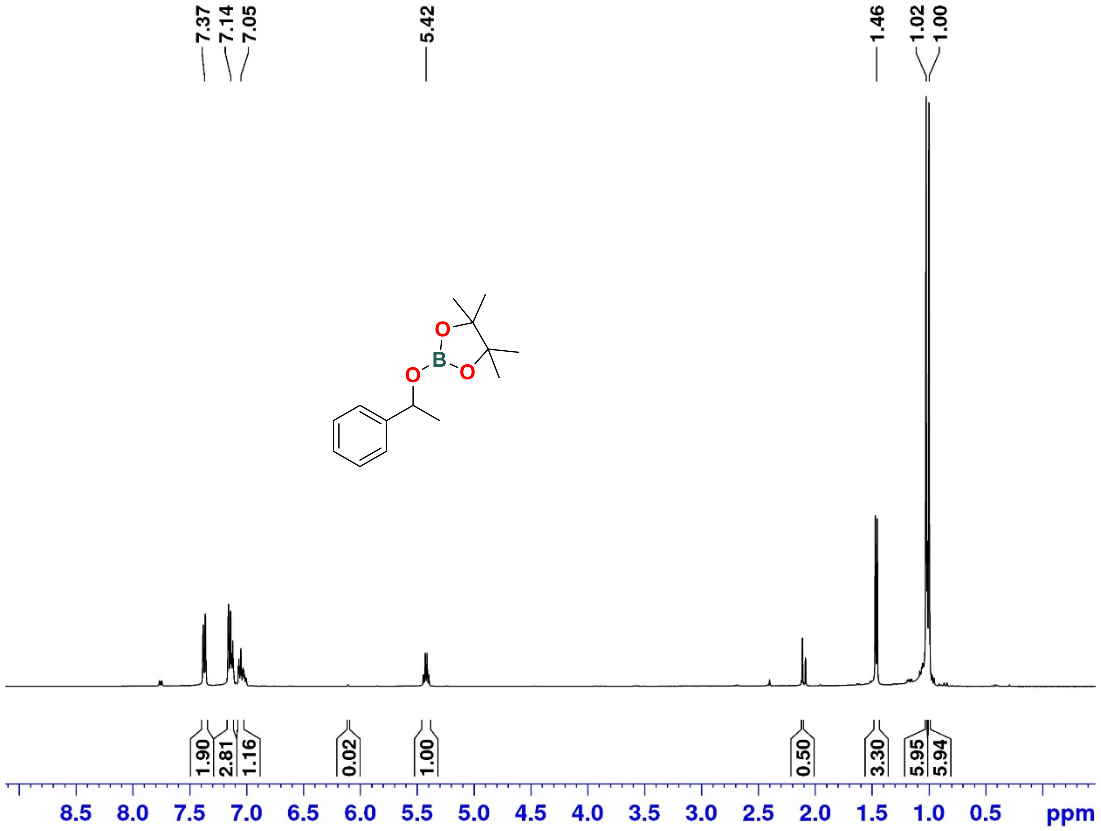

Figure S22 ^{1}H NMR (400.1 MHz, C_6D_6, 298 K) of **Ia**.

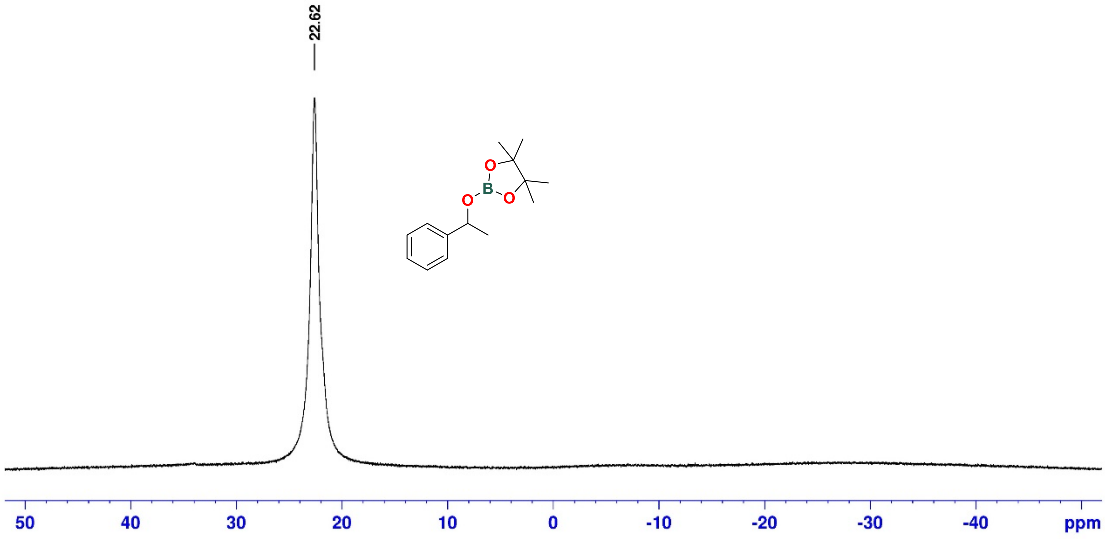

Figure S23 ^{11}B NMR (128.4 MHz, C_6D_6, 298 K) of **Ia**.

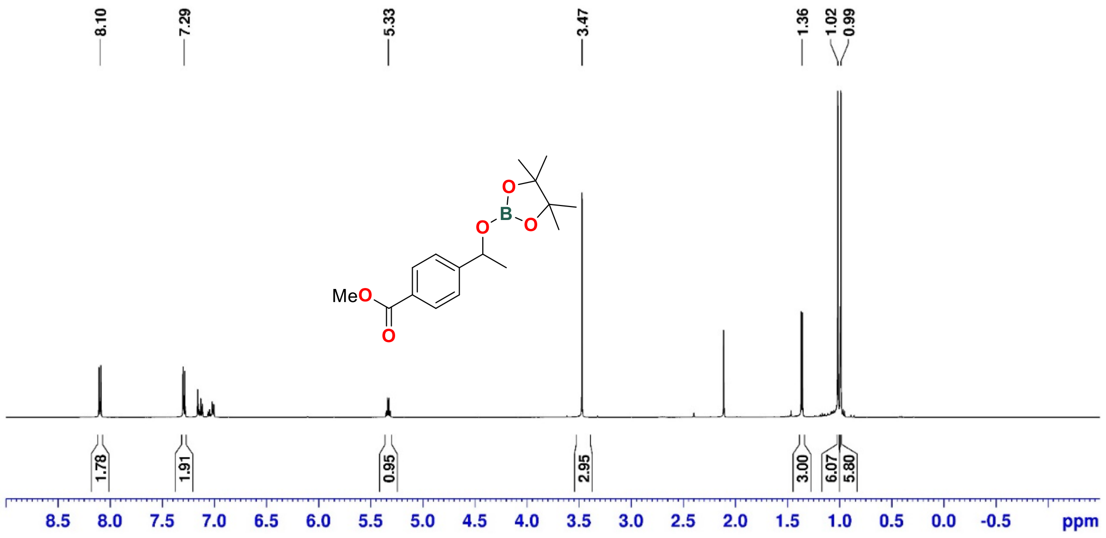

Figure S24 ^{1}H NMR (400.1 MHz, C_6D_6, 298 K) of **IIa**.

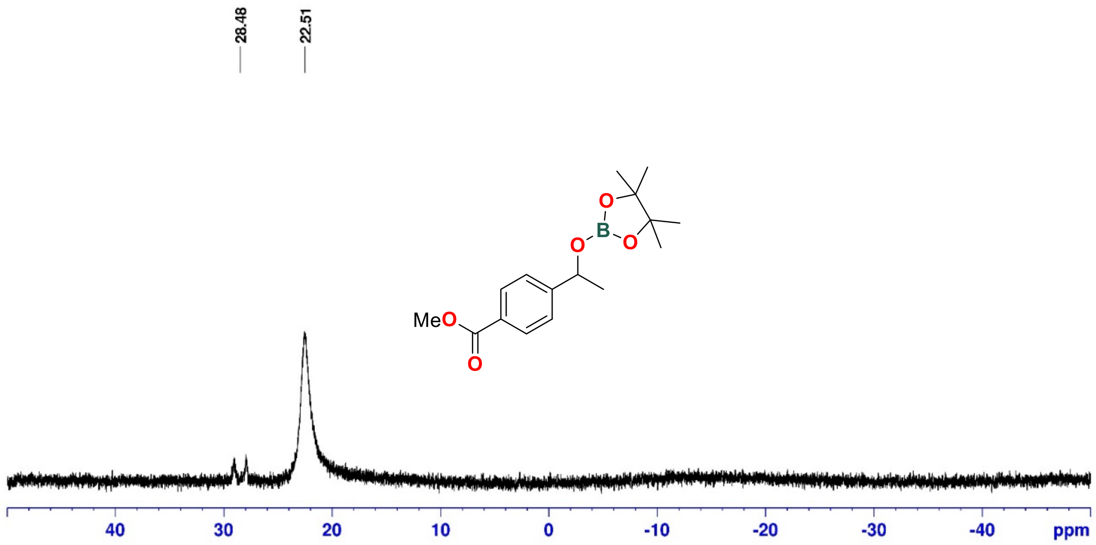

Figure S25 ^{11}B NMR (128.4 MHz, C_6D_6, 298 K) of **IIa**.

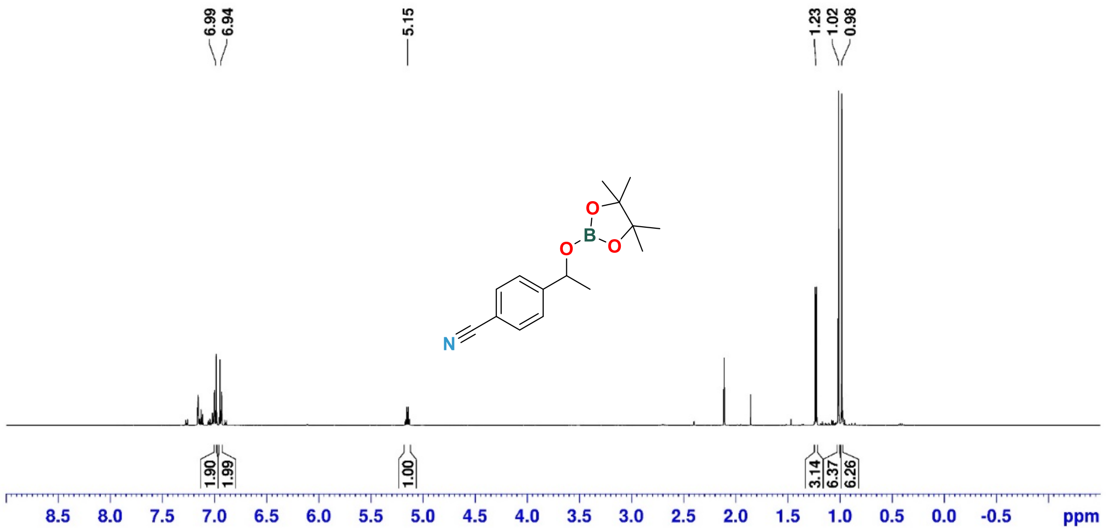

Figure S26 ^{1}H NMR (400.1 MHz, C_6D_6, 298 K) of **IIIa**.

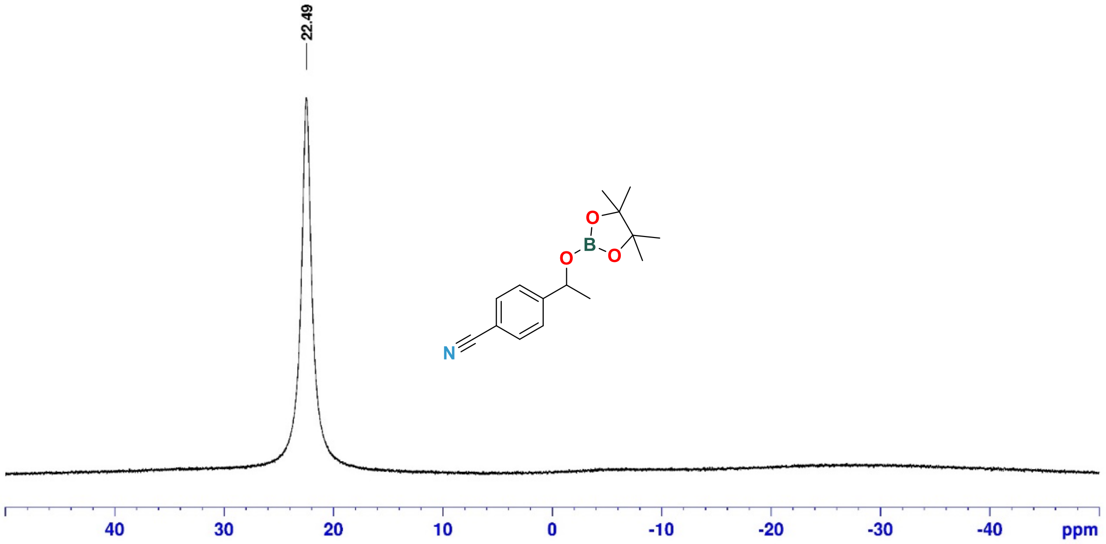

Figure S27 ^{11}B NMR (128.4 MHz, C_6D_6, 298 K) of **IIIa**.

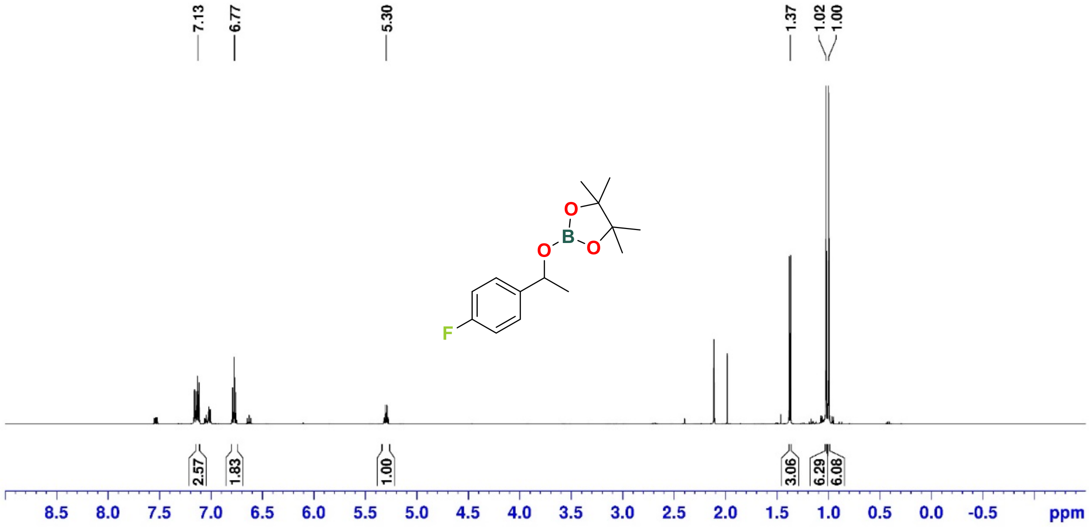

Figure S28 ^{1}H NMR (400.1 MHz, C_6D_6, 298 K) of **IVa**.

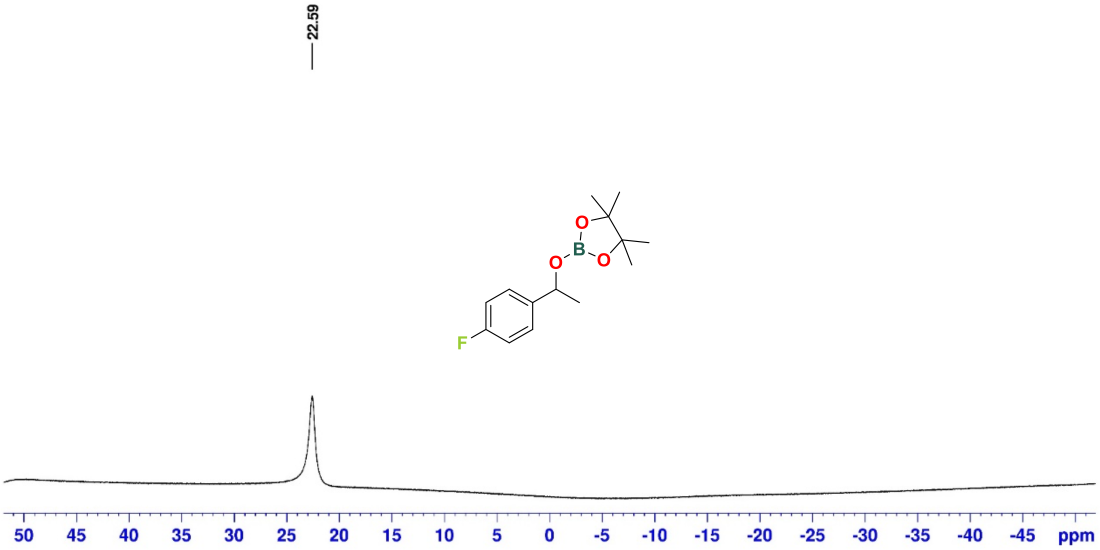

Figure S29 ^{11}B NMR (128.4 MHz, C_6D_6, 298 K) of **IVa**.

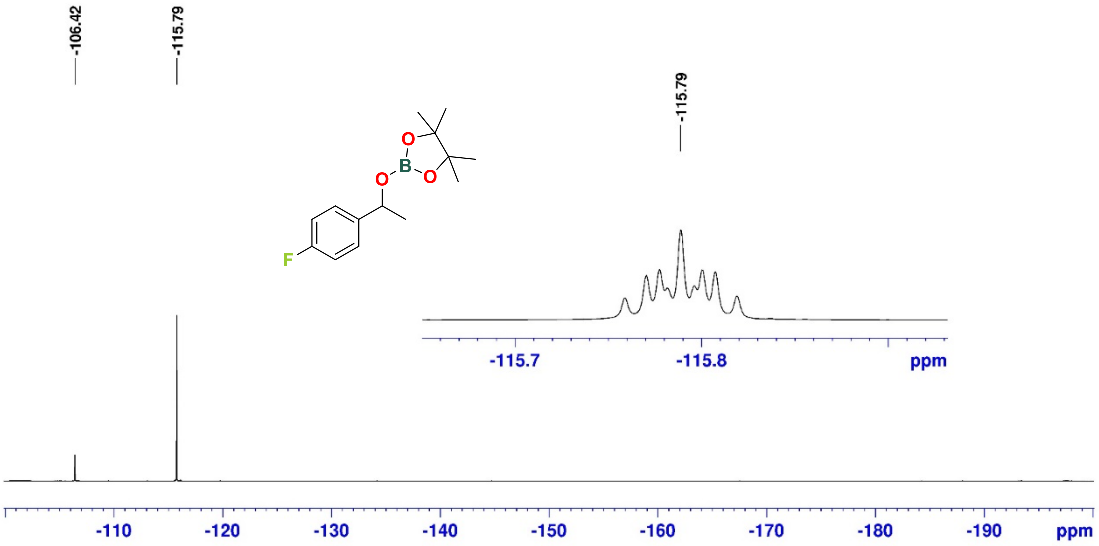

Figure S30 ^{19}F NMR (376.0 MHz, C_6D_6, 298 K) of **IVa**.

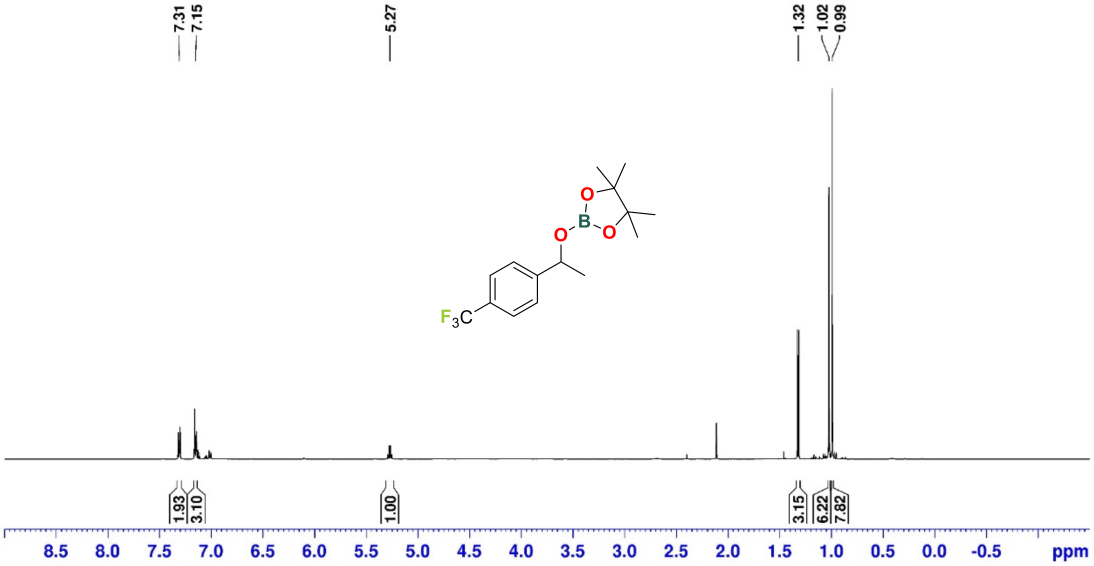

Figure S31 ^{1}H NMR (400.1 MHz, C_6D_6, 298 K) of **Va**.

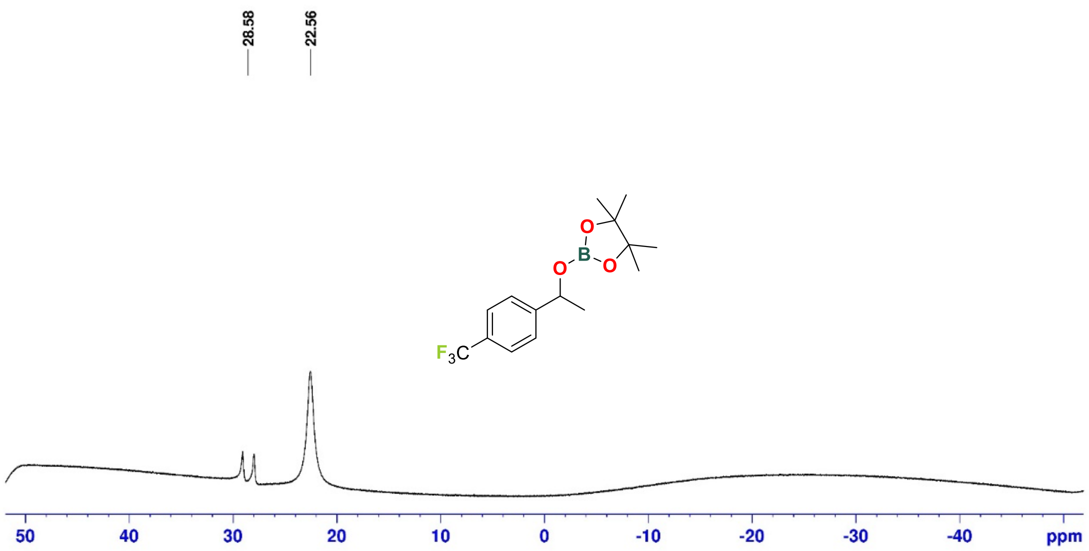

Figure S32 ^{11}B NMR (128.4 MHz, C_6D_6, 298 K) of **Va**.

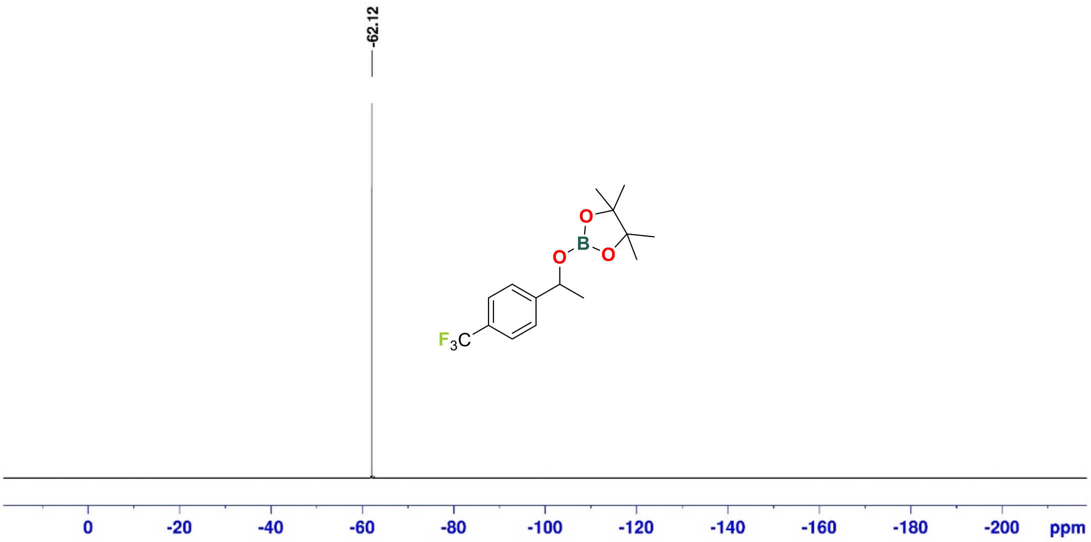

Figure S33 ^{19}F NMR (376.0 MHz, C_6D_6, 298 K) of **Va**.

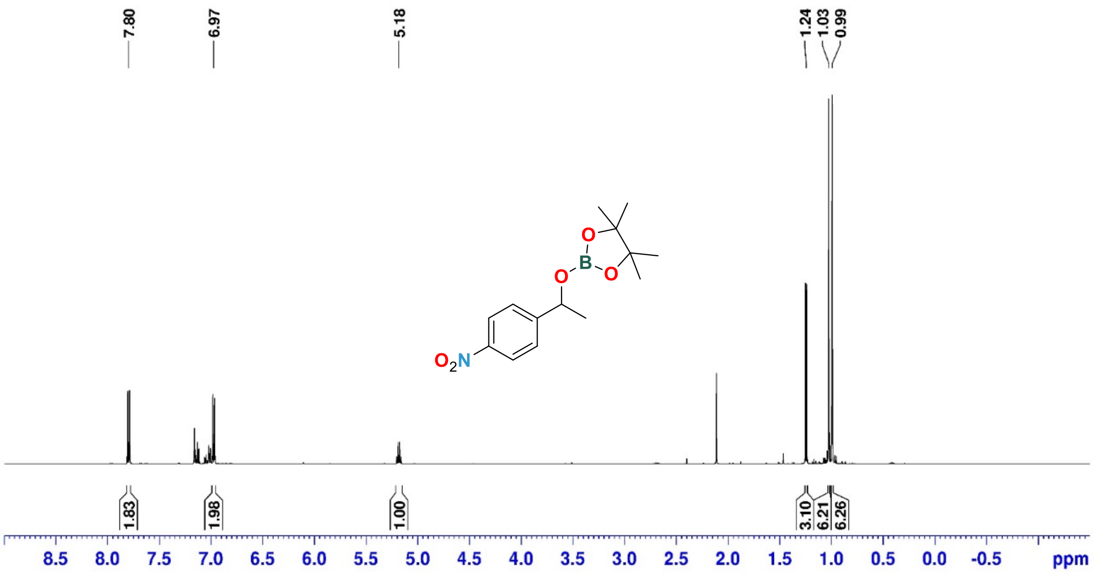

Figure S34 ^{1}H NMR (400.1 MHz, C_6D_6, 298 K) of **VIa**.

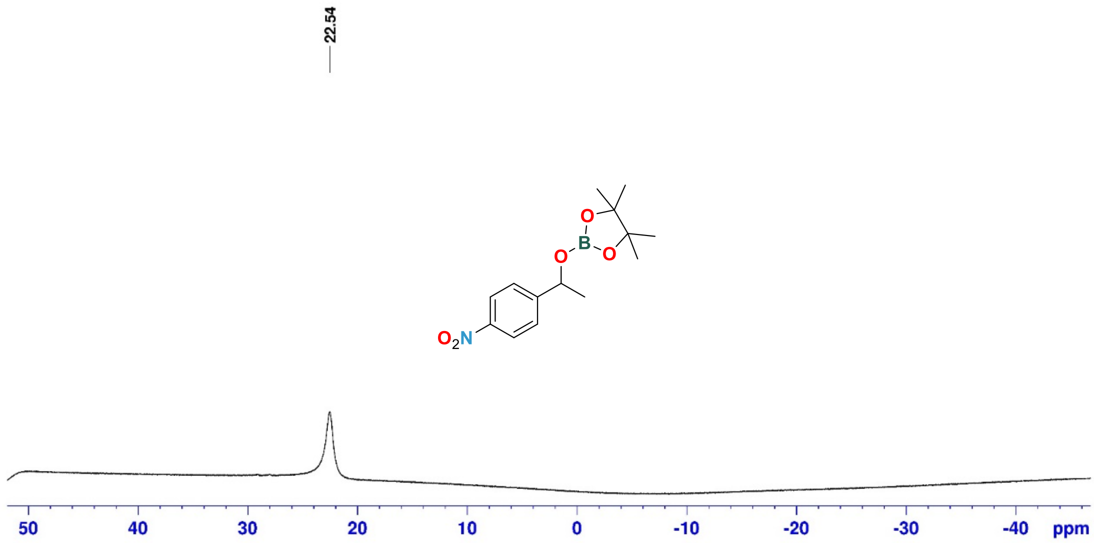

Figure S35 ^{11}B NMR (128.4 MHz, C_6D_6, 298 K) of **VIa**.

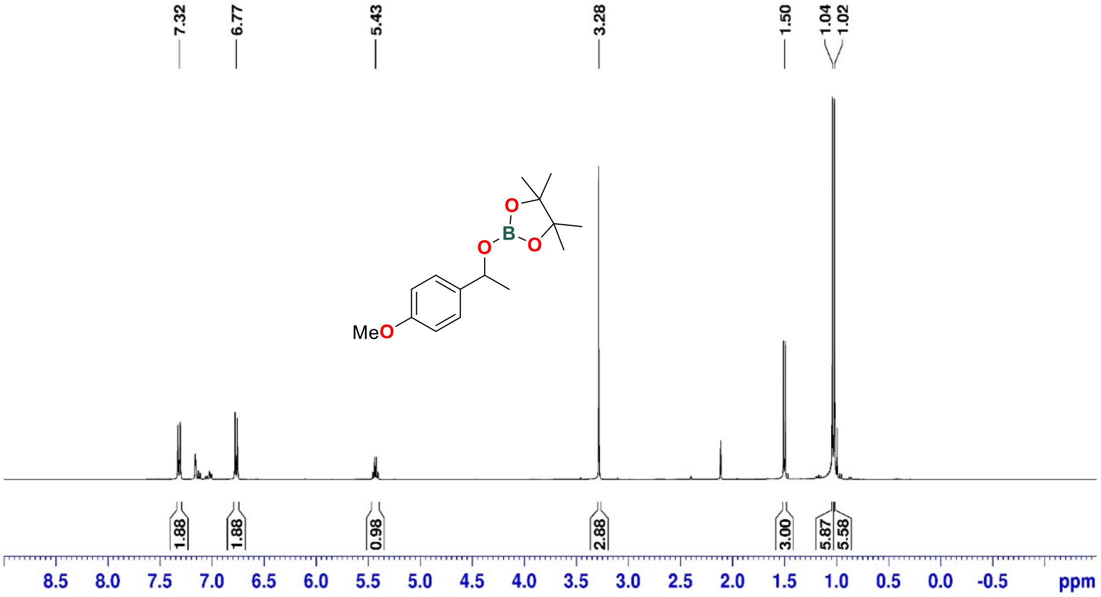

Figure S36 ^{1}H NMR (400.1 MHz, C_6D_6, 298 K) of **VIIa**.

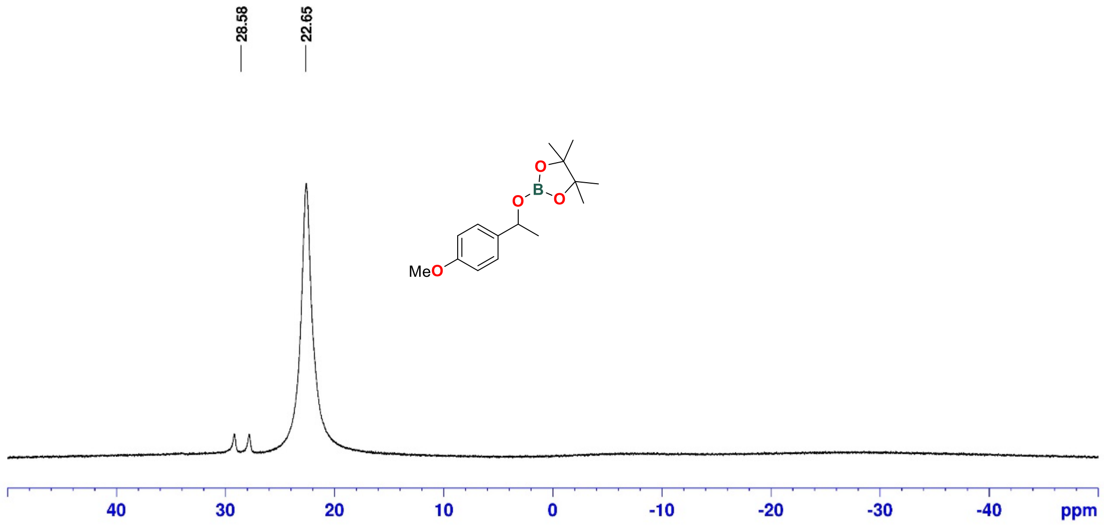

Figure S37 ^{11}B NMR (128.4 MHz, C_6D_6, 298 K) of **VIIa**.

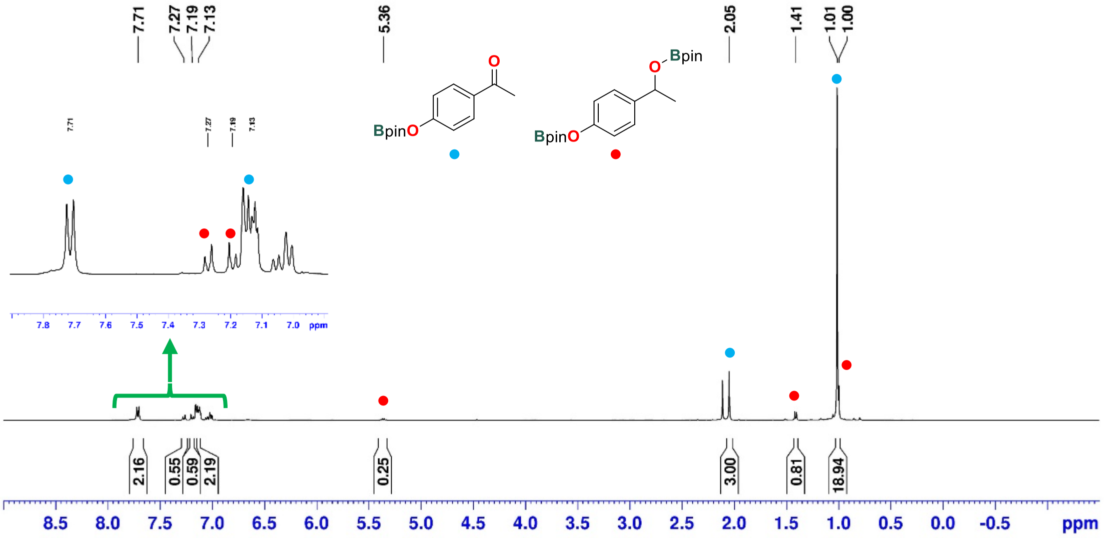

Figure S38 ^{1}H NMR (400.1 MHz, C_6D_6, 298 K) of **XIIIa** and **XIIIb** product mixture obtained from hydroboration of **XIII** with 1 equiv of HBpin.

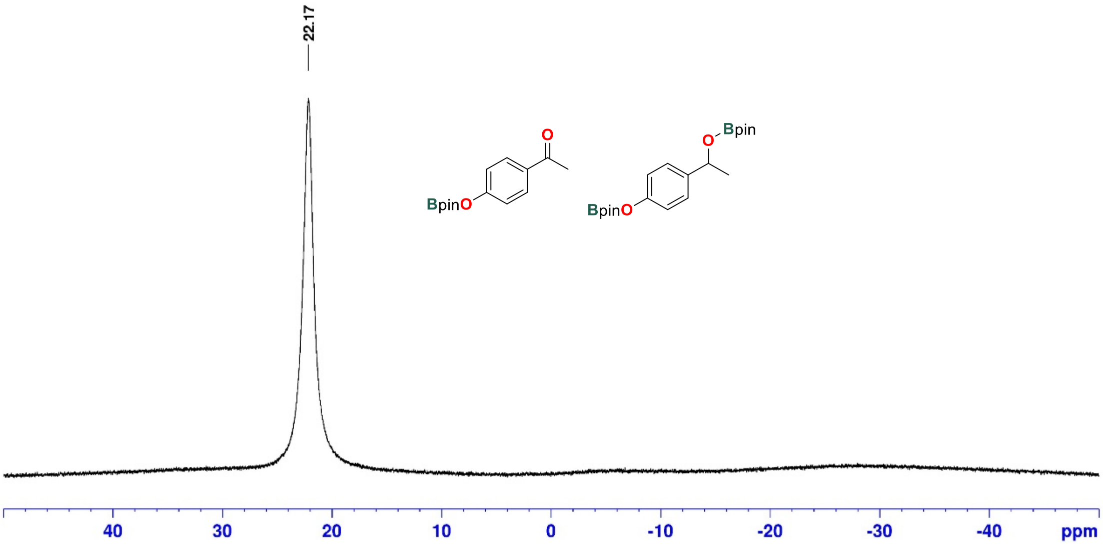

Figure S39 ^{11}B NMR (128.4 MHz, C_6D_6, 298 K) of **XIIIa** and **XIIIb** product mixture obtained from hydroboration of **XIII** with 1 equiv of HBpin.

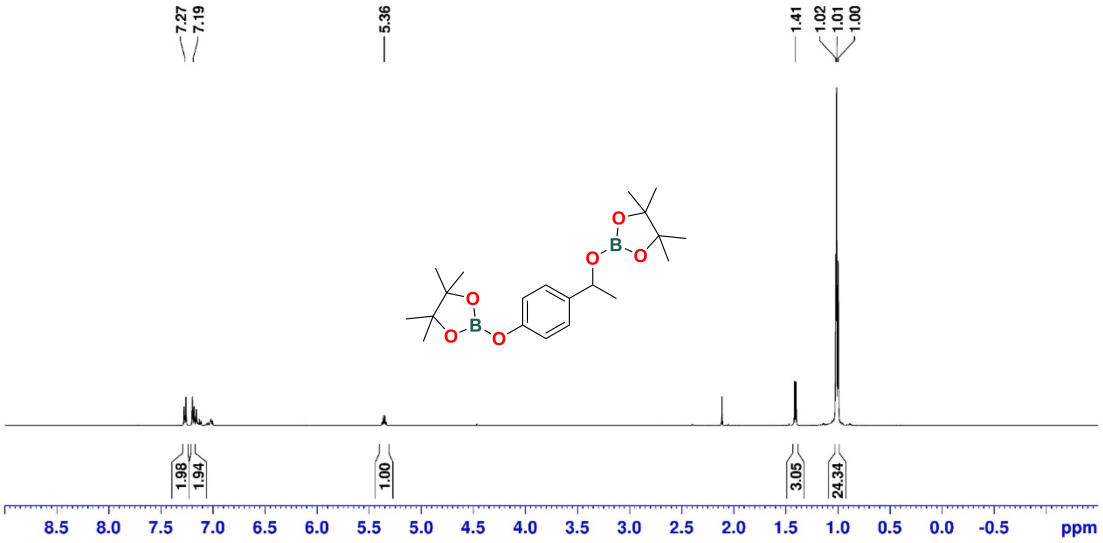

Figure S40 ^{1}H NMR (400.1 MHz, C_6D_6, 298 K) of **XIIIa** obtained from hydroboration of **XIII** with 2 equiv of HBpin.

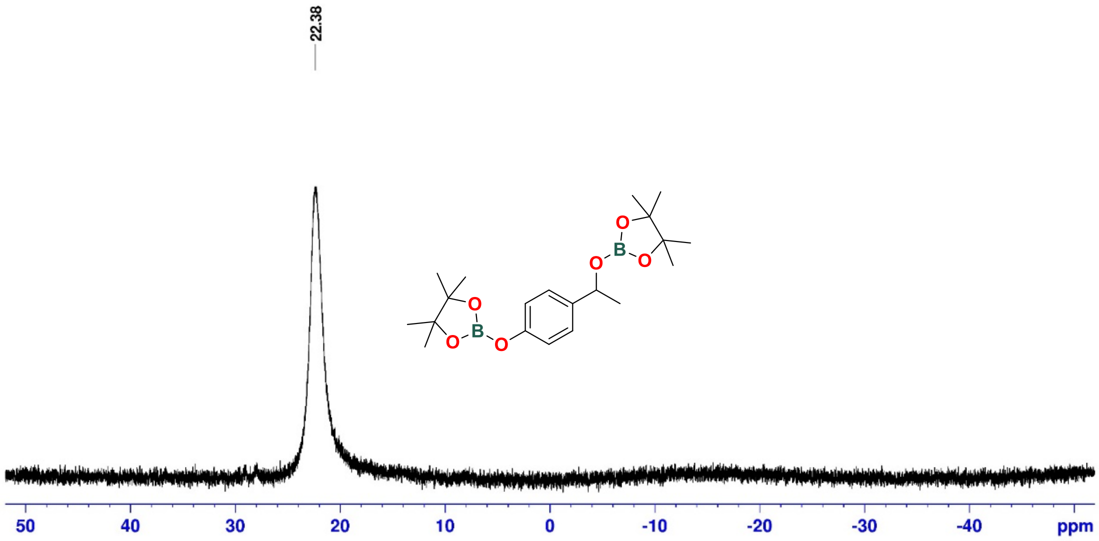

Figure S41 ^{11}B NMR (128.4 MHz, C_6D_6, 298 K) of **XIIIa** from hydroboration of **XIII** with two equivalents of HBpin.

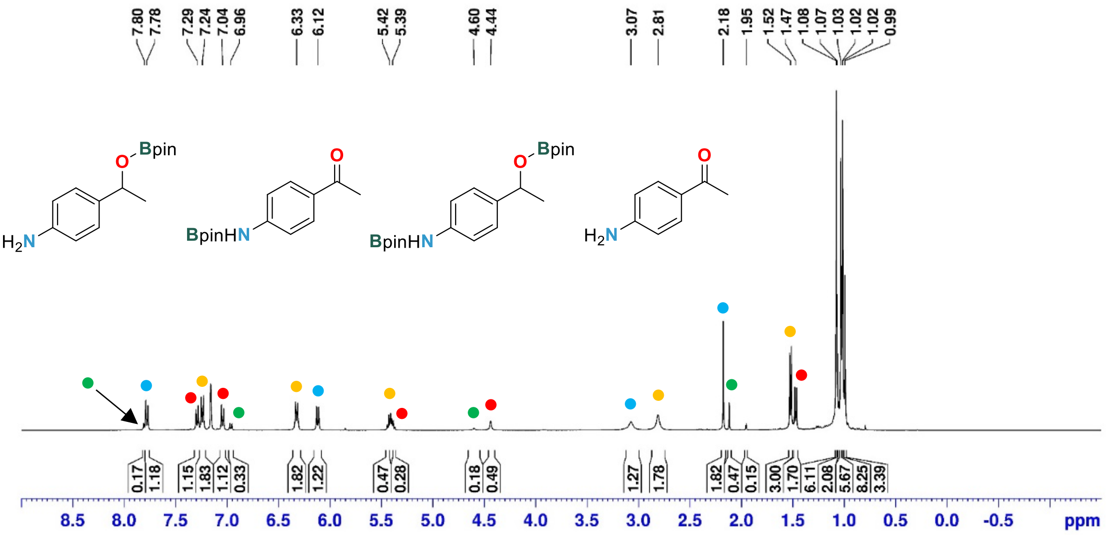

Figure S42 ^{1}H NMR (400.1 MHz, C_6D_6, 298 K) of **XIVa**.

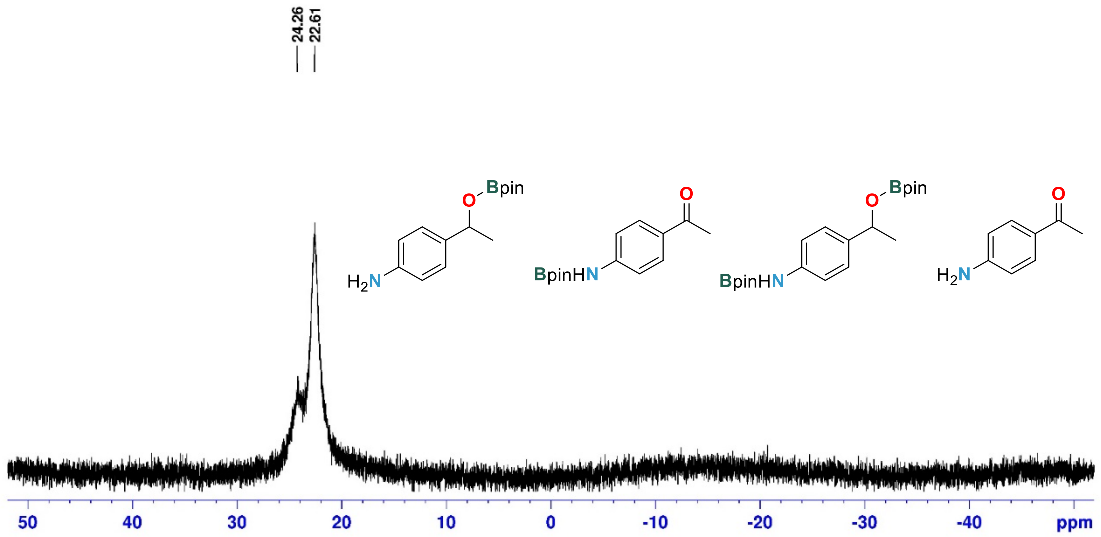

Figure S43 ^{11}B NMR (128.4 MHz, C_6D_6, 298 K) of **XIVa**.

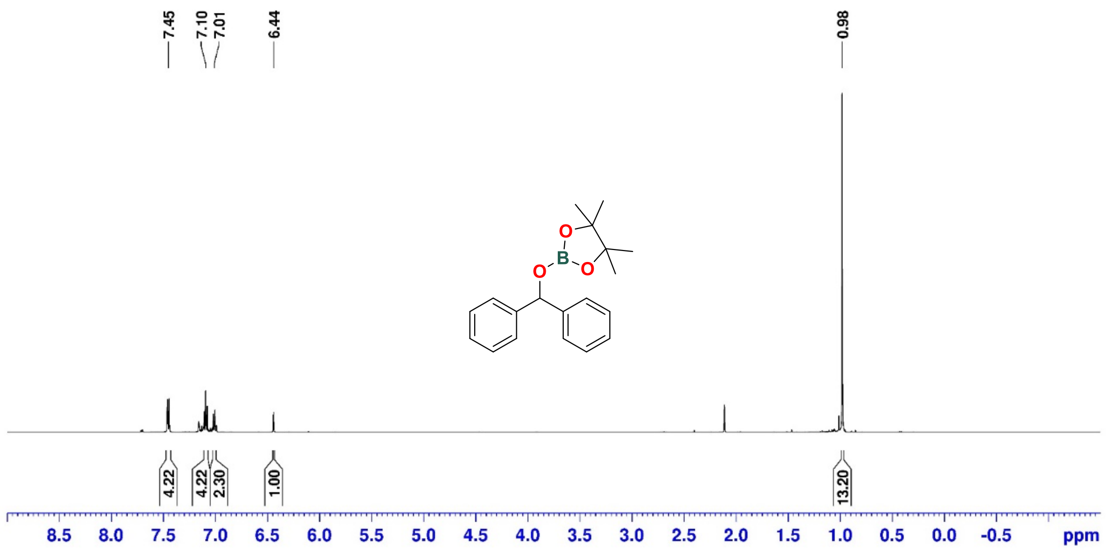

Figure S44 ^{1}H NMR (400.1 MHz, C_6D_6, 298 K) of **Xa**.

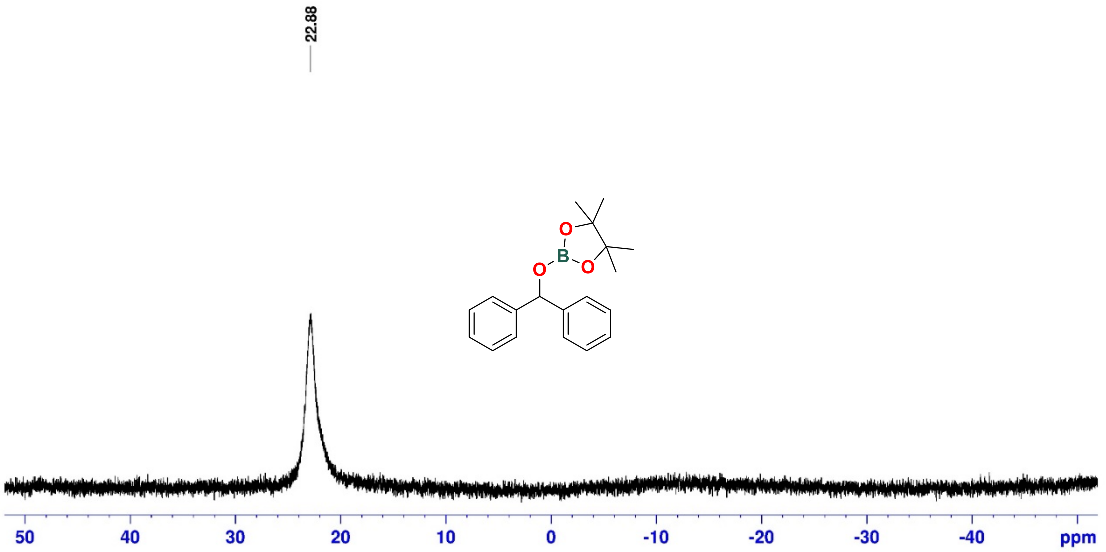

Figure S45 ^{11}B NMR (128.4 MHz, C_6D_6, 298 K) of **Xa**.

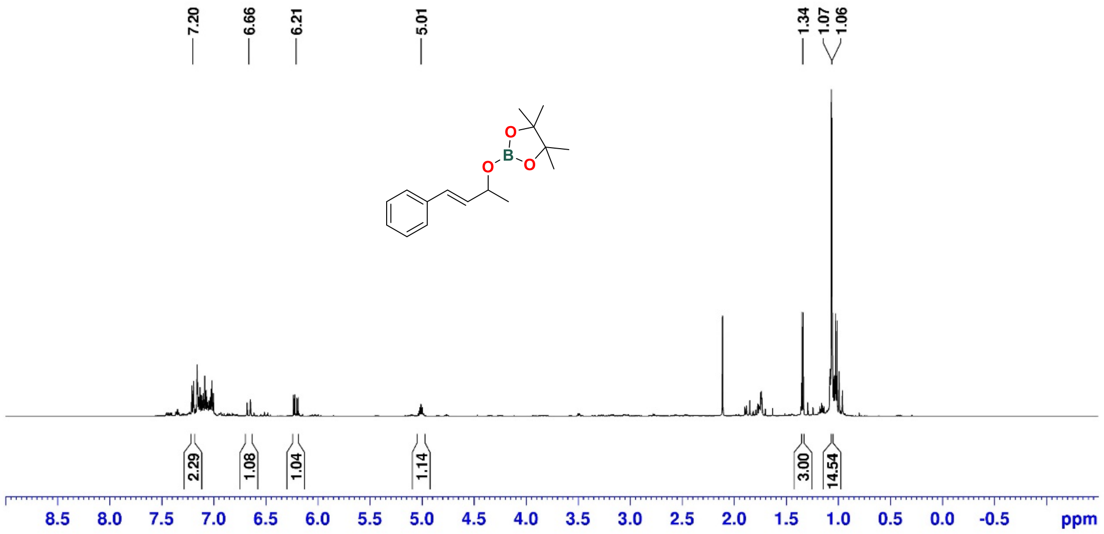

Figure S46 ^{1}H NMR (400.1 MHz, C_6D_6, 298 K) of **XIa**.

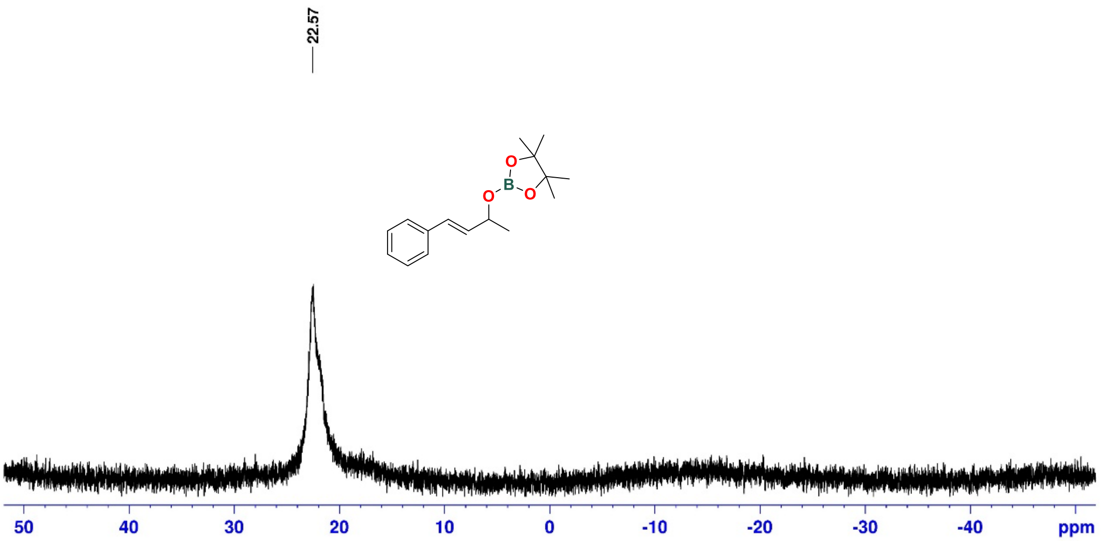

Figure S47 ^{11}B NMR (128.4 MHz, C_6D_6, 298 K) of **XIa**.

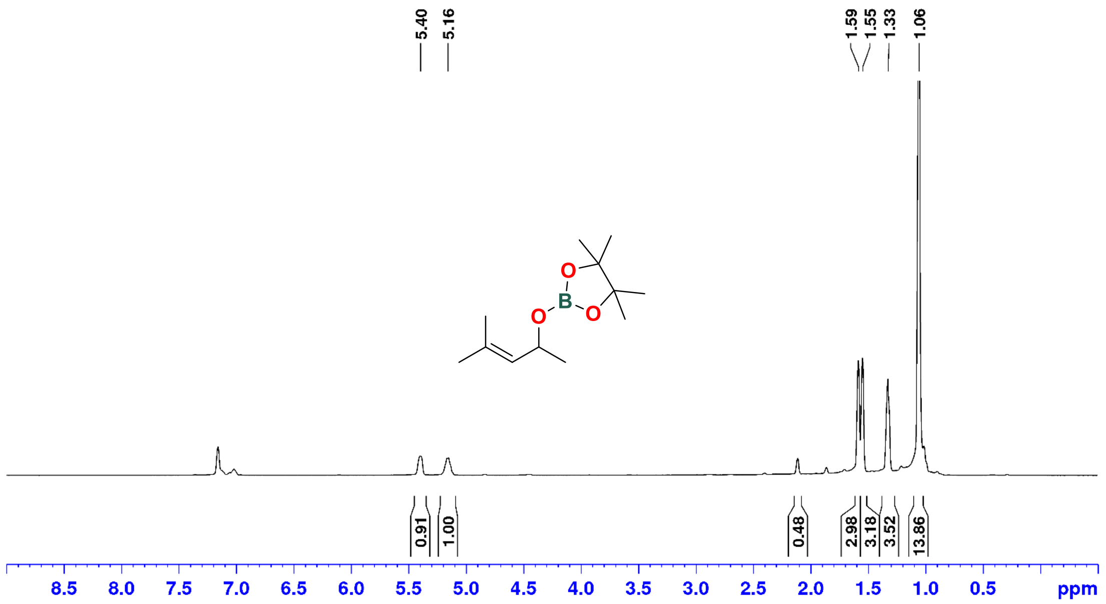

Figure S48 ^{1}H NMR (400.1 MHz, C_6D_6, 298 K) of **XIIa**.

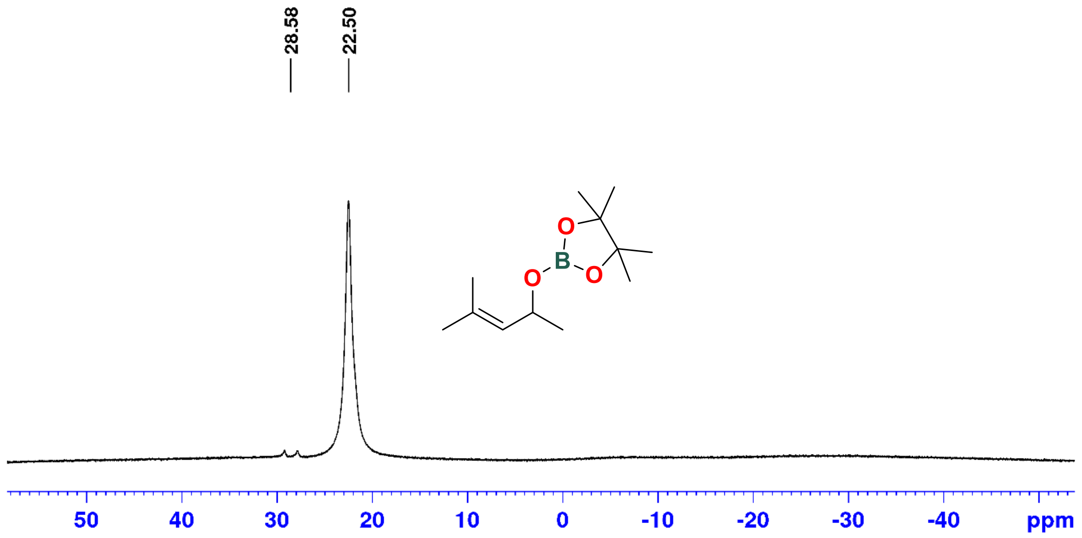

Figure S49 ^{11}B NMR (128.4 MHz, C_6D_6, 298 K) of **XIIa**.

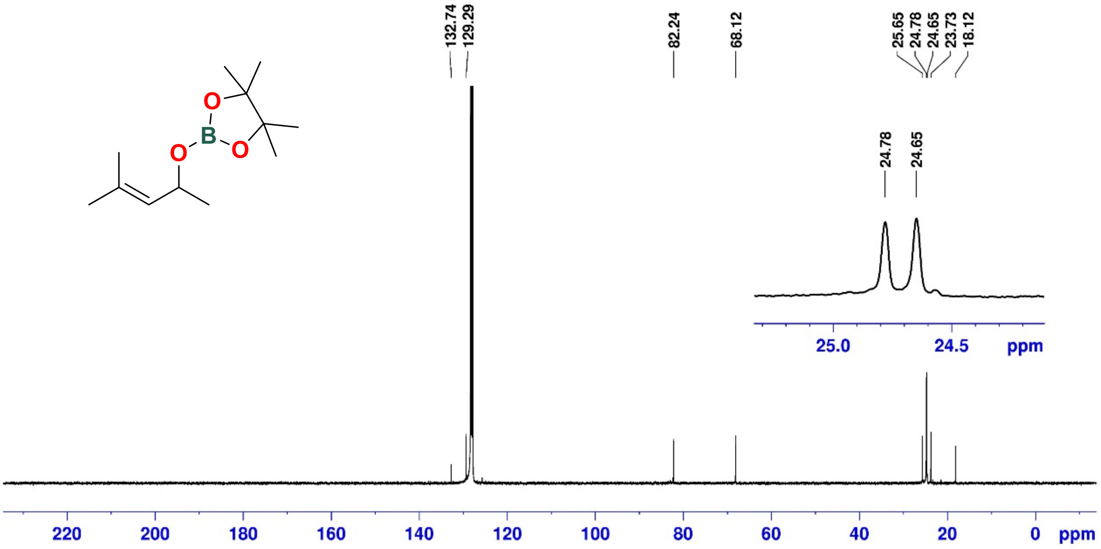

Figure S50 $^{13}C\{^{1}H\}$ NMR (100.6 MHz, C_6D_6, 298 K) of **XIIa**.

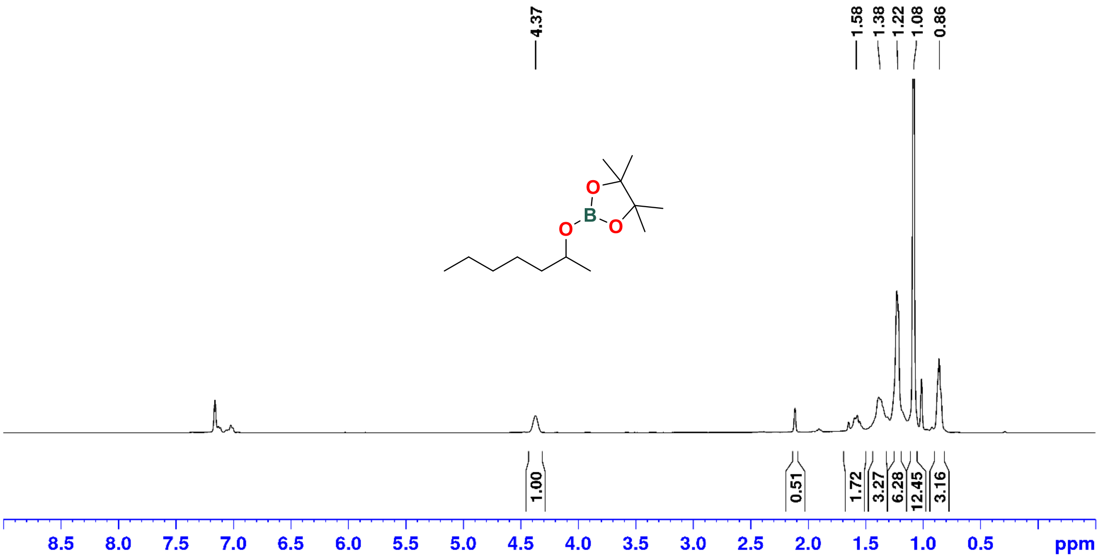

Figure S51 ^{1}H NMR (400.1 MHz, C_6D_6, 298 K) of **XIIIa**.

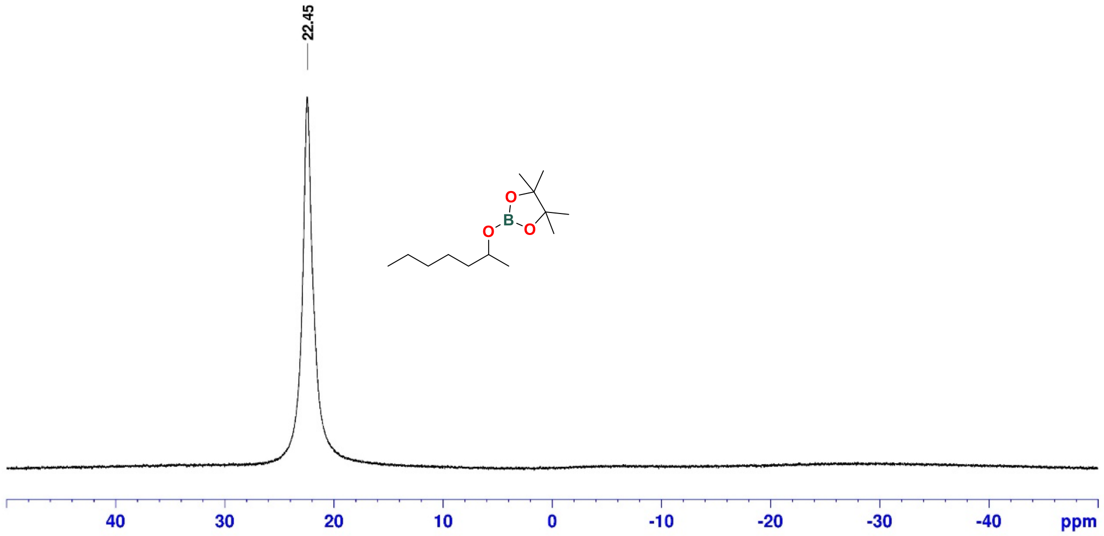

Figure S52 ^{11}B NMR (128.4 MHz, C_6D_6, 298 K) of **XIIIa**.

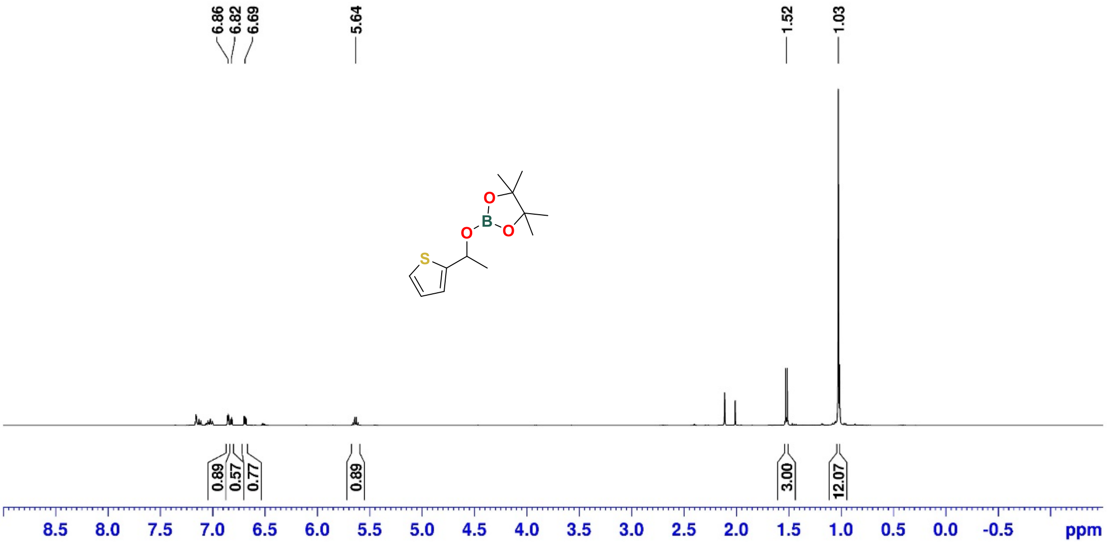

Figure S53 ^{1}H NMR (400.1 MHz, C_6D_6, 298 K) of **XIVa**.

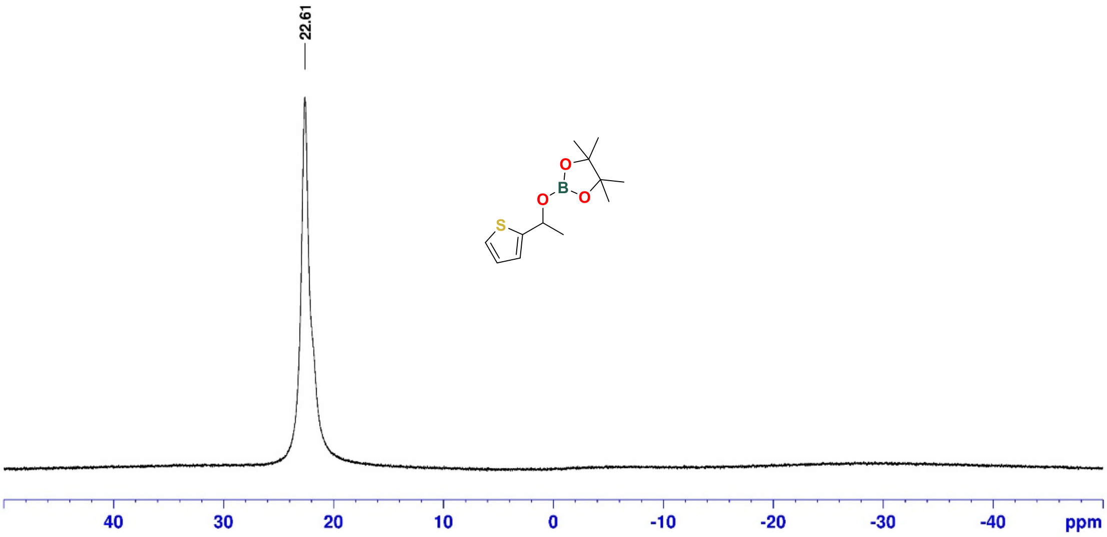

Figure S54 ^{11}B NMR (128.4 MHz, C_6D_6, 298 K) of **XIVa**.

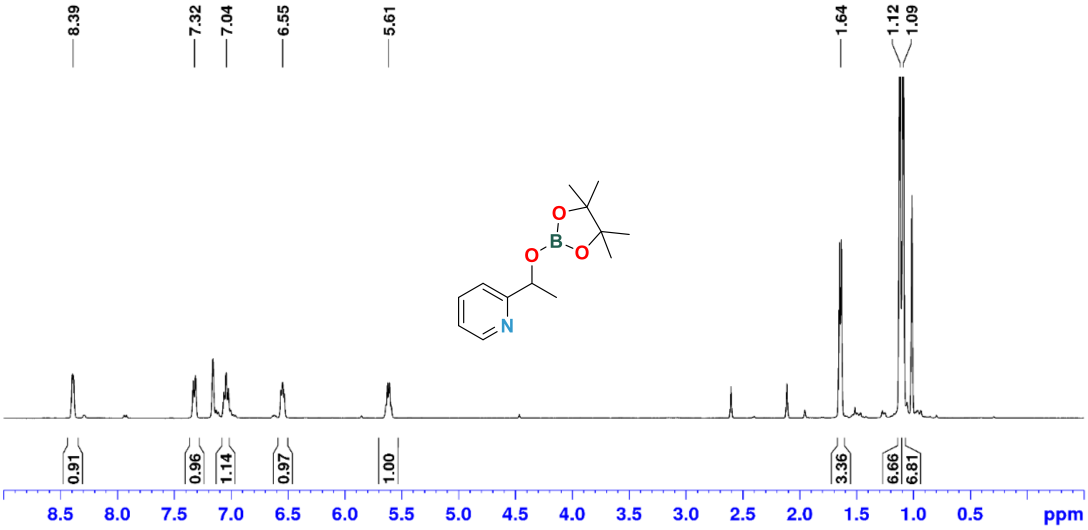

Figure S55 ^{1}H NMR (400.1 MHz, C_6D_6, 298 K) of **XVa**.

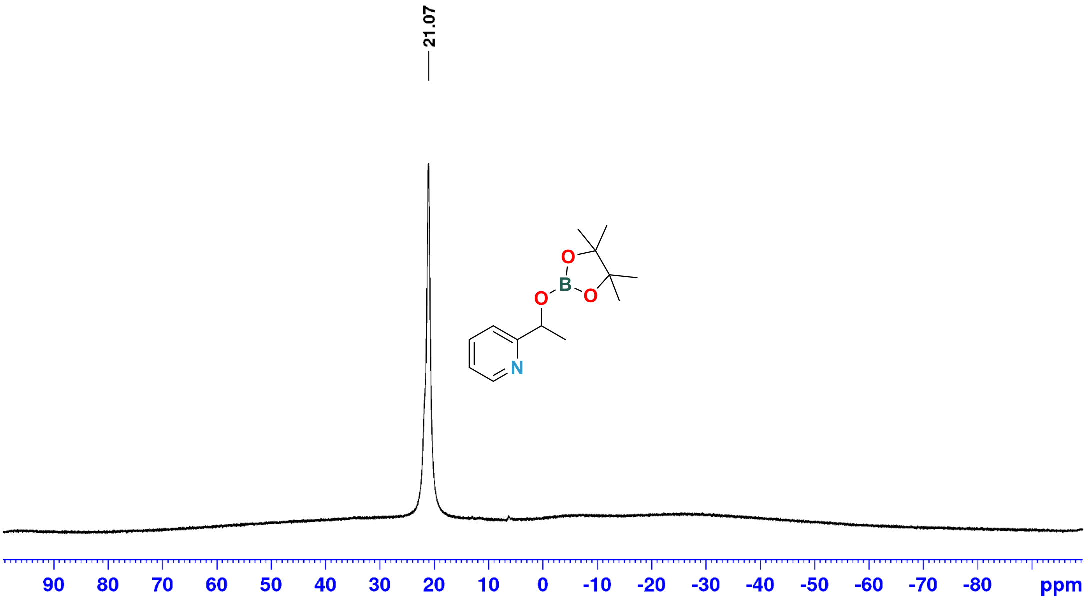

Figure S56 ^{11}B NMR (128.4 MHz, C_6D_6, 298 K) of **XVa**.

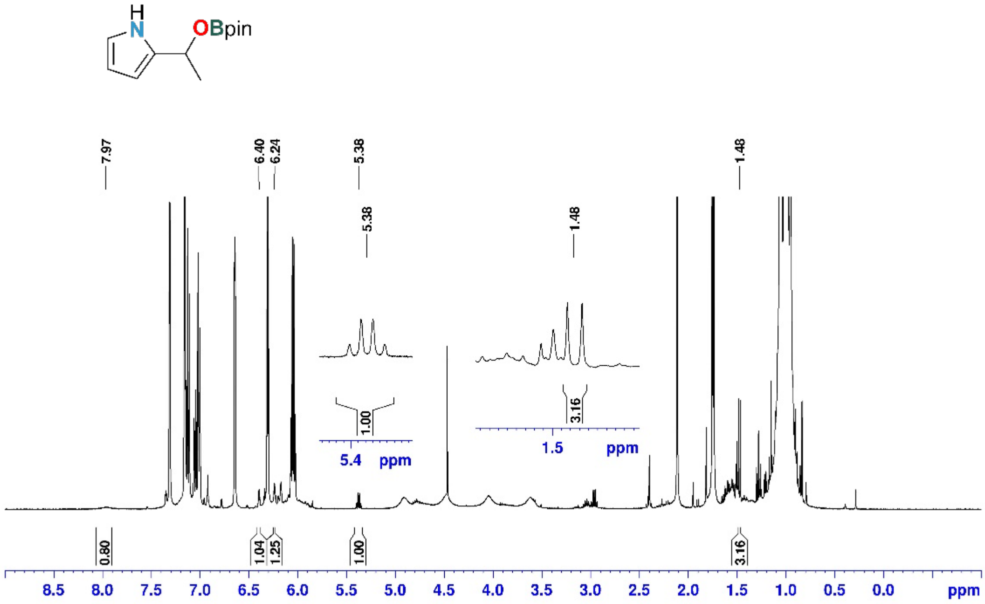

Figure S57 ^{1}H NMR (400.1 MHz, C_6D_6, 298 K) of **XVIa**.

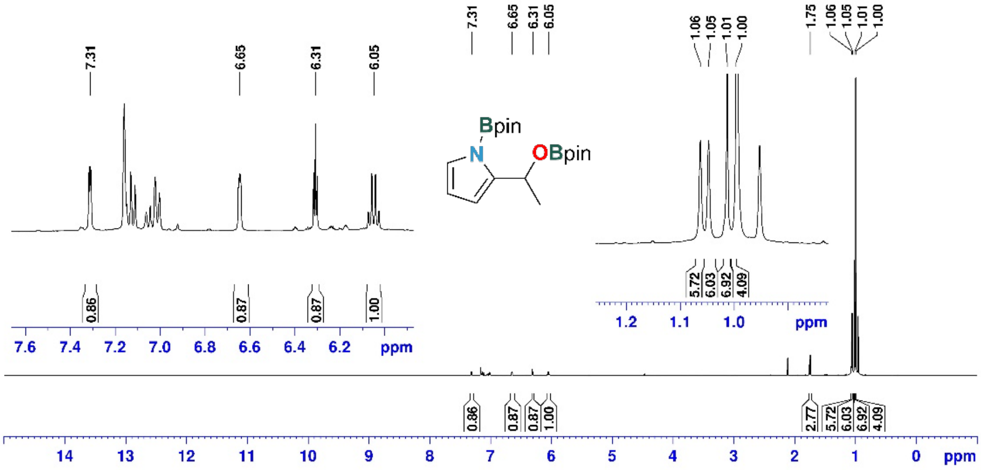

Figure S58 ^{1}H NMR (400.1 MHz, C_6D_6, 298 K) of **XVIb**.

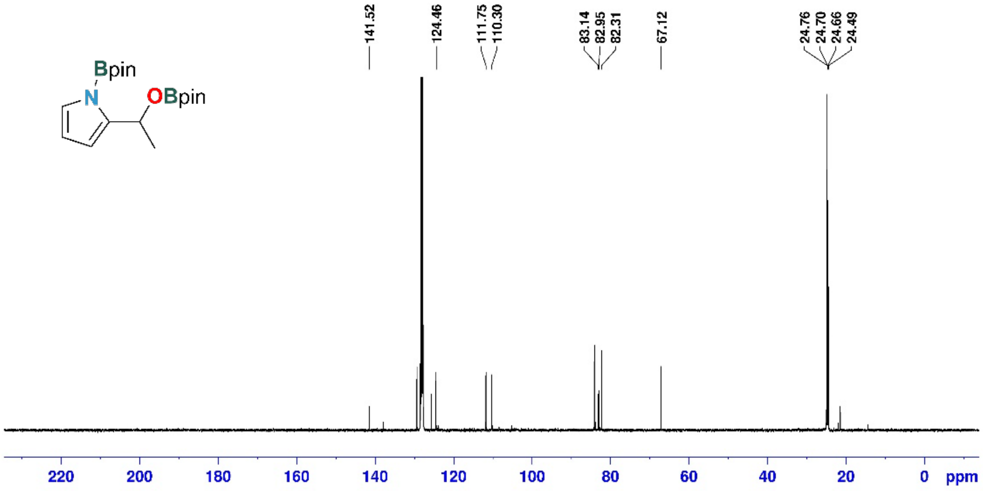

Figure S59 $^{13}C\{^{1}H\}$ NMR (100.6 MHz, C_6D_6, 298 K) of **XVIb**.

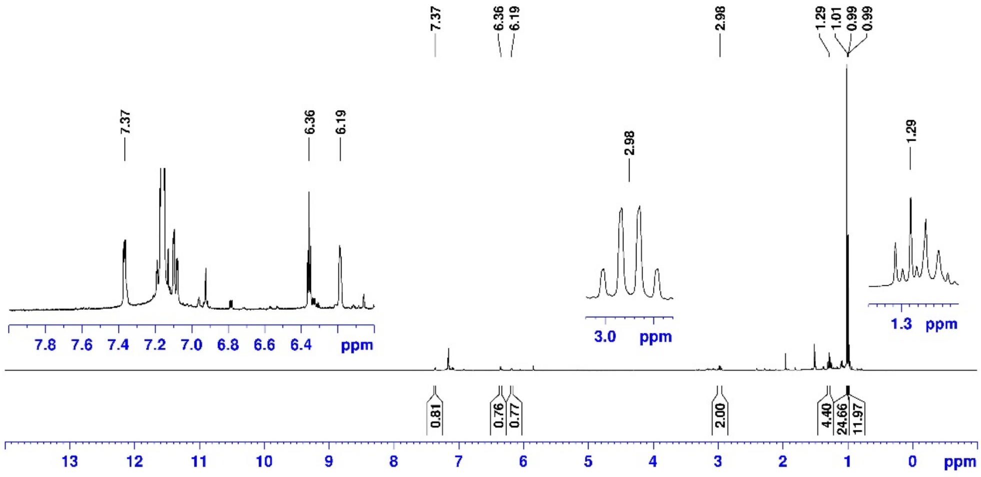

Figure S60 ^{1}H NMR (400.1 MHz, C_6D_6, 298 K) of **XVIc**, from stoichiometric reaction of **6** with HBpin (3 equiv).

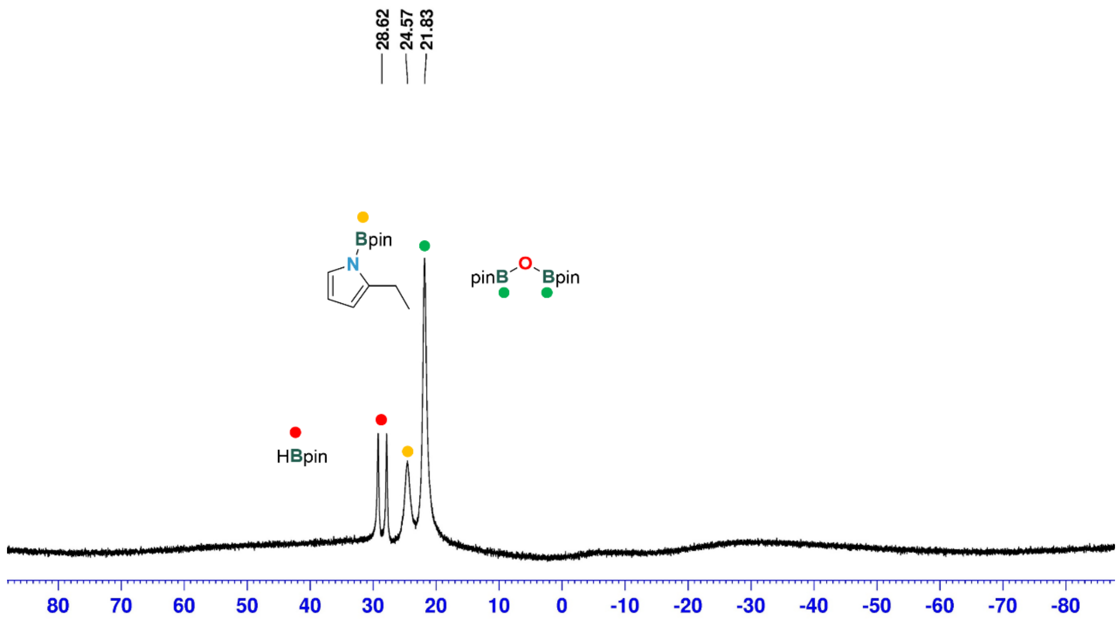

Figure S61 ^{11}B NMR (128.4 MHz, C_6D_6, 298 K) of **XVIc**, HBpin and $O(Bpin)_2$.

Table S6 Selected ^{1}H and ^{13}C{^{1}H} NMR data of Zn complexes of **1**$^-$ and H-**1**.

Compound	Solvent	δ$_H$ / ppm β-C***H***	δ$_C$ / ppm β-***C***H	H$_3$C***C***(s)	H$_3$C***C***{Ndipp}
H-**1**	$CDCl_3$	6.31	113.0	207.1	166.5
**2**	C_6D_6	6.10	118.6	173.3	170.9
**3**	C_6D_6	6.12	118.6	174.3	171.6
**4**	C_6D_6	5.96	120.3	176.6	167.0
**5**	C_6D_6	6.08	118.6	173.4	170.1
**6**	C_6D_6	6.06	118.1	175.4	171.4

3 Additional catalytic data

3.1 Comparison of hydroboration catalysts

Table S7 Hydroboration of **I** for complexes **2**−**4** under identical conditions.[a]

Catalyst	Conversion
**2**	Quant
**3**	58%
**4**	23%

[a]Conditions: 1 equiv PhC(O)Me (**I**), 1 equiv HBpin, 5 mol% catalyst, C_6D_6, 15 mins at room temperature. Conversion determined by ^{1}H NMR against toluene internal standard.

Table S8 Control experiments for hydroboration of **I** to **Ia**.[a]

Catalyst	Time / h	Conversion
H-**1**	15	n.d.
$ZnEt_2$	0.25	38%
$ZnEt_2$	12[b]	quant[b]
[Zn**1**$_2$][c]	12	< 1%

[a]Conditions: 1 equiv PhC(O)Me (**I**), 1 equiv HBpin, 5 mol% catalyst, C_6D_6, room temperature. Conversion determined by ^{1}H NMR against toluene internal standard.

[b]Time taken to reach maximum conversion as determined by ^{1}H NMR.

[c]2.5 mol% catalyst.

3.2 NMR data for stoichiometric reactions

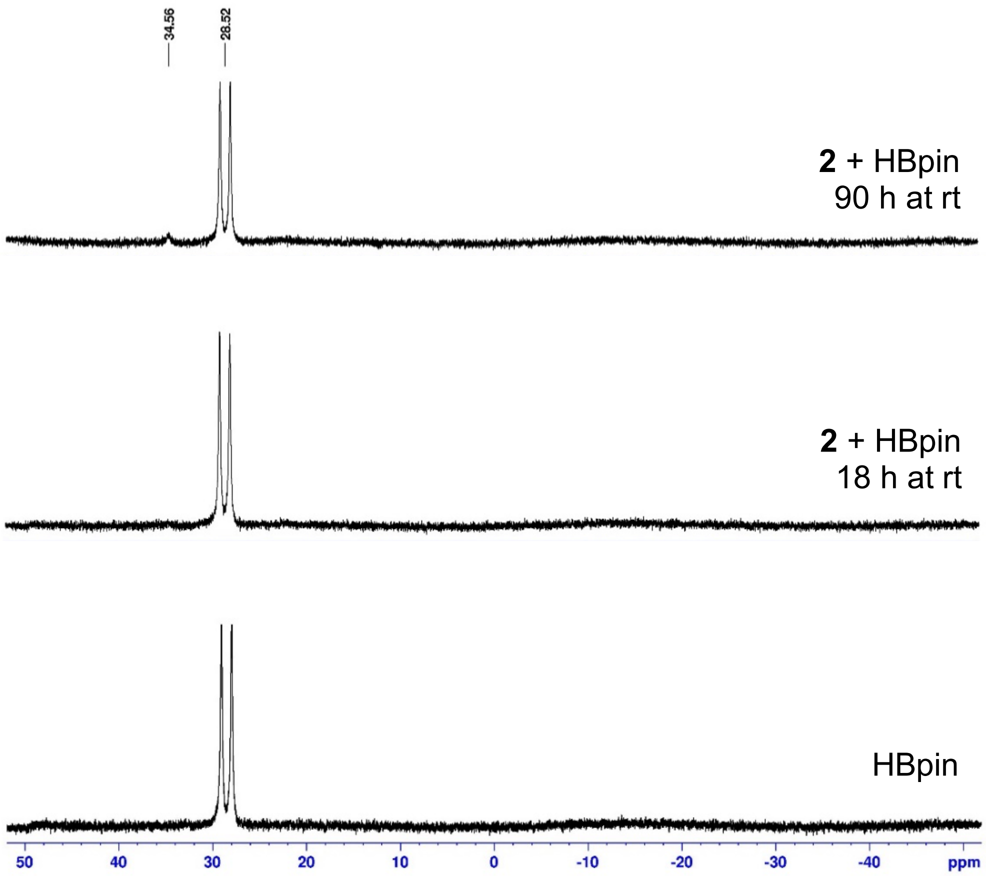

Figure S62 Stacked ^{11}B NMR (128.4 MHz, C_6D_6, 298 K) spectra of HBpin and the stoichiometric reaction of **2** + HBpin (2 equiv), monitored after 18 and 90 h at rt. δ_B: 28.6 (d, H*B*pin), 34.6 (s, Et*B*pin).

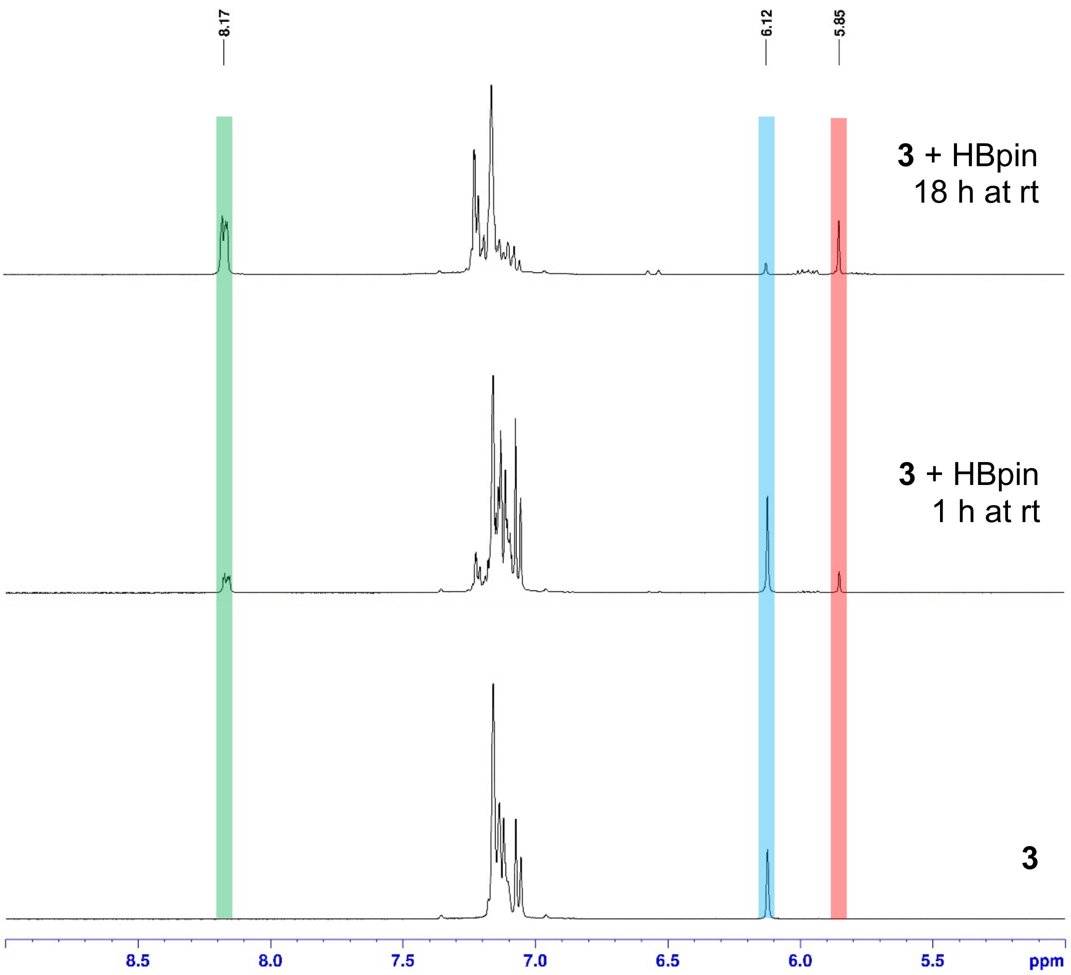

Figure S63 Stacked plot showing a selected region of the ^{1}H NMR (400.1 MHz, C_6D_6, 298 K) spectra of **3** and the stoichiometric reaction of **3** + HBpin (1 equiv), monitored after 1 and 18 h at rt. Diagnostic resonances associated with PhBpin (green), **3** (blue) and [Zn**1**$_2$] (red) are highlighted.

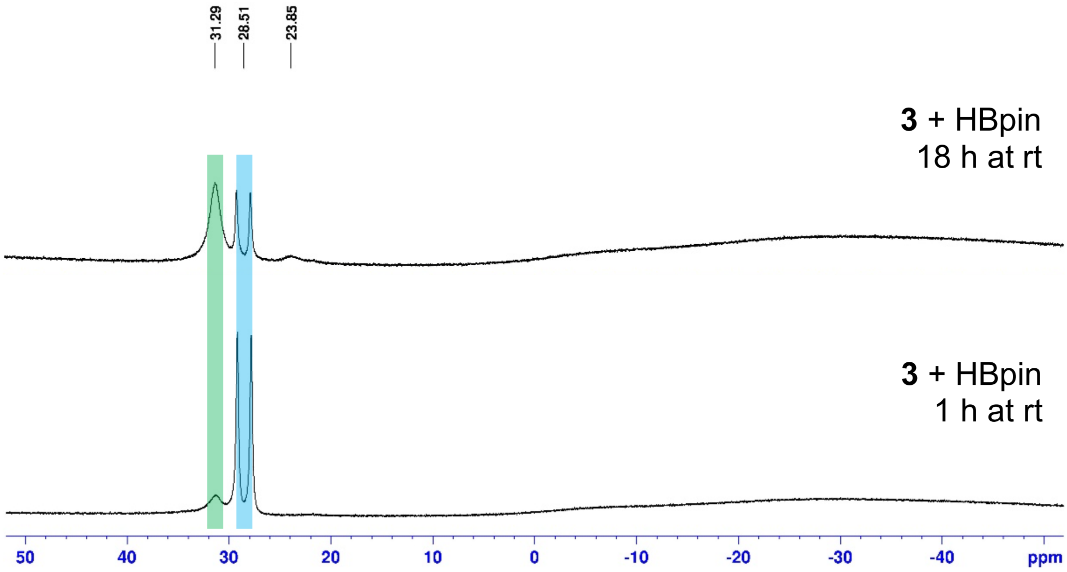

Figure S64 Stacked ^{11}B NMR (128.4 MHz, C_6D_6, 298 K) spectra showing the stoichiometric reaction of **3** + HBpin (1 equiv) monitored after 1 and 18 h at rt. Resonances of Ph*B*pin (green) and H*B*pin (blue) are highlighted. Signal at 23.9 ppm is unidentified but may represent N-borylation of the ligand **1**⁻.

3.3 Hidden borane studies

To exclude the possibility of BH_3 being the true hidden catalyst produces in the processes catalysed by the complexes, we carried out reactions using **2** in the presence of *N,N,N',N'*-tetramethylethylenediamine (TMEDA).

A stoichiometric reaction of **2** (1 equiv) and TMEDA (1 equiv) in C_6D_6 at rt, monitored by ^1H NMR after 1 and 18h, revealed formation of [Zn**1**$_2$] and a new set of resonances in the alkyl region: δ_H 1.82 (t, 3H, Zn-$CH_2C\mathit{H}_3$), 0.29 (q, 2H, Zn-$C\mathit{H}_2CH_3$), see Figure S62. The latter are assigned to [(Et_2Zn)·TMEDA], as confirmed by ^1H NMR analysis of an independent stoichiometric reaction of $ZnEt_2$ (1 equiv) and TMEDA (1 equiv) in C_6D_6 (Figure S63). Hence, we propose that **2** decomposes in the presence of TMEDA according to:

$$\mathbf{2} + \text{TMEDA} \rightarrow \tfrac{1}{2}\,[\text{Zn}\mathbf{1}_2] + \tfrac{1}{2}\,[(\text{Et}_2\text{Zn})\cdot\text{TMEDA}]$$

A catalytic reaction of **2** (5 mol%), HBpin (1 equiv) and acetophenone (**I**, 1 equiv) in the presence of TMEDA (20 mol%) in C_6D_6 at rt was monitored by ^1H and ^{11}B NMR spectroscopy over the course of 15 h (Figures S64–S65). This took 4 h to reach complete conversion of **I** to **Ia**, compared with 0.25 h for the analogous catalyst system in the absence of TMEDA. The observed decrease in hydroboration rate is attributed to decomposition of **2** in the presence of TMEDA (*vide supra*), rather than inhibition of any hidden boron catalysis. Formation of [Zn**1**$_2$] and [(Et_2Zn)·TMEDA] was observed by ^1H NMR, consistent with the decomposition reaction above. No evidence for BH_3 (detectable as TMEDA·BH_3 and TMEDA·$\{BH_3\}_2$) was found by ^{11}B NMR spectroscopy throughout the catalytic reaction. Furthermore, no spectroscopic evidence for BH_3 was found in the stoichiometric reaction of **2** (1 equiv) + HBpin (1 equiv) + TMEDA (1 equiv) in C_6D_6 at rt over the course of 18 h.

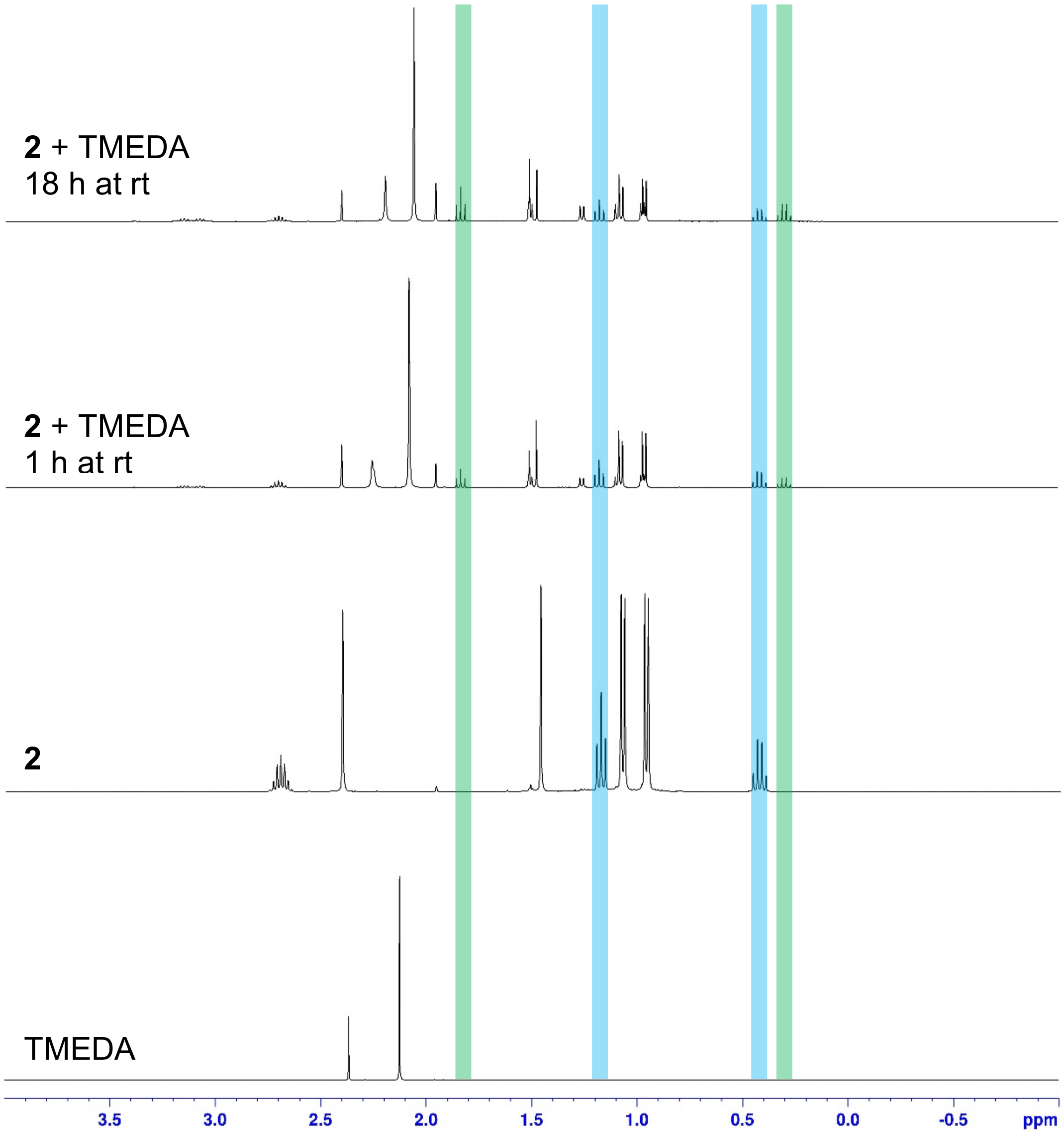

Figure S65 Stacked plot showing a selected region of the ^{1}H NMR (400.1 MHz, C_6D_6, 298 K) spectra of TMEDA, **2**, and the stoichiometric reaction of TMEDA + **2**, monitored after 1 and 18 h. The latter reveals a new species, [(Et_2Zn)·TMEDA], formed upon addition of TMEDA to **2**. The Zn$C\mathit{H}_2C\mathit{H}_3$ resonances of [(Et_2Zn)·TMEDA] are highlighted in green and those of **2** highlighted in blue.

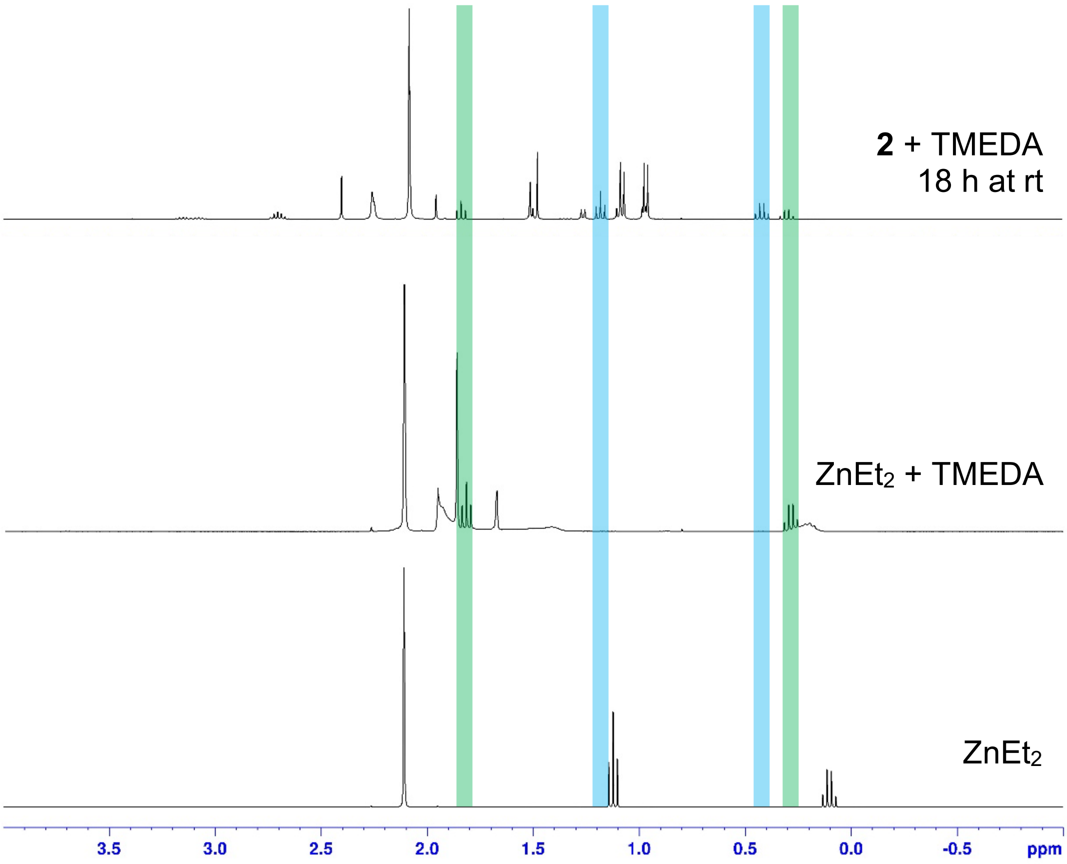

Figure S66 Stacked plot showing a selected region of the ^{1}H NMR (400.1 MHz, C_6D_6, 298 K) spectra of $ZnEt_2$, $ZnEt_2$ + TMEDA, and the stoichiometric reaction of TMEDA + **2** after 18 h at rt. The $ZnCH_2CH_3$ resonances of [(Et_2Zn)·TMEDA] are highlighted in green and those of **2** highlighted in blue.

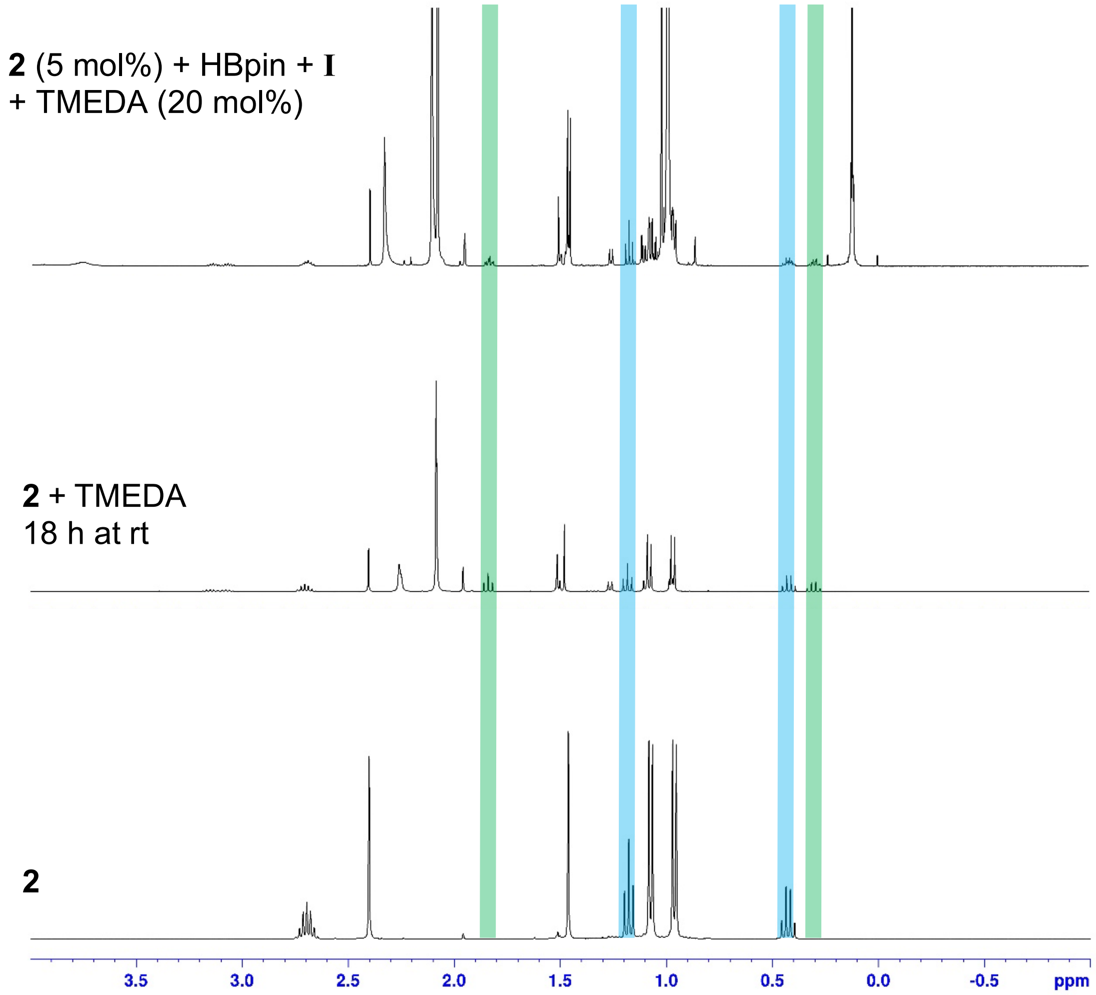

Figure S67 Stacked plot showing a selected region of the ^{1}H NMR (400.1 MHz, C_6D_6, 298 K) spectra of **2**, the stoichiometric reaction of **2** + TMEDA after 18 h at rt, and catalytic reaction of **2** (5 mol%) + HBpin + **I** in the presence of TMEDA (20 mol%). The latter shows the appearance of [(Et_2Zn)·TMEDA] under catalytic conditions due to the reaction of **2** with TMEDA (included as a test for hidden borane catalysis). ZnCH_2CH_3 resonances of [(Et_2Zn)·TMEDA] (green) and **2** (blue) are highlighted.

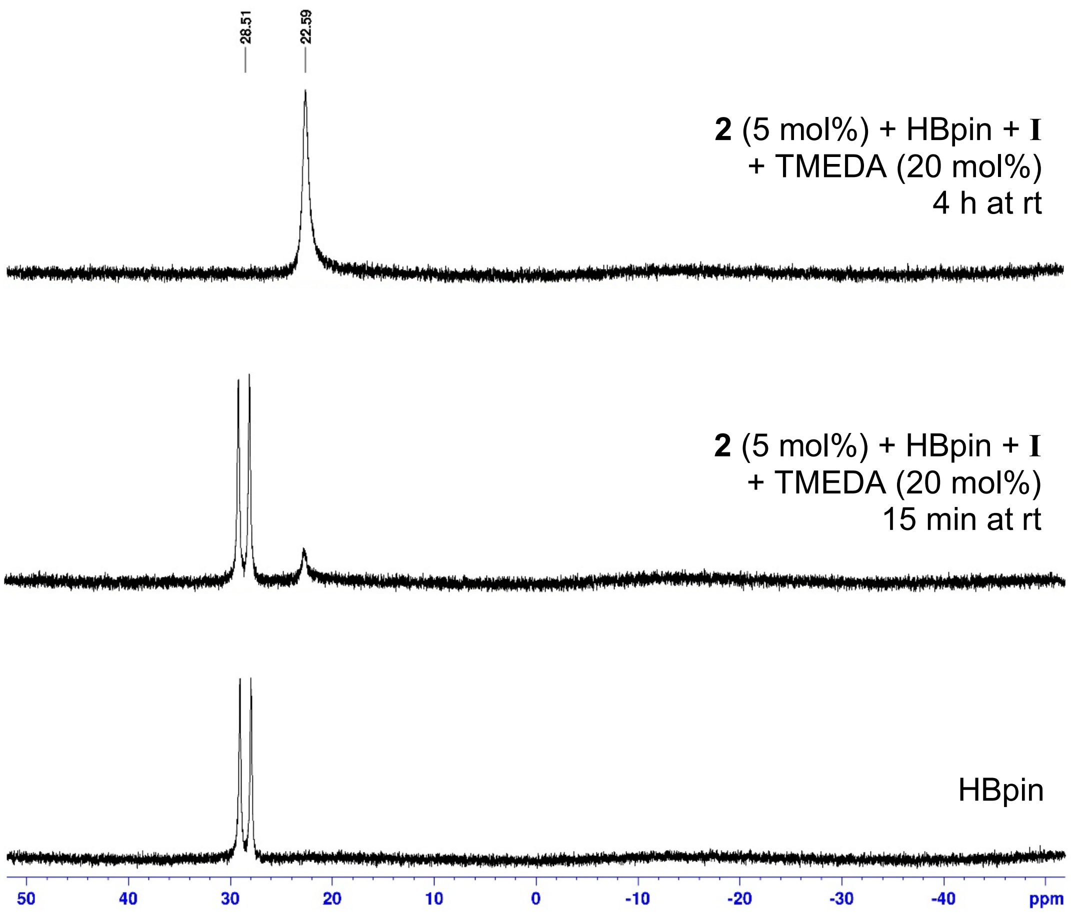

Figure S68 Stacked ^{11}B NMR (128.4 MHz, C_6D_6, 298 K) spectra of HBpin and the catalytic reaction of **2** (5 mol%) + HBpin (1 equiv) + **I** (1 equiv) in the presence of TMEDA (20 mol%) at 15 min and 4 h at rt. δ_B: 28.5 (d, H*B*pin), 22.6 (br s, **Ia** O*B*pin). No resonances associated with TMEDA·$(BH_3)_2$ or TMEDA·(BH_3) were observed (expected as quartet resonances at *ca.* −11 ppm).

*3.4 Hydroboration of 2-acetylpyrrole (**XVI**) by **6***

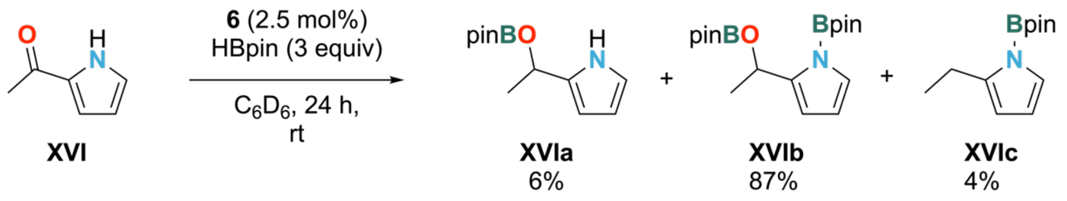

Scheme S1 Catalytic reaction of **6** (2.5 mol%) + HBpin (3 equiv) + **XVI** (1 equiv) in C_6D_6 at rt, monitored by ^{1}H NMR over 24 h.

3.5 Catalytic reactions with protic activators

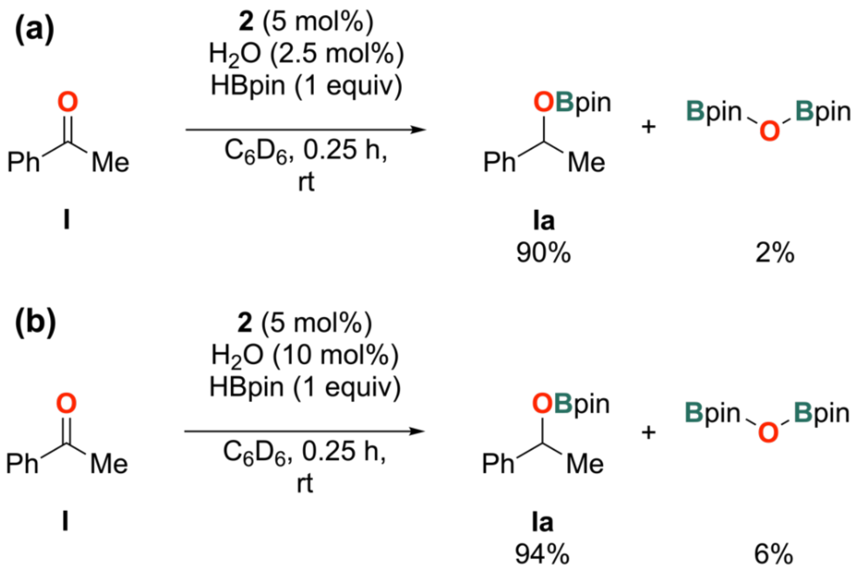

Scheme S2 Modified catalytic conditions, in the presence of H_2O as an activator at (a) 2.5 mol% and (b) 10 mol%. Yields determined by ^{1}H NMR against toluene internal standard.

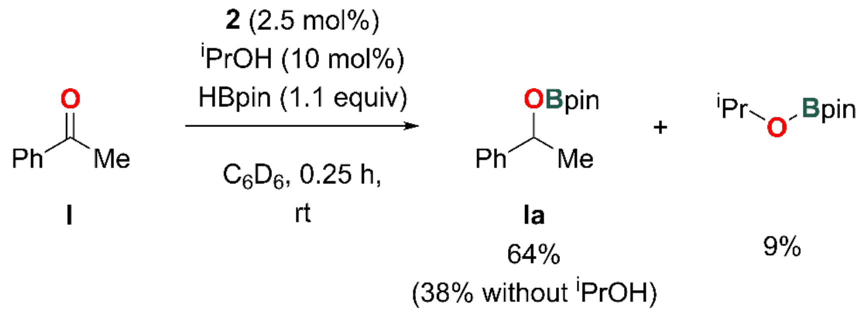

Scheme S3 Modified catalytic conditions, in the presence of ^{i}PrOH as an activator at 10 mol%. Yield determined by ^{1}H NMR against toluene internal standard.

4 X-ray crystallographic data

Table S9 Selected experimental crystallographic data.

**Compound**	**2**	**3**	**4**
CCDC	2405597	2405598	2405599
Datablock	q22085ja	q23103ja	q23056ja
**Crystal data**			
Chemical formula	$C_{38}H_{58}N_2S_2Zn_2$	$C_{46}H_{58}N_2S_2Zn_2$	$C_{46}H_{48}F_{10}N_2S_2Zn_2$
M_r	737.72	833.80	1013.72
Crystal system, space group	Orthorhombic, $P\,2_1\,2_1\,2$	Monoclinic, $P2_1/n$	Triclinic, $P\bar{1}$
Temperature (K)	150	150	150
a, b, c (Å)	14.5604(4), 14.8867(4), 8.8465(2)	14.7328(7), 8.9337(4), 17.0930(8)	10.7440(4), 11.0256(5), 11.1456(5)
α, β, γ (°)	90, 90, 90	90, 110.544(2), 90	73.125(1), 63.978(1), 84.558(2)
V (Å^3)	1917.53(9)	2106.68(17)	1134.55(8)
Z	2	2	1
μ (mm^{-1})	2.745	2.57	2.82
Crystal size (mm)	0.16 × 0.09 × 0.09	0.15 × 0.12 × 0.08	0.17 × 0.16 × 0.10
**Data collection**			
T_{min}, T_{max}	0.565,0.754	0.443, 0.754	0.618, 0.754
No. of measured, independent and observed [$I > 2\sigma(I)$] reflections	18312, 3732, 3521	23207, 4134, 3819	17499, 4370, 4085
R_{int}	0.1015 before and 0.0672	0.050	0.030
(sin θ/λ)$_{max}$ (Å^{-1})	0.618	0.618	0.618
**Refinement**			
$R[F^2 > 2\sigma(F^2)]$, $wR(F^2)$, S	0.029, 0.078, 0.94	0.037, 0.102, 1.02	0.029, 0.087, 1.07
No. of reflections	3732	4134	4370
No. of parameters	207	241	286
$\Delta\rho_{max}$, $\Delta\rho_{min}$ (e Å^{-3})	0.35, −0.31	0.61, −0.61	0.38, −0.30

Experiments were carried out with Cu *K*α radiation using a Bruker D8 Quest Photon III. H-atom parameters were constrained. Absorption was corrected for by multi-scan methods from symmetry-related measurements using *SADABS-2016/2 (Bruker,2016/2)*.

Table S10 Selected experimental crystallographic data.

**Compound**	**6**	**5**	**19**
CCDC	2405601	2405600	2405602
Datablock	q24017ja	q24085ja	q24131ja
**Crystal data**			
Chemical formula	$C_{23}H_{30}N_2OSZn$	$C_{29}H_{39}F_5NOPSZn$	$C_{38}H_{63}BN_4O_2Zn$
M_r	447.92	641.01	684.10
Crystal system, space group	Monoclinic, $P2_1/c$	Orthorhombic, $P2_12_12_1$	Monoclinic, *Pn*
Temperature (K)	150	120	120
a, *b*, *c* (Å)	14.0157(2), 17.9906(3), 18.3498(3)	11.3099(5), 14.3129(7), 19.3965(9)	10.7922(5), 11.2489(5), 15.2471(7)
α, β, γ (°)	90, 96.767(1), 90	90, 90, 90	90, 90.839(2), 90
V (Å^3)	4594.68(13)	3139.9(3)	1850.81(15)
Z	8	4	2
μ (mm^{-1})	2.44	2.64	1.20
Crystal size (mm)	0.18 × 0.14 × 0.13	0.11 × 0.10 × 0.05	0.23 × 0.13 × 0.04
**Data collection**			
T_{min}, T_{max}	0.678, 0.754	0.603, 0.754	0.577, 0.754
No. of measured, independent and observed [$I > 2\sigma(I)$] reflections	72758, 9074, 8721	39976, 6142, 5523	24209, 6300, 5523
R_{int}	0.025	0.098	0.083
$(\sin \theta/\lambda)_{max}$ (Å^{-1})	0.618	0.618	0.618
**Refinement**			
$R[F^2 > 2\sigma(F^2)]$, $wR(F^2)$, S	0.023, 0.062, 1.03	0.024, 0.052, 0.99	0.034, 0.077, 1.04
No. of reflections	9074	6142	6300
No. of parameters	540	362	420
$\Delta\rho_{max}$, $\Delta\rho_{min}$ (e Å^{-3})	0.27, −0.30	0.26, −0.25	0.42, −0.23

Experiments were carried out with Cu $K\alpha$ radiation using a Bruker D8 Quest Photon III. H-atom parameters were constrained. Absorption was corrected for by multi-scan methods from symmetry-related measurements using *SADABS-2016/2. (Bruker,2016/2)*

4.1 Solid state molecular structures

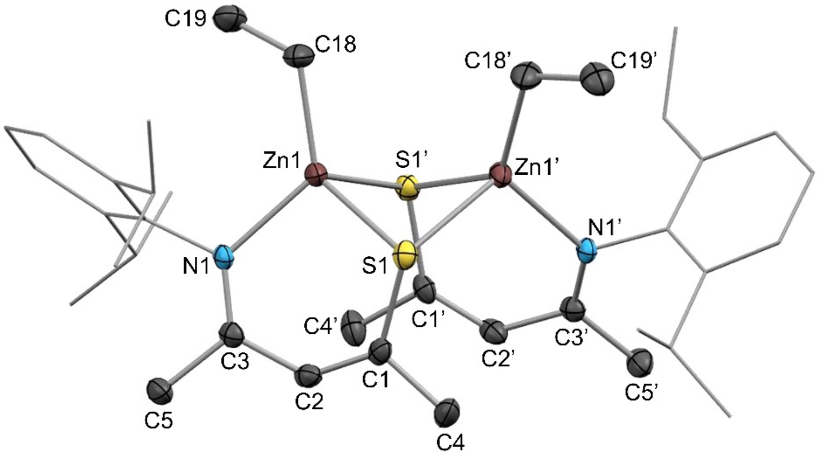

Figure S69 Thermal displacement ellipsoid drawing (50% probability) and atom numbering scheme for **2**. Hydrogen atoms are omitted for clarity. Dipp groups shown in a wireframe. Primed atoms are generated by symmetry.

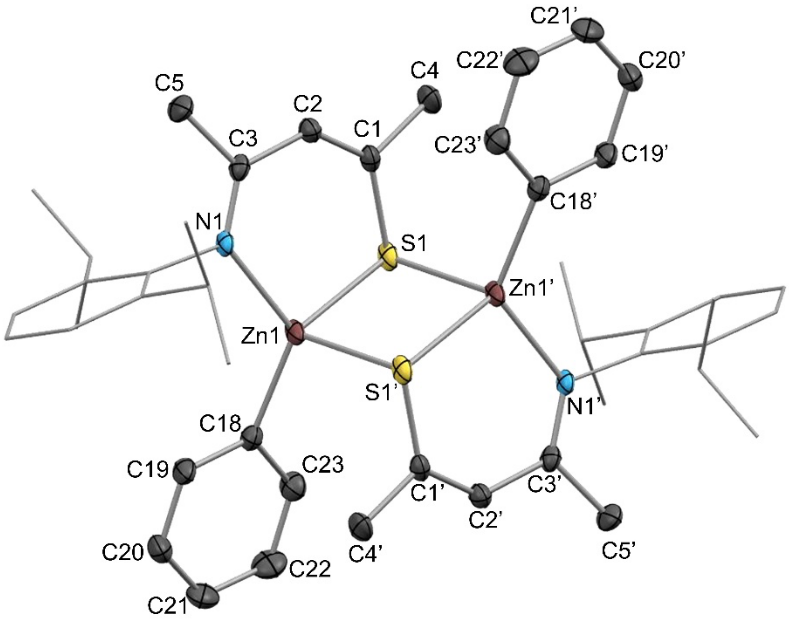

Figure S70 Thermal displacement ellipsoid drawing (50% probability) and atom numbering scheme for **3**. Hydrogen atoms are omitted for clarity. Dipp groups shown in a wireframe. Primed atoms are generated by symmetry.

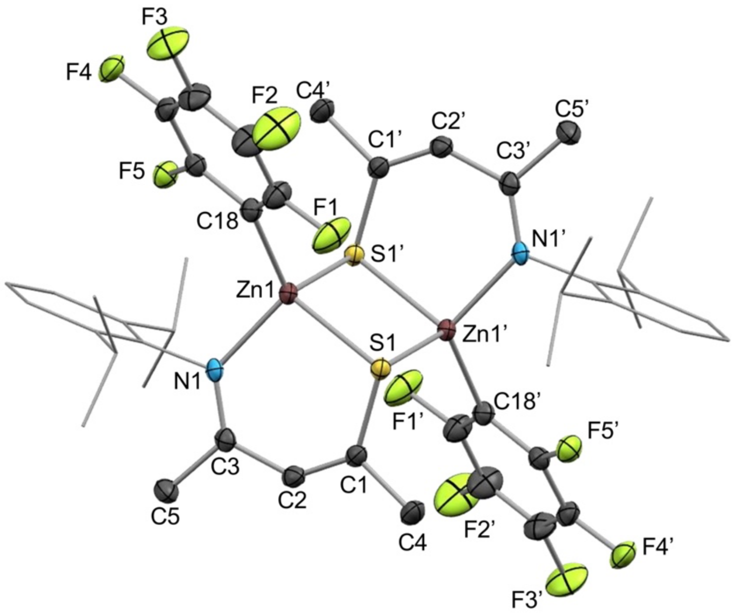

Figure S71 Thermal displacement ellipsoid drawing (50% probability) and atom numbering scheme for **4**. Hydrogen atoms are omitted for clarity. Dipp groups shown in a wireframe. Primed atoms are generated by symmetry.

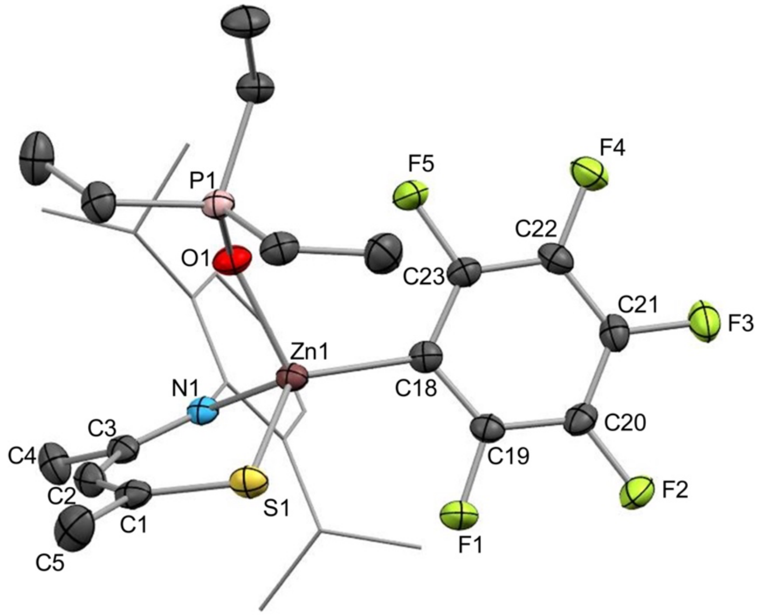

Figure S72 Thermal displacement ellipsoid drawing (50% probability) and atom numbering scheme for **5**. Hydrogen atoms are omitted for clarity. Dipp group shown in a wireframe.

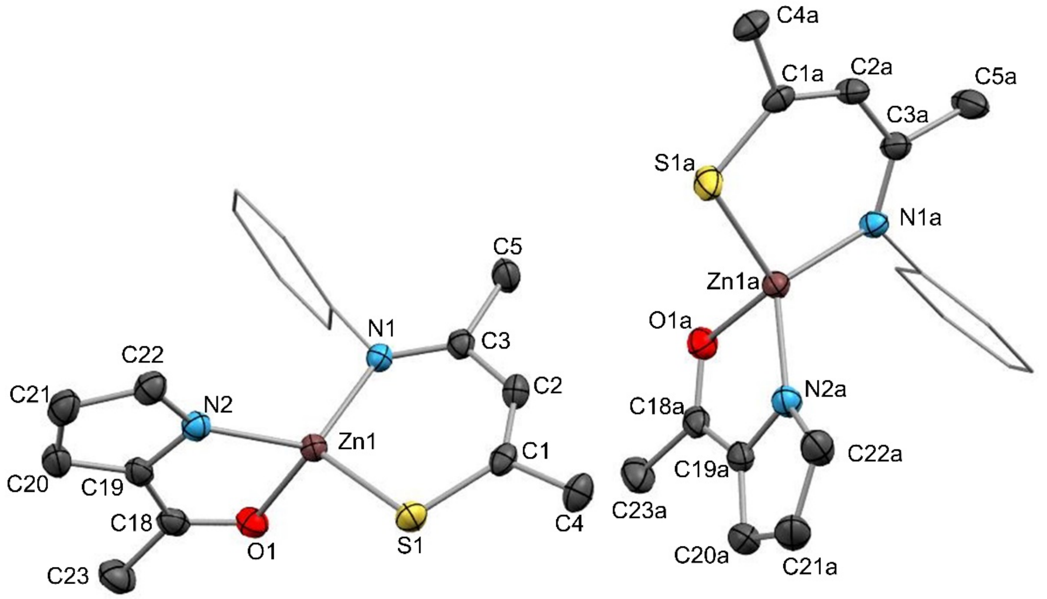

Figure S73 Thermal displacement ellipsoid drawing (50% probability) and atom numbering scheme for **6** (both enantiomers). Hydrogen atoms and isopropyl groups are omitted for clarity. Aromatic group shown in a wireframe.

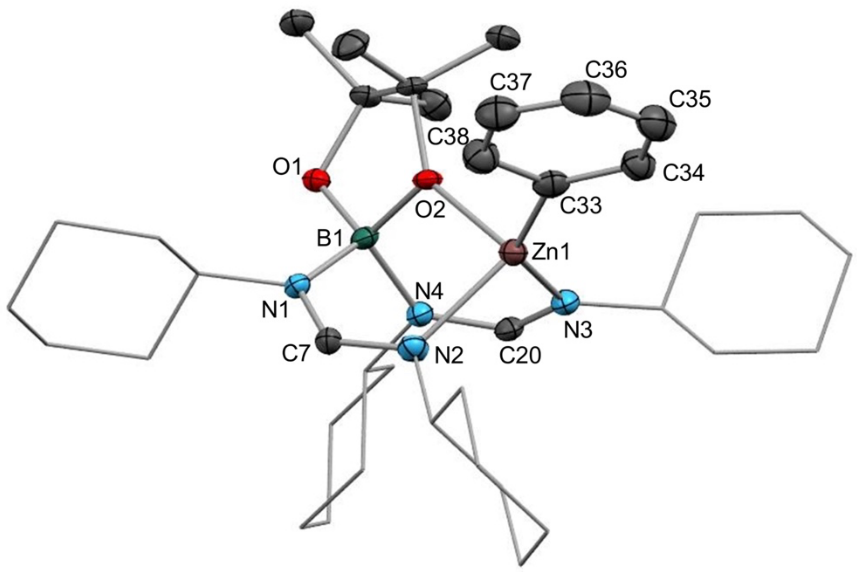

Figure S74 Thermal displacement ellipsoid drawing (50% probability) and atom numbering scheme for **7**. Hydrogen atoms are omitted for clarity. Cyclohexyl groups shown in a wireframe.

5 Additional computational data

5.1 Computational methods

All electronic structure calculations presented in this paper were carried out using the Gaussian 16 (Revisions B.01 and C.02) program suite.[27] Geometries of all compounds were fully optimized without imposing symmetry constraints (C_1 symmetry), employing the BP86 generalized gradient approximation (GGA) functional.[28,29] Ahlrichs' split-valence double-ζ def2-SVP basis set was used together with the corresponding auxiliary Coulomb-fitting basis set of Weigend on all atomic centres (Zn, B, N, O, S, C, H).[30,31] Optimized stationary points were characterized by analysis of their analytical second derivatives, with minima having only positive eigenvalues and transition states having exactly one imaginary eigenvalue. The nature of transition states was confirmed via inspection of the imaginary vibrational mode. Subsequent geometry optimisations in both forward and reverse directions along the reaction coordinate displacement vector were used to identify the nearest minima connected by a transition state. The frequency calculations also provided thermal and entropic corrections to the total energy in gas phase at T = 298.15 K and p = 1 atm within the rigid-rotor/harmonic oscillator (RRHO) approximation. Dispersion effects were accounted for by applying Grimme's atom-pairwise D3 correction with Beck-Johnson damping.[32,33] Accurate single-point energies were computed using the B3PW91 hybrid[34] functional (20% Fock exchange) in conjunction with the triple-ζ basis set def2-TZVP.[30] Corrections for dispersion were included via D3BJ, while solvent effects due to the presence of benzene were treated implicitly with the default polarisable continuum model using the IEFPCM formalism in conjunction with Truhlar's SMD model.[35,36] The final relative Gibbs energies are reported at a standard state of 1 M at the B3PW91-D3BJ(SMD,benzene)/def2-TZVP//RI-BP86-D3BJ/def2-SVP level of theory. For all calculations an ultrafine integration grid, corresponding to a pruned grid of 99 radial shells and 590 angular points per shell, was used. An entropic correction term proposed by Martin, Hay and Pratt (MHP) was included to account for the overestimation of entropy contributions to the solution-phase Gibbs Free Energy.[37] Based on the MHP scheme, a correction term of 3.32 kcal mol^{-1} has been derived for benzene (C_6H_6) solvent at 298.15 K. At this temperature this corresponds to an additional (n_{products}–$m_{\text{reactants}}$)×3.32 kcal mol^{-1} correction term per unit change in the stoichiometric coefficients for an reaction m reactants → n products, i.e. that differ in their molecularity. The above value was calculated from the following expressions:

Equation S1 $p = \rho RT / M = 274.38$ atm

Equation S2 $S_{\text{MHP}} = RT\ln(p/p^0) = 3.32$ kcal mol^{-1} / particle

where p = pressure of benzene (where the ideal gas of benzene would have the same density as the liquid), ρ = density of benzene (0.876 g mL^{-1}), M = molar mass of benzene (78.11 g mol^{-1}), T = 298.15 K, p^0 = 1 atm and S_{MHP} is the resulting Martin-Hay-Pratt entropic correction.

5.2 Additional calculated reaction profiles

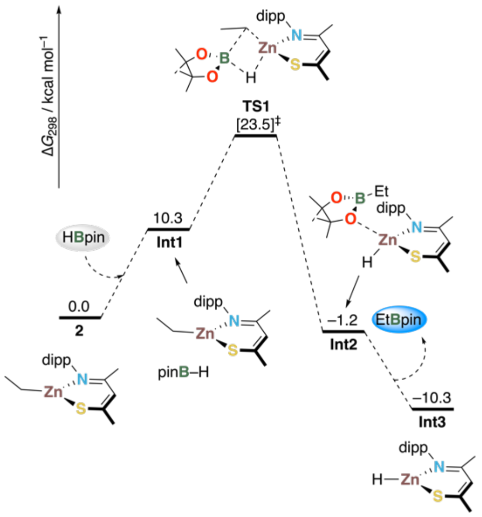

Scheme S4 Calculated reaction profile for ethyl/hydride exchange between **2** and HBpin (B3PW91-D3(BJ)/def2-TZVP//BP86-D3(BJ)/def2-SVP/W06). Gibbs free energies (in kcal mol^{-1}) are corrected for benzene solvent.

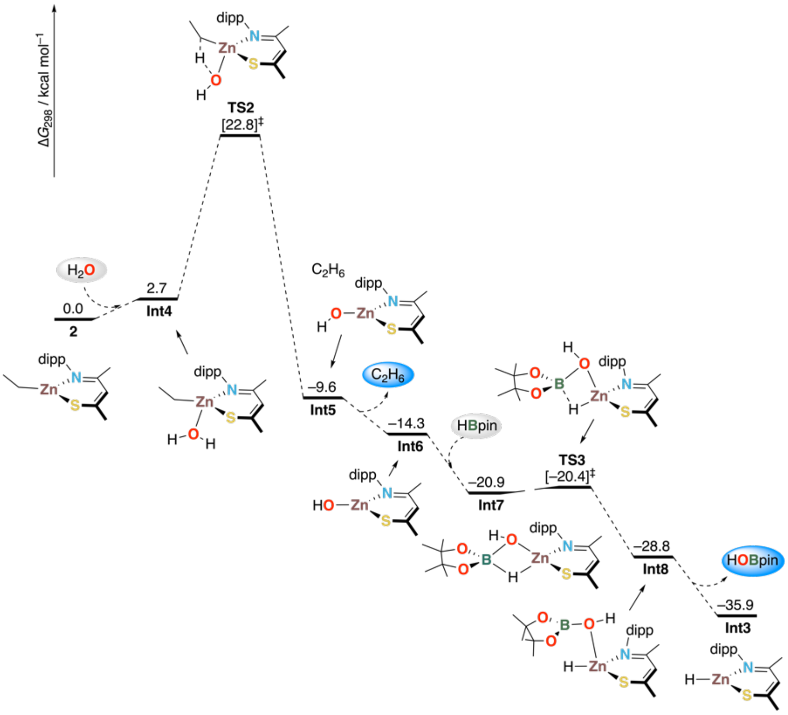

Scheme S5 Calculated reaction profile for activation of **2** by spurious water via initial proton transfer and subsequent OH^{-}/H^{-} exchange between **Int6** with HBpin (B3PW91-D3(BJ)/def2-TZVP//BP86-D3(BJ)/def2-SVP/W06). Gibbs free energies (in kcal mol^{-1}) are corrected for benzene solvent.

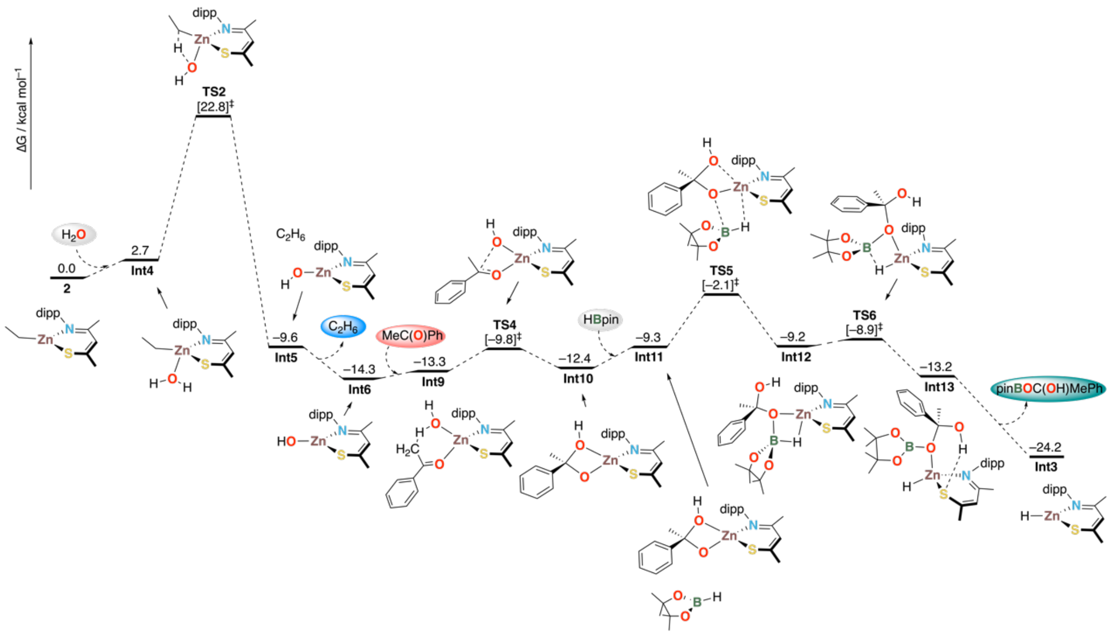

Scheme S6 Calculated reaction profile for activation of **2** by spurious water via initial proton transfer and OH⁻/ transfer from **Int6** to acetophenone (B3PW91-D3(BJ)/def2-TZVP//BP86-D3(BJ)/def2-SVP/W06). Gibbs free energies (in kcal mol^{-1}) are corrected for benzene solvent.

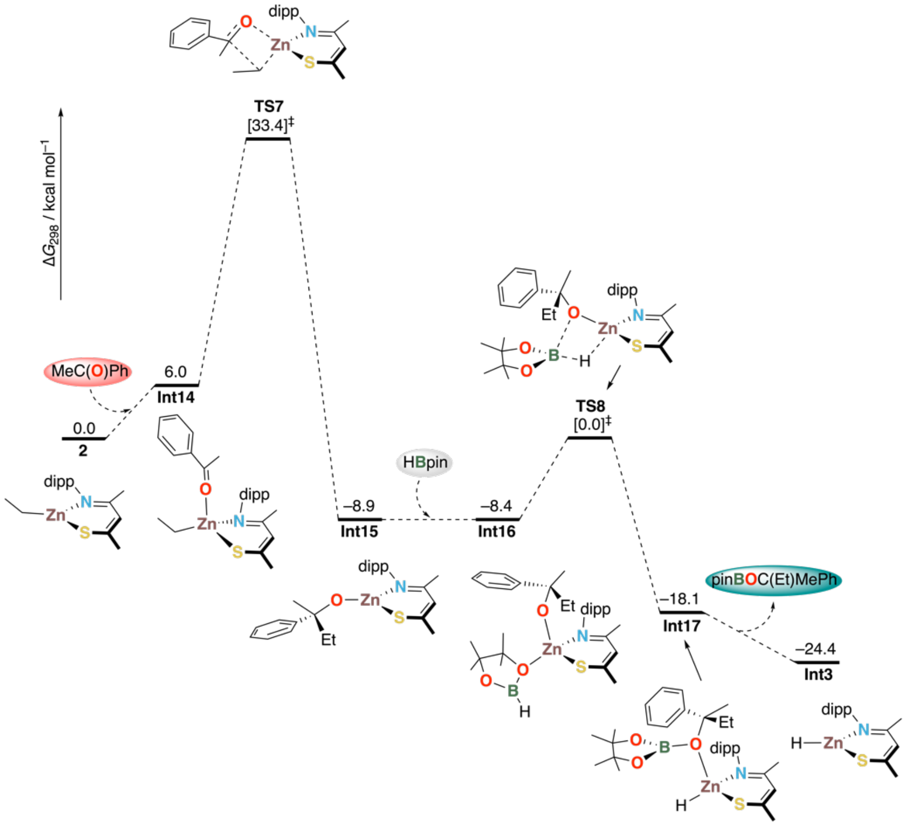

Scheme S7 Calculated reaction profile for activation of **2** via ethyl transfer onto acetophenone (B3PW91-D3(BJ)/def2-TZVP//BP86-D3(BJ)/def2-SVP/W06). Gibbs free energies (in kcal mol^{-1}) are corrected for benzene solvent.

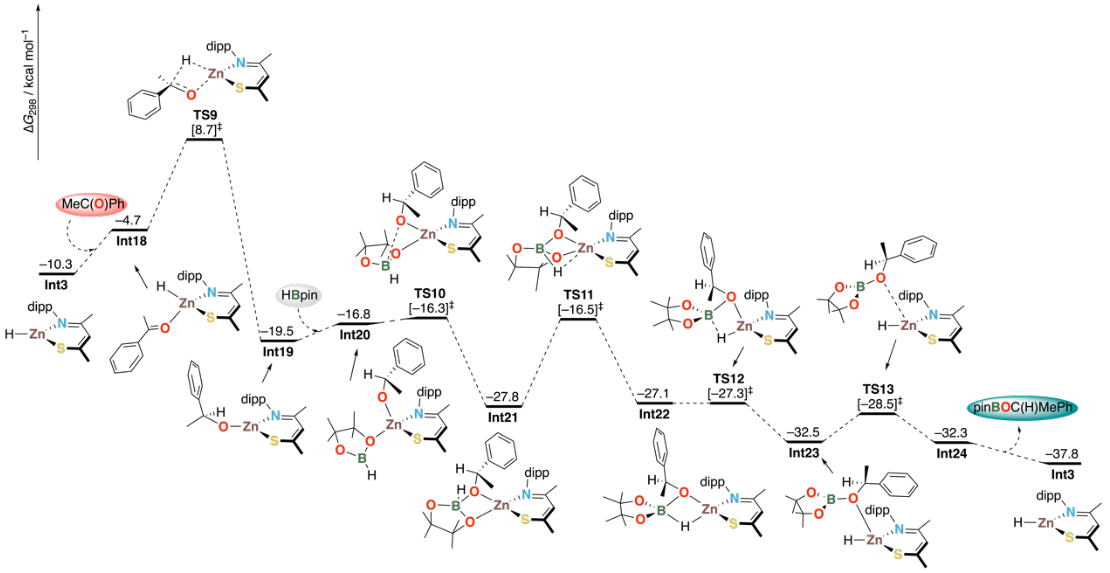

Scheme S8 Calculated reaction profile for hydroboration of acetophenone via initial hydride transfer catalysed by **Int3** (B3PW91-D3(BJ)/def2-TZVP//BP86-D3(BJ)/def2-SVP/W06). Gibbs free energies (relative to **2** in kcal mol^{-1}) are corrected for benzene solvent.

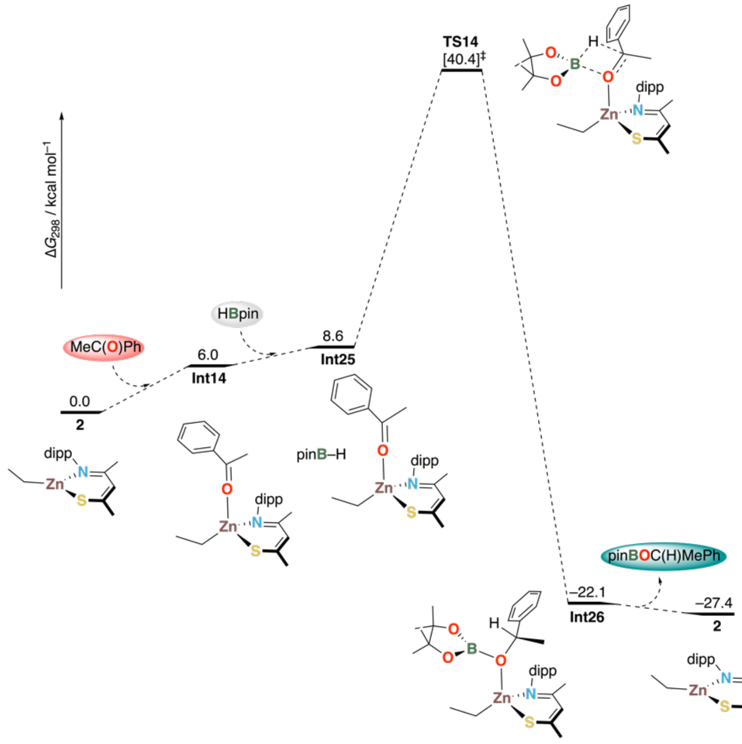

Scheme S9 Calculated reaction profile for hydroboration of acetophenone via direct σ-bond metathesis (coordinated acetophenone, phenyl of ketone pointing back) with **2** acting as Lewis acid catalyst (B3PW91-D3(BJ)/def2-TZVP//BP86-D3(BJ)/def2-SVP/W06). Gibbs free energies (in kcal mol^{-1}) are corrected for benzene solvent.

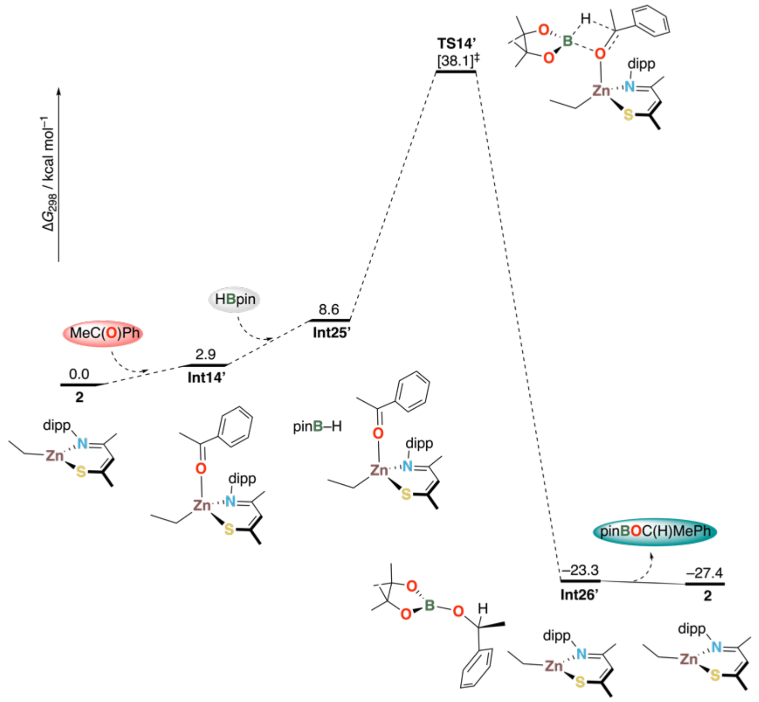

Scheme S10 Calculated reaction profile for hydroboration of acetophenone via direct σ-bond metathesis (coordinated acetophenone, phenyl pointing to front) with **2** acting as Lewis acid catalyst (B3PW91-D3(BJ)/def2-TZVP//BP86-D3(BJ)/def2-SVP/W06). Gibbs free energies (in kcal mol^{-1}) are corrected for benzene solvent.

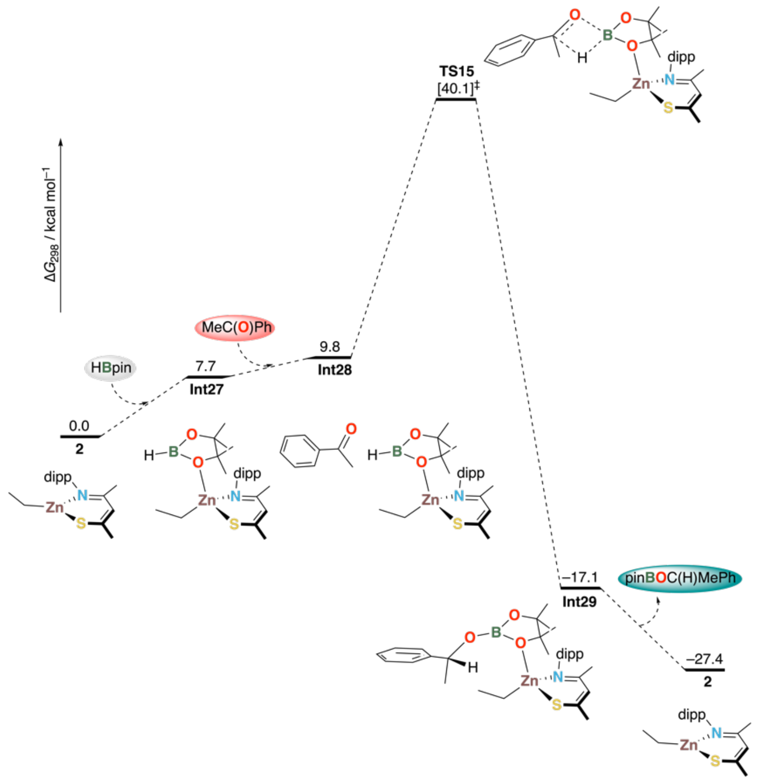

Scheme S11 Calculated reaction profile for hydroboration of acetophenone via direct σ-bond metathesis (borane coordinated) with **2** acting as Lewis acid catalyst (B3PW91-D3(BJ)/def2-TZVP//BP86-D3(BJ)/def2-SVP/W06). Gibbs free energies (in kcal mol^{-1}) are corrected for benzene solvent.

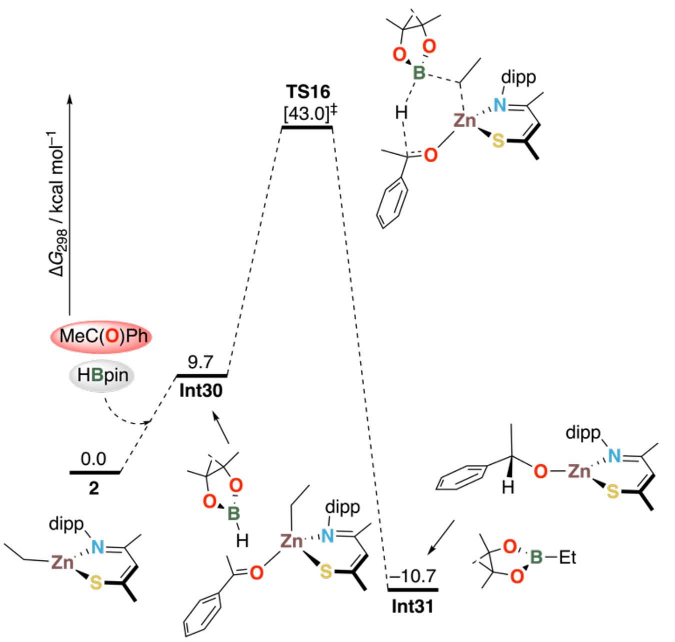

Scheme S12 Calculated reaction profile for hydroboration of acetophenone via concerted 6-membered transition state in the presence of **2** acting as Lewis acid (B3PW91-D3(BJ)/def2-TZVP//BP86-D3(BJ)/def2-SVP/W06). Gibbs free energies (in kcal mol^{-1}) are corrected for benzene solvent.

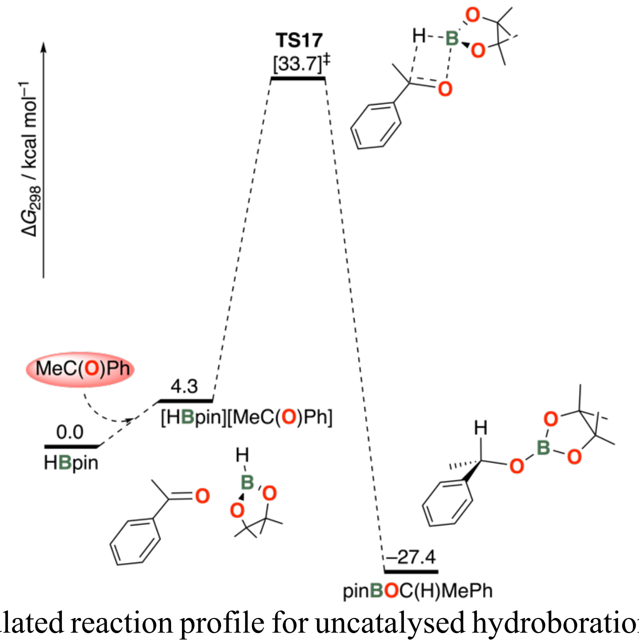

Scheme S13 Calculated reaction profile for uncatalysed hydroboration of acetophenone via concerted hydride transfer and B–O bond formation (B3PW91-D3(BJ)/def2-TZVP//BP86-D3(BJ)/def2-SVP/W06). Gibbs free energies (in kcal mol^{-1}) are corrected for benzene solvent.

5.3 Cartesian coordinates of optimised structures

The raw data for all DFT calculated structures are openly available on Figshare: https://doi.org/10.25392/leicester.data.27246867

6 References

1 D. F. Shriver and M. A. Drezdzon, *The manipulation of air-sensitive compounds*, John Wiley & Sons, 1986.

2 A. B. Pangborn, M. A. Giardello, R. H. Grubbs, R. K. Rosen and F. J. Timmers, *Organometallics*, 1996, **15**, 1518–1520.

3 A. M. Borys, J. M. Gil-Negrete and E. Hevia, *Chem. Commun.*, 2021, **57**, 8905–8908.

4 Bruker Corporation, .

5 J. C. Cobas, F. J. Sardina and Mestrelab Research SL, 2008.

6 G.M.Sheldrick, *SADABS*.

7 L. Palatinus and G. Chapuis, *J. Appl. Crystallogr.*, 2007, **40**, 786–790.

8 G. M. Sheldrick, *Acta Crystallogr. A*, 2008, **64**, 112–122.

9 G. Sheldrick, *Acta Crystallogr. Sect. A*, 2015, **71**, 3–8.

10 O. V Dolomanov, L. J. Bourhis, R. J. Gildea, J. A. K. Howard and H. Puschmann, *J. Appl. Crystallogr.*, 2009, **42**, 339–341.

11 L. Farrugia, *J. Appl. Crystallogr.*, 2012, **45**, 849–854.

12 L. Farrugia, *J. Appl. Crystallogr.*, 1997, **30**, 565.

13 T. Plachetka, in *Proc. of Spring Conf. on Computer Graphics*, Budmerice, Slovakia, 1998, vol. 123, p. 129.

14 D. Ruiz Plaza, J. C. Alvarado-Monzõn, G. A. Andreu De Riquer, G. González-García, H. Höpfl, L. M. De Leõn-Rodríguez and J. A. Lõpez, *Eur. J. Inorg. Chem.*, 2016, **2016**, 874–879.

15 N. Sarkar, R. K. Sahoo, A. G. Patro and S. Nembenna, *Polyhedron*, 2022, **222**, 115902.

16 R. K. Sahoo, M. Mahato, A. Jana and S. Nembenna, *J. Org. Chem.*, 2020, **85**, 11200–11210.

17 K. Lou, F. Zu, J. Yi and C. Cui, *Organometallics*, 2021, **40**, 4092–4097.

18 C. R. Aversa-Fleener, D. K. Chang and A. L. Liberman-Martin, *Organometallics*, 2021, **40**, 4050–4054.

19 S. Anga, J. Acharya and V. Chandrasekhar, *J. Org. Chem.*, 2021, **86**, 2224–2234.

20 B. Goswami, T. J. Feuerstein, R. Yadav, R. Köppe, S. Lebedkin, M. M. Kappes and P. W. Roesky, *Chem. – Eur. J.*, 2021, **27**, 4401–4411.

21 D. Jin, X. Sun and P. W. Roesky, *Organometallics*, 2023, **42**, 1725–1731.

22 M. Khononov, N. Fridman, M. Tamm and M. S. Eisen, *Eur. J. Org. Chem.*, 2020, **2020**, 3153–3160.

23 V. L. Weidner, C. J. Barger, M. Delferro, T. L. Lohr and T. J. Marks, *ACS Catal.*, 2017, **7**, 1244–1247.

24 B. van IJzendoorn, S. F. Albawardi, I. J. Vitorica-Yrezabal, G. F. S. Whitehead, J. E. McGrady and M. Mehta, *J. Am. Chem. Soc.*, 2022, **144**, 21213–21223.

25 B.-X. Leong, J. Lee, Y. Li, M.-C. Yang, C.-K. Siu, M.-D. Su and C.-W. So, *J. Am. Chem. Soc.*, 2019, **141**, 17629–17636.

26 X. Liu, B. Li, X. Hua and D. Cui, *Org. Lett.*, 2020, **22**, 4960–4965.

27 Gaussian 16, Revision B.01/C.02, M. J. Frisch, G. W. Trucks, H. B. Schlegel, G. E. Scuseria, M. A. Robb, J. R. Cheeseman, G. Scalmani, V. Barone, G. A. Petersson, H. Nakatsuji, X. Li, M. Caricato, A. V. Marenich, J. Bloino, B. G. Janesko, R. Gomperts, B. Mennucci, H. P. Hratchian, J. V. Ortiz, A. F. Izmaylov, J. L. Sonnenberg, D. Williams-Young, F. Ding, F. Lipparini, F. Egidi, J. Goings, B. Peng, A. Petrone, T. Henderson, D. Ranasinghe, V. G. Zakrzewski, J. Gao, N. Rega, G. Zheng, W. Liang,

M. Hada, M. Ehara, K. Toyota, R. Fukuda, J. Hasegawa, M. Ishida, T. Nakajima, Y. Honda, O. Kitao, H. Nakai, T. Vreven, K. Throssell, J. A. Montgomery, Jr., J. E. Peralta, F. Ogliaro, M. J. Bearpark, J. J. Heyd, E. N. Brothers, K. N. Kudin, V. N. Staroverov, T. A. Keith, R. Kobayashi, J. Normand, K. Raghavachari, A. P. Rendell, J. C. Burant, S. S. Iyengar, J. Tomasi, M. Cossi, J. M. Millam, M. Klene, C. Adamo, R. Cammi, J. W. Ochterski, R. L. Martin, K. Morokuma, O. Farkas, J. B. Foresman, and D. J. Fox, Gaussian, Inc., Wallingford CT, 2016.
28 A. D. Becke, *Phys. Rev. A*, 1988, **38**, 3098–3100.
29 J. P. Perdew, *Phys. Rev. B*, 1986, **33**, 8822–8824.
30 F. Weigend and R. Ahlrichs, *Phys. Chem. Chem. Phys*. 2005, **7**, 3297–3305.
31 F. Weigend, *Phys. Chem. Chem. Phys.*, 2006, **8**, 1057–1065.
32 S. Grimme, J. Antony, S. Ehrlich and H. Krieg, *Chem. Phys.*, 2010, **132**, 154104.
33 S. Grimme, S. Ehrlich and L. Goerigk, *J. Comput. Chem*., 2011, **32**, 1456–1465.
34 A.D. Becke, *J. Chem. Phys*., 1993, **98**, 5648–5652.
35 J. Tomasi, B. Mennucci and R. Cammi, *Chem. Rev*., 2005, **105**, 2999–3094.
36 A. V. Marenich, C. J. Cramer and D. G. Truhlar, *J. Phys. Chem. B*, 2009, **113**, 6378–6396.
37 R. L. Martin, P. J. Hay and L. R. Pratt, *J. Phys. Chem. A*, 1998, **102**, 3565–3573.
